# Prevalence of Antibiotic-Resistant Pathogens in Culture-Proven Sepsis and Outcomes Associated With Inadequate and Broad-Spectrum Empiric Antibiotic Use

**DOI:** 10.1001/jamanetworkopen.2020.2899

**Published:** 2020-04-16

**Authors:** Chanu Rhee, Sameer S. Kadri, John P. Dekker, Robert L. Danner, Huai-Chun Chen, David Fram, Fang Zhang, Rui Wang, Michael Klompas

**Affiliations:** 1 Department of Population Medicine, Harvard Pilgrim Health Care Institute, Harvard Medical School, Boston, Massachusetts; 2 Division of Infectious Diseases, Brigham and Women’s Hospital, Boston, Massachusetts; 3 Critical Care Medicine Department, Clinical Center, National Institutes of Health, Bethesda, Maryland; 4 Laboratory of Clinical Immunology and Microbiology, National Institutes of Allergy and Infectious Diseases, National Institutes of Health, Bethesda, Maryland; 5 Commonwealth Informatics, Waltham, Massachusetts

## Abstract

**Importance:**

Broad-spectrum antibiotics are recommended for all patients with suspected sepsis to minimize the risk of undertreatment. However, little is known regarding the net prevalence of antibiotic-resistant pathogens across all patients with community-onset sepsis or the outcomes associated with unnecessarily broad empiric treatment.

**Objective:**

To elucidate the epidemiology of antibiotic-resistant pathogens and the outcomes associated with both undertreatment and overtreatment in patients with culture-positive community-onset sepsis.

**Design, Setting, and Participants:**

This cohort study included 17 430 adults admitted to 104 US hospitals between January 2009 and December 2015 with sepsis and positive clinical cultures within 2 days of admission. Data analysis took place from January 2018 to December 2019.

**Exposures:**

Inadequate empiric antibiotic therapy (ie, ≥1 pathogen nonsusceptible to all antibiotics administered on the first or second day of treatment) and unnecessarily broad empiric therapy (ie, active against methicillin-resistant *Staphylococcus aureus* [MRSA]; vancomycin-resistant *Enterococcus* [VRE]; ceftriaxone-resistant gram-negative [CTX-RO] organisms, including *Pseudomonas aeruginosa*; or extended-spectrum β-lactamase [ESBL] gram-negative organisms when none of these were isolated).

**Main Outcomes and Measures:**

Prevalence and empiric treatment rates for antibiotic-resistant organisms and associations of inadequate and unnecessarily broad empiric therapy with in-hospital mortality were assessed, adjusting for baseline characteristics and severity of illness.

**Results:**

Of 17 430 patients with culture-positive community-onset sepsis (median [interquartile range] age, 69 [57-81] years; 9737 [55.9%] women), 2865 (16.4%) died in the hospital. The most common culture-positive sites were urine (9077 [52.1%]), blood (6968 [40.0%]), and the respiratory tract (2912 [16.7%]). The most common pathogens were *Escherichia coli* (5873 [33.7%]), *S aureus* (3706 [21.3%]), and *Streptococcus* species (2361 [13.5%]). Among 15 183 cases in which all antibiotic-pathogen susceptibility combinations could be calculated, most (12 398 [81.6%]) received adequate empiric antibiotics. Empiric therapy targeted resistant organisms in 11 683 of 17 430 cases (67.0%; primarily vancomycin and anti-*Pseudomonal* β-lactams), but resistant organisms were uncommon (MRSA, 2045 [11.7%]; CTX-RO, 2278 [13.1%]; VRE, 360 [2.1%]; ESBLs, 133 [0.8%]). The net prevalence for at least 1 resistant gram-positive organism (ie, MRSA or VRE) was 13.6% (2376 patients), and for at least 1 resistant gram-negative organism (ie, CTX-RO, ESBL, or CRE), it was 13.2% (2297 patients). Both inadequate and unnecessarily broad empiric antibiotics were associated with higher mortality after detailed risk adjustment (inadequate empiric antibiotics: odds ratio, 1.19; 95% CI, 1.03-1.37; *P* = .02; unnecessarily broad empiric antibiotics: odds ratio, 1.22; 95% CI, 1.06-1.40; *P* = .007).

**Conclusions and Relevance:**

In this study, most patients with community-onset sepsis did not have resistant pathogens, yet broad-spectrum antibiotics were frequently administered. Both inadequate and unnecessarily broad empiric antibiotics were associated with higher mortality. These findings underscore the need for better tests to rapidly identify patients with resistant pathogens and for more judicious use of broad-spectrum antibiotics for empiric sepsis treatment.

## Key Points

**Question:**

What is the prevalence of antibiotic resistance in community-onset sepsis, and is there risk associated with the receipt of empiric broad-spectrum antibiotics?

**Findings:**

In this cohort study of 17 430 adults with culture-positive sepsis admitted to 104 US hospitals, 67.0% received empiric broad-spectrum antibiotics, but resistant gram-positive organisms were isolated in only 13.6% of patients and resistant gram-negative organisms in 13.2%. Both undertreatment (failure to cover organisms) and overtreatment (resistant organisms targeted but not isolated) were associated with higher mortality after detailed risk adjustment.

**Meaning:**

In this study, broad-spectrum antibiotics were frequently administered to patients with community-onset sepsis without resistant organisms, and these therapies were associated with worse outcomes.

## Teaser

This cohort study elucidates the epidemiology of antibiotic-resistant pathogens and the outcomes associated with both undertreatment and overtreatment among patients with culture-positive community-onset sepsis.

## Introduction

Sepsis, the syndrome of life-threatening organ dysfunction complicating severe infection, is a leading cause of death in hospitalized patients.^1^ Early active antibiotic therapy is associated with better outcomes.^2,3,4,5^ Therefore, national quality measures and international guidelines recommend immediate empiric broad-spectrum antibiotics for all patients with suspected sepsis.^6,7^

However, it is unclear how many patients presenting with sepsis require coverage for methicillin-resistant *Staphylococcus aureus* (MRSA), *Pseudomonas aeruginosa*, and other potentially resistant pathogens. Most existing epidemiologic studies present rates of resistance to selected antimicrobials per pathogen rather than quantifying overall resistance rates across all patients and syndromes associated with sepsis. These data are needed to inform the rational use of antibiotics, given that overuse of broad-spectrum therapy may also confer harm by selecting for antibiotic-resistant bacteria, increasing the risk of adverse events, such as *Clostridioides difficile* infections, and raising costs.^8,9,10,11,12^ Overtreatment has also been associated with higher mortality rates in some populations.^13,14,15^ Therefore, we sought to elucidate the epidemiology of antibiotic-resistant pathogens in patients with culture-positive community-onset sepsis and the risks of both inadequate and unnecessarily broad antibiotic treatments in US hospitals.

## Methods

### Study Design, Data Source, and Population

We conducted a retrospective cohort study using Cerner HealthFacts, a deidentified data set that includes detailed electronic clinical data from diverse US hospitals.^16,17,18,19,20^ We included all patients aged at least 20 years who were admitted between January 2009 and September 2015, excluding those with missing discharge dispositions or *International Classification of Diseases, Tenth Revision, Clinical Modification* (*ICD*-*10*-*CM*) rather than *ICD*-*9*-*CM* codes.^16^ Data analysis took place between January 2018 and December 2019 and followed the Strengthening the Reporting of Observational Studies in Epidemiology (STROBE) reporting guideline for cohort studies.^21^ This study was approved by the institutional review board at Harvard Pilgrim Health Care Institute with a waiver of informed consent given that the patients in this study had high mortality rates, making collection of consent infeasible.

### Identifying Patients With Culture-Positive Sepsis

We identified sepsis hospitalizations using the US Centers for Disease Control and Prevention Adult Sepsis Event surveillance criteria, which defines sepsis as concurrent evidence of presumed serious infection (ie, blood culture order and ≥4 consecutive days of new antibiotics) and organ dysfunction (ie, initiation of vasopressors or mechanical ventilation, lactate level ≥2.0 mmol/L [to convert to milligram per deciliter, divide by 0.111], doubling in baseline creatinine level or ≥50% decrease in estimated glomerular filtration rate, doubling in total bilirubin level to ≥2.0 mg/dL [to convert to micromoles per liter, multiply by 17.104], or ≥50% decrease in platelet count to <100 × 10^3^/μL [to convert to × 10^9^/L, multiply by 1.0]).^22^ We focused on culture-positive community-onset sepsis, as defined by a blood culture draw, first antibiotic administration, organ dysfunction, and clinical cultures obtained and subsequently testing positive for potentially pathogenic organisms, all on hospital day 2 or earlier. We excluded culture-negative patients because of the difficulty determining antibiotic appropriateness in this population using electronic data alone. We further excluded patients transferred from hospitals, rehabilitation or long-term facilities, and hospice as well as patients with hospital-onset sepsis because the epidemiology, antibiotic resistance patterns, and recommended treatments differ substantially in health care–acquired vs community-acquired infections.^23,24,25^ Infectious syndromes were classified using *ICD*-*9*-*CM* discharge codes (eAppendix 1 in the Supplement).^26^

### Culture Sites and Pathogens of Interest

We included clinical cultures from the following anatomic sites: blood, respiratory, urine, deep tissue, central nervous system fluid, body fluid, and superficial tissue. We focused on pathogens commonly encountered in routine practice. Gram-negative organisms included *Acinetobacter* species, *Citrobacter* species, *Enterobacter* species, *Escherichia coli*, *Klebsiella* species, *Proteus* species, *P aeruginosa*, and *Serratia* species. Gram-positive organisms included *S aureus*, *Streptococcus* species, and *Enterococcus* species. Surveillance cultures and cultures positive for other organisms within the first 2 days of hospitalization were excluded, including coagulase-negative *Staphylococcus* species (because it can be difficult to distinguish contaminants vs true infections) and *Enterococcus* species isolated from respiratory samples.

We assessed the prevalence of the following resistant organisms: MRSA, vancomycin-resistant *Enterococcus* (VRE), extended spectrum β-lactamase (ESBL) gram-negative organisms (ie, resistant to all β-lactams except carbapenems), carbapenem-resistant *Enterobacteriaceae* (CRE; defined as resistant to imipenem, meropenem, doripenem, or ertapenem), and ceftriaxone-resistant gram negatives (CTX-RO; including *P aeruginosa* and ESBLs). We grouped *P aeruginosa* with other CTX-RO organisms because they share the need for treatment with β-lactams with anti-*Pseudomonal* coverage, which are an important class of broad-spectrum antibiotics.

### Antibiotic Susceptibilities and Adequacy of Therapy

We assessed each potential antibiotic-pathogen combination using antibiotic susceptibilities derived from in vitro reports generated by each institution. Intermediate susceptibilities were treated as nonsusceptible. In some cases, susceptibilities to specific antibiotics administered were not explicitly listed but could be assumed using knowledge of the spectrum of activity for each antibiotic-pathogen combination. For example, many antibiotics are intrinsically inactive against certain species (eg, ceftriaxone vs* P aeruginosa*) and are therefore not included in susceptibility reports. Alternatively, a gram-negative organism susceptible to ceftriaxone may not have susceptibilities reported to all higher-generation cephalosporins (ie, cefepime), but these agents can be safely used. We created rules to impute susceptibilities for each antibiotic-pathogen combination (eAppendix 2 in the Supplement).

We considered patients to have received inadequate empiric therapy if at least 1 pathogen isolated from any clinical culture site was not susceptible to all antibiotics administered on the first and second day of treatment. We considered patients to have received unnecessarily broad empiric therapy if they received adequate empiric therapy and anti-MRSA antibiotics (ie, vancomycin, linezolid, or daptomycin), anti-VRE antibiotics (ie, linezolid or daptomycin), anti-*Pseudomonal* β-lactams (ie, ceftazidime, cefepime, piperacillin-tazobactam, aztreonam, imipenem, meropenem, or doripenem), or carbapenems (ie, imipenem, meropenem, doripenem, or ertapenem), but none of the organisms targeted by these antibiotics (ie, MRSA, VRE, CTX-RO, or ESBL) were recovered. We did not consider CRE in the analysis of unnecessarily broad therapy given the infrequency of empiric CRE treatment.^27^

### Outcomes

We examined associations of both inadequate and unnecessarily broad empiric regimens with in-hospital mortality. The analysis of unnecessarily broad antibiotics was limited to patients who received adequate antibiotics because an empiric regimen would generally not be considered unnecessarily broad if the spectrum was inadequate. Secondary outcomes included hospital-onset acute kidney injury, defined as an increase in creatinine level by at least 0.5 mg/dL (to convert to micrograms per liter, multiply by 1) at any point during hospitalization relative to the initial value on presentation and *C difficile* infections, which we identified using the *ICD*-*9*-*CM* code 008.45 because *C difficile* test results were unavailable in our data set.

### Statistical Analysis

We fit logistic regression models using generalized estimating equations to account for clustering within hospitals. Model covariates included admission year, hospital characteristics (bed size, region, teaching status), patient demographic characteristics (age, sex, race), total burden of comorbidities (Agency for Healthcare Research and Quality [AHRQ] Elixhauser Comorbidity Index^28^), microbiologic characteristics (site of positive clinical culture, pathogen, and presence of antibiotic resistance [MRSA, VRE, CTX-RO, ESBL, or CRE]), infectious syndrome (by *ICD*-*9*-*CM *codes), intensive care unit (ICU) care on admission, and physiological markers of severity of illness on admission (number of vasopressors, need for mechanical ventilation, and worst values for temperature, systolic blood pressure, respiratory rate, Glasgow Coma Scale score, serum lactate level, creatinine level, anion gap, total bilirubin level, aspartate aminotransferase level, white blood cell count, hematocrit level, platelet count, and serum albumin level). We used univariate logistic regression to assess associations of covariates with each outcome. Covariates with univariate *P* < .10 were included in the multivariable models. The 2247 cases (12.9%) in which susceptibility or resistance to all administered antibiotics could not be reliably imputed were excluded from this multivariable analysis (eAppendix 3 in the Supplement).

Multiple imputation was used in our primary analysis to assign values for missing severity-of-illness physiological variables (eAppendix 3 in the Supplement). Several sensitivity analyses were conducted to handle missing data in different ways. First, we imputed all missing severity-of-illness covariates with median values. Second, we limited analyses to patients with nonmissing vital signs (ie, temperature, blood pressure, and respiratory rate) and used multiple imputation to account for other missing covariates. Third, we limited analyses to patients with complete data for all covariates.

For our assessment of inadequate antibiotic therapy and mortality, we conducted an additional sensitivity analysis restricted to positive blood cultures because these organisms unequivocally represent true pathogens, whereas organisms isolated from most other sites can sometimes be colonizers rather than pathogens. We also conducted subgroup analyses restricted to patients with septic shock, as defined by the need for vasopressors on admission.

All tests of significance used a 2-sided *P* < .05. Analyses were conducted using SAS version 9.4 (SAS Institute).

## Results

### Patient Characteristics and Pathogen Epidemiology

**Figure 1.**
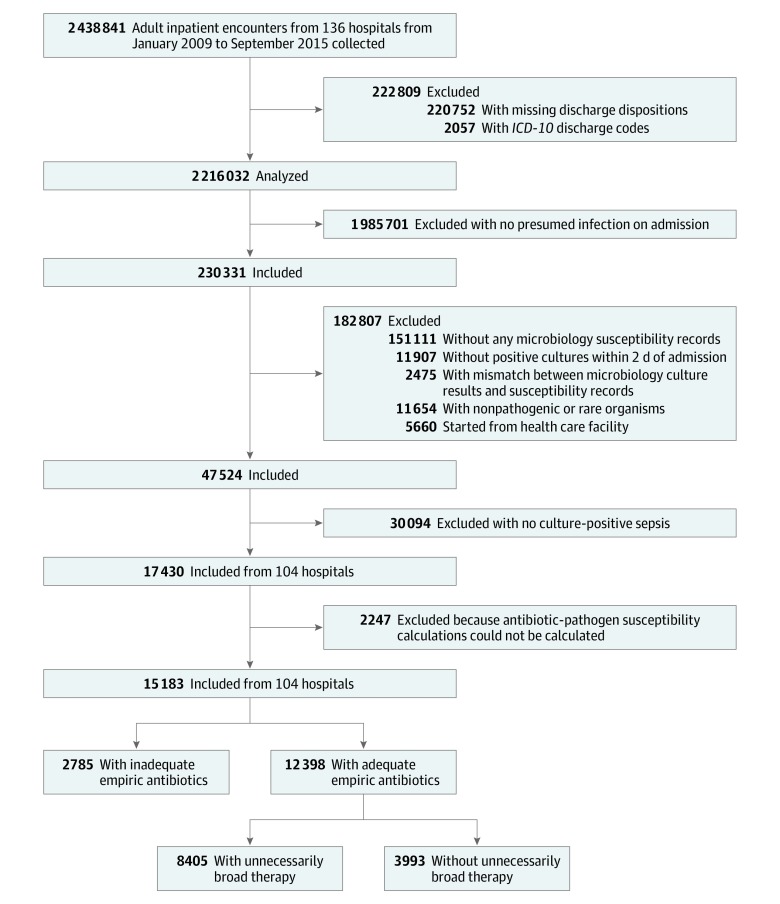
Study Cohort Flowchart *ICD-10* indicates *International Statistical Classification of Diseases and Related Health Problems, Tenth Revision*.

The cohort included 17 430 patients with culture-positive community-onset sepsis (median [interquartile range {IQR}] age, 69 [57-81] years; 9737 [55.9%] women) (Figure 1). Of these, 5609 (32.2%) had septic shock requiring vasopressors, 8001 (45.9%) were admitted to the ICU, and 2865 (16.4%) died in the hospital (Table 1). Patients with septic shock had higher mortality than those without shock (1733 of 5345 [32.4%] vs 1137 of 12 098 [9.4%]; *P* < .001).

**Table 1.** Characteristics of 17 430 Patients With Culture-Positive Sepsis

Characteristic	No. (%)			*P* value
	Culture-positive sepsis (N = 17 430)	Resistant pathogen isolated (n = 4474)^a^	No resistant pathogen isolated (n = 12 956)	
Age, median (IQR), y	69 (57-81)	68 (56-79)	69 (57-81)	<.001
Sex				
Men or unknown^b^	7692 (44.1)	2269 (50.7)	5424 (41.9)	<.001
Women	9737 (55.9)	2206 (49.3)	7531 (58.1)	
Race				
White	12 740 (73.1)	3105 (69.4)	9635 (74.4)	<.001
Black	3365 (19.3)	1052 (23.5)	2313 (17.9)	
Other or unknown^b^	1325 (7.6)	317 (7.1)	1008 (7.8)	
Select comorbidities^c^				
Cancer	1487 (8.5)	383 (8.6)	1104 (8.5)	.94
Chronic lung disease	3524 (20.2)	1124 (31.9)	2400 (18.5)	<.001
Congestive heart failure	3476 (19.9)	1048 (23.4)	2428 (18.7)	<.001
Diabetes	5402 (31.0)	1402 (31.3)	4000 (30.9)	.56
Liver disease	956 (5.5)	215 (4.8)	741 (5.7)	.02
Neurologic disease	3177 (18.2)	893 (20.0)	2284 (17.6)	<.001
Peripheral vascular disease	1271 (7.3)	387 (8.7)	884 (6.8)	<.001
Renal disease	3411 (19.6)	1013 (22.6)	2398 (18.5)	<.001
AHRQ Elixhauser Comorbidity Index score, median (IQR)^c^	11 (3-19)	12 (4-20)	11 (3-19)	<.001
Infectious syndrome^d^				
Pulmonary	5728 (32.9)	1851 (41.4)	3877 (29.9)	<.001
Urinary	8515 (48.9)	1867 (41.7)	6648 (51.3)	<.001
Intra-abdominal	2373 (13.6)	540 (12.1)	1833 (14.2)	<.001
Skin or soft tissue	1787 (10.3)	572 (12.8)	1215 (9.4)	<.001
Bone or joint	600 (3.4)	224 (5.0)	376 (2.9)	<.001
Central nervous system	179 (1.0)	36 (0.8)	143 (1.1)	.09
Obstetric or gynecologic	100 (0.6)	22 (0.5)	78 (0.6)	.40
Other	5130 (29.4)	1396 (31.2)	73 734 (28.8)	.003
Culture site				
Blood	6968 (40.0)	1590 (35.5)	5378 (41.5)	<.001
Body fluid	958 (5.5)	261 (5.8)	697 (5.4)	.25
Central nervous system	51 (0.3)	9 (0.2)	42 (0.3)	.19
Deep tissue	175 (1.0)	50 (1.1)	125 (1.0)	.38
Other	175 (1.0)	140 (3.1)	35 (0.3)	<.001
Respiratory	2912 (16.7)	1339 (29.9)	1573 (12.1)	<.001
Superficial	1674 (9.6)	836 (18.7)	838 (6.5)	<.001
Urine	9077 (52.1)	1868 (41.8)	7209 (55.6)	<.001
Sepsis organ dysfunction^e^				
Vasopressors	5609 (32.2)	1612 (36.0)	3997 (30.9)	<.001
Mechanical ventilation	3753 (21.5)	1264 (28.3)	2489 (19.2)	<.001
Renal	9176 (52.6)	2238 (50.0)	6938 (53.6)	<.001
Lactate	7543 (43.3)	1846 (41.3)	5697 (44.0)	.002
Hepatic	1889 (10.8)	323 (7.2)	1566 (12.1)	<.001
Platelets	2077 (11.9)	446 (10.0)	1631 (12.6)	<.001
SOFA score on admission, median (IQR)	4 (2-7)	4 (2-7)	4 (2-7)	<.001
Hospital LOS, median (IQR), d	8 (5-13)	9 (6-15)	8 (5-13)	<.001
Admitted to ICU	8001 (45.9)	2243 (50.1)	5758 (44.4)	<.001
ICU LOS, median (IQR), d	4 (3-7)	4 (3-7)	4 (3-7)	<.001
In-hospital death	2865 (16.4)	888 (19.9)	1977 (15.3)	<.001

Abbreviations: AHRQ, Agency for Healthcare Research and Quality; ICU, intensive care unit; IQR, interquartile range; LOS, length of stay; SOFA, Sequential Organ Failure Assessment.

^a^ Resistant pathogens include methicillin-resistant *Staphylococcus aureus*, ceftriaxone-resistant gram-negative organisms (including *Pseudomonas aeruginosa*), vancomycin-resistant *Enterococcus*, extended spectrum β-lactamase producing gram-negative organisms, and carbapenem-resistant *Enterobacteriaceae*.

^b^ Sex was missing for 1 patient; race was missing for 243 patients.

^c^ Comorbidities were calculated using the Elixhauser method as adapted by AHRQ. Cancer comorbidity included the Elixhauser categories of solid tumor without metastases, metastatic tumor, and lymphoma. Diabetes included diabetes with and without complications. The AHRQ Elixhauser Comorbidity Index score is weighted and allows for negative points for comorbidities with an inverse association with mortality.

^d^ Infectious syndromes were determined by *International Classification of Diseases, Ninth Revision, Clinical Modification* diagnosis codes on discharge.

^e^ Sepsis organ dysfunction was defined by Centers for Disease Control and Prevention Adult Sepsis Event criteria.

**Figure 2.**
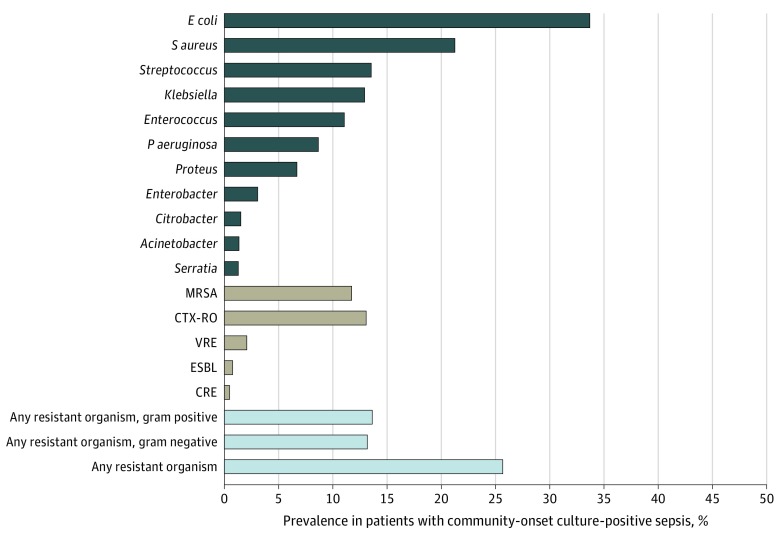
Prevalence of Pathogens in Culture-Positive Community-Onset Sepsis The reported prevalence of each pathogen is relative to 17 430 patients with culture-positive community-onset sepsis in the cohort from any clinical culture site. Only pathogens isolated within the first 2 days of hospitalization were analyzed. The same pathogen isolated from different sites from the same patient was counted as 1 pathogen. Of 2278 patients with ceftriaxone-resistant gram-negative organism (CTX-RO), 1510 (66.3%) had *Pseudomonas aeruginosa*. CRE indicates carbapenem-resistant *Enterobacteriaceae*; *E coli*, *Escherichia coli*; ESBL, extended-spectrum β-lactamase producing gram-negative organism; MRSA, methicillin-resistant *Staphylococcus aureus*; and VRE, vancomycin-resistant *Enterococcus*.

Urinary tract infection was the most common infectious diagnosis (8515 [48.9%]), followed by pulmonary (5728 [32.9%]), intra-abdominal (2373 [13.6%]), and skin or soft tissue (1787 [10.3%]) infections. The most common positive culture sites were urine (9077 [52.1%]), blood (6968 [40.0%]), and the respiratory tract (2912, [16.7%]). The top pathogens were *E coli* (5873 [33.7%]), *S aureus* (3706 [21.3%]), *Streptococcus* species (2361 [13.5%]), *Klebsiella* (2254 [12.9%]), and *Enterococcus* (1928 [11.1%]) (Figure 2). Empiric therapy targeted resistant organisms in 11 683 of 17 430 cases (67.0%). Drug-resistant pathogens were relatively uncommon (MRSA, 2045 [11.7%]; CTX-RO, 2278 [13.1%], of whom 1510 patients [66.3%] had *P aeruginosa*; VRE, 360 [2.1%]; ESBLs, 133 [0.8%]; and CRE, 83 [0.5%]). The net prevalence of at least 1 resistant gram-positive organism (ie MRSA or VRE) was 13.6% (2376 patients); at least 1 resistant gram negative organism (ie, CTX-RO, ESBL, CRE), 13.2% (2297 patients); and any of these organisms, 25.7% (4474 patients). The prevalence of pathogens across blood, respiratory, and urine cultures is shown in eFigure 1 in the Supplement.

The characteristics of patients with vs without resistant organisms (4474 [25.7%] vs 12 956 [74.3%]) are shown in Table 1. Patients with resistant organisms were more likely to have more comorbidities (median [IQR] AHRQ Elixhauser Comorbidity Index score, 12 [4-20] vs 11 [3-19]; *P* < .001), to have a pulmonary infection (1851 [41.4%] vs 3877 [29.9%]; *P* < .001), to have positive respiratory cultures (1339 [29.9%] vs 1573 [12.1%]; *P* < .001), to require vasopressors (1612 [36.0%] vs 3997 [30.9%]; *P* < .001) or mechanical ventilation (1264 [28.3%] vs 2489 [19.2%]; *P* < .001), to require ICU admission (2243 [50.1%] vs 5758 [44.4%]; *P* < .001), and to die in the hospital (888 [19.9%] vs 1977 [15.3%]; crude odds ratio [OR], 1.38; 95% CI, 1.26-1.50).

The prevalence of resistant organisms was higher in the 5609 patients with septic shock vs 11 821 patients without shock (1612 [28.7%] vs 2862 [24.2%]; *P* < .001), including higher rates of MRSA (785 [14.0%] vs 1260 [10.7%]; *P* < .001) and CTX-RO (801 [14.3%] vs 1477 [12.5%]; *P* = .001) but not ESBL (49 [0.9%] vs 84 [0.7%]; *P* = .25), VRE (117 [2.1%] vs 243 [2.1%]; *P* = .90), or CRE (52 [0.9%] vs 31 [0.3%]; *P* = .31) (eFigure 2 in the Supplement).

### Empiric Antibiotics and Rates of Overtreatment for Resistant Pathogens

**Figure 3.**
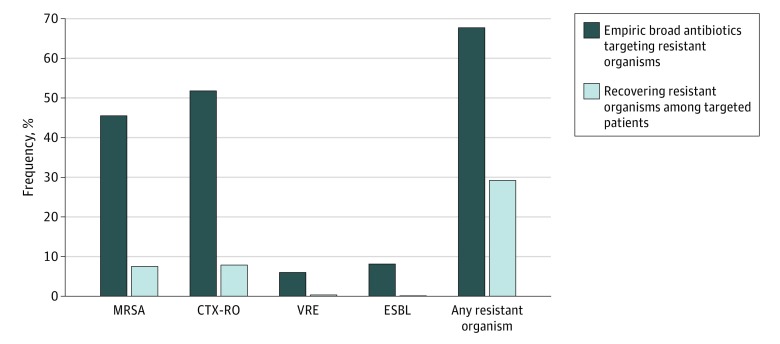
Proportion of Culture-Positive Sepsis Patients Treated With Broad-Spectrum Antibiotics in Whom Targeted Resistant Organisms Were Subsequently Recovered The dark bars indicate the proportion of 17 430 patients with culture-positive sepsis on admission who received empiric antibiotics directed at specific resistant organisms. Anti–methicillin-resistant *Staphylococcus aureus *(MRSA) antibiotics include vancomycin, linezolid, and daptomycin; anti–ceftriaxone-resistant gram-negative organism (CTX-RO) antibiotics (ie, anti-*Pseudomonal* β-lactams) include ceftazidime, cefepime, piperacillin-tazobactam, aztreonam, imipenem, meropenem, and doripenem; anti–vancomycin-resistant *Enterococcus* (VRE) antibiotics include linezolid or daptomycin; and anti–extended-spectrum β-lactamase (ESBL) producing gram-negative organism antibiotics include carbapenems (ie, imipenem, meropenem, doripenem, or ertapenem). The light bars indicate the proportion of patients treated with antibiotics directed at resistant organisms who had that organism recovered from any clinical site within the first 2 days of hospitalization.

Vancomycin was the most commonly prescribed empiric antibiotic (7262 [41.7%]), followed by an anti-*Pseudomonal* fluoroquinolone (ciprofloxacin or levofloxacin, 6997 [40.1%]), piperacillin-tazobactam (5911 [33.9%]), ceftriaxone (5187 [29.8%]), and third- or fourth-generation cephalosporins (ceftazidime or cefepime, 2300 [13.2%]) (eFigure 3 in the Supplement). Anti-MRSA treatment (ie, vancomycin, linezolid, or daptomycin) was given to 7936 of 17 430 patients (45.5%), of whom 1310 (16.5%) had positive cultures for MRSA. Anti-*Pseudomonal* β-lactams were given to 9031 patients (51.8%), of whom 1367 (15.1%) had positive cultures for CTX-ROs. Anti-VRE treatment was given to 1040 patients (6.0%), of whom 58 (5.6%) had positive cultures for VRE. Carbapenems were given to 1408 patients (8.1%), of whom 19 (1.4%) had positive cultures for ESBL. Overall, 11 797 patients (67.7%) received anti-MRSA, anti-*Pseudomonal*, anti-VRE, and/or anti-ESBL treatment, of whom 3447 (29.2%) had at least 1 of these organisms isolated (Figure 3). In addition, 4090 patients (23.5%) received double coverage for gram-negative organisms; 3260 patients (8.7%) received anti-*Pseudomonal* β-lactams with ciprofloxacin or levofloxacin, and 830 patients (4.8%) received anti-*Pseudomonal* β-lactams with amikacin, gentamicin, or tobramycin.

### Associations Between Empiric Therapy, Antibiotic Resistance, and Outcomes

The crude and adjusted associations between empiric therapy patterns and outcomes are summarized in Table 2. Empiric antibiotics were active against all isolated pathogens in 12 398 of 15 183 (81.6%) sepsis cases in which all antibiotic-pathogen susceptibility combinations could be calculated. Compared with 12 398 patients who received adequate therapy, 2785 patients who received inadequate empiric antibiotic therapy were older (median [IQR] age, 71 [60-83] years vs 68 [56-80] years; *P* < .001) and had a higher burden of comorbidities (median [IQR] AHRQ Elixhauser Comorbidity Index score, 13 [5-21] vs 11 [2-19]; *P* < .001), but the groups had similar rates of organ dysfunction (eg, renal dysfunction: 1480 [53.1%] vs 6465 [52.2%]; *P* = .34), ICU admission (1248 [44.8%] vs 5710 [46.1%]; *P* = .23), and in-hospital mortality (488 [17.5%] vs 2011 [16.3%]; *P* = .09) (eTable 1 in the Supplement). However, on multivariable analysis, inadequate therapy was significantly associated with higher mortality (adjusted OR, 1.19; 95% CI, 1.03-1.37; *P* = .02).

**Table 2.** Outcomes Associated With Inadequate and Unnecessarily Broad Empiric Antibiotic Therapy^a^

Outcome	Inadequate vs adequate empiric therapy						Unnecessarily broad vs not unnecessarily broad empiric therapy^b^					
	No./total No. (%)		Unadjusted OR (95% CI)	*P *value	Adjusted OR (95% CI)	*P *value	No./total No. (%)		Unadjusted OR (95% CI)	*P *value	Adjusted OR (95% CI)	*P *value
	Inadequate	Adequate empiric therapy					Unnecessarily broad	Not unnecessarily broad				
In-hospital death	488/2785 (17.5)	2011/12 388 (16.3)	1.10 (0.98-1.22)	.09	1.19 (1.03-1.37)	.02	1575/8405 (18.7)	436/3993 (10.9)	1.88 (1.68-2.11)	<.001	1.22 (1.06-1.40)	.007
Hospital-onset acute kidney injury	486/2785 (17.5)	2196/12 398 (17.7)	0.98 (0.88-1.09)	.74	1.02 (0.90-1.16)	.72	1641/8405 (19.5)	555/3993 (13.9)	1.50 (1.35-1.67)	<.001	1.12 (1.00-1.26)	.05
*Clostridioides difficile*	207/2785 (7.4)	498/12 398 (4.0)	1.92 (1.63-2.27)	<.001	1.19 (0.98-1.45)	.09	367/8405 (4.4)	131/3993 (3.3)	1.34 (1.10-1.65)	.004	1.26 (1.01-1.57)	.04

Abbreviation: OR, odds ratio.

^a^ Each model was adjusted for admission year, hospital characteristics, patient demographic characteristics (age, sex, race), comorbidities, microbiologic characteristics (site of positive culture, pathogen, presence of antibiotic resistance), infectious syndrome, care in intensive care unit on admission, vasopressors, mechanical ventilation, vital signs, Glasgow Coma Scale score, and laboratory values.

^b^ The analysis of unnecessarily broad vs not unnecessarily broad empiric therapy was conducted among patients who received adequate therapy.

Inadequate therapy was much more likely in patients with resistant pathogens (MRSA, VRE, CTX-RO, ESBL, or CRE) vs nonresistant pathogens (1544 of 3811 [40.5%] vs 1241 of 11 372 [10.9%]; *P* < .001). Although patients with antibiotic-resistant organisms had higher crude hospital mortality rates, there was no difference after adjusting for baseline characteristics, severity of illness, and adequacy of therapy (adjusted OR, 1.04; 95% CI, 0.83-1.30; *P* = .75). There was also no association between antibiotic-resistant organisms and mortality when only considering positive blood cultures (adjusted OR, 1.10; 95% CI, 0.82-1.46; *P* = .54).

Inadequate antibiotic therapy was not significantly associated with hospital death in the subgroup of patients with septic shock (OR, 1.10; 95% CI, 0.87-1.38; *P* = .44), but there was an association between inadequate antibiotics and hospital death when considering patients with sepsis who had positive blood cultures alone (adjusted OR, 1.40; 95% CI, 1.07-1.84; *P* = .02). The risk of *C difficile* was similar in patients who received inadequate vs adequate therapy (adjusted OR 1.19; 95% CI, 0.98-1.45; *P* = .09) as was the risk of hospital-onset acute kidney injury (adjusted OR, 1.02; 95% CI, 0.90-1.16; *P* = .72).

Patients who received adequate but unnecessarily broad empiric antibiotics were younger and had a similar burden of comorbidities compared with those who did not receive unnecessarily broad therapy (median [IQR] age, 67 [55-79] years vs 71 [58-82] years; *P* < .001; median [IQR] AHRQ Elixhauser Comorbidity Index score, 11 [2-19] vs 11 [3-18]; *P* = .11) but were more severely ill on admission, with higher rates of vasopressor use (3310 [39.4%] vs 858 [21.5%]; *P* < .001), mechanical ventilation (1987 [23.6%] vs 601 [15.1%]; *P* < .001), ICU care (4276 [50.9%] vs 1434 [35.9%]; *P* < .001), and crude mortality (1575 [18.7%] vs 436 [10.9%]; *P* < .001) (eTable 2 in the Supplement). The association between unnecessarily broad empiric antibiotics and higher mortality persisted after risk adjustment (adjusted OR, 1.22; 95% CI, 1.06-1.49; *P* = .007). On subgroup analysis, the association between unnecessarily broad therapy and higher mortality was only seen in patients with sepsis and without shock (adjusted OR, 1.33; 95% CI, 1.09-1.60; *P* = .005) but not in patients with septic shock (adjusted OR, 1.12; 95% CI, 0.90-1.40; *P* = .32). The risk of *C difficile* among patients with sepsis was higher with unnecessarily broad therapy (adjusted OR, 1.26; 95% CI, 1.01-1.57; *P* = .04), but there was no association between unnecessarily broad therapy and hospital-onset acute kidney injury (adjusted OR, 1.12; 95% CI, 1.00-1.26; *P* = .05). The median (IQR) duration of treatment in the unnecessarily broad group was 3 (1-5) days for vancomycin, 4 (2-6) days for anti-*Pseudomonal* β-lactams, and 4 (2-6) days for carbapenems.

The full univariate and multivariable models for mortality are shown in eTable 3 in the Supplement. The distribution of severity-of-illness covariates is shown in eTable 4 in the Supplement. The frequencies of missing data within the first 2 days of hospitalization are shown in eFigure 4 in the Supplement and were highest among laboratory data for lactate levels (7123 of 17 430 [40.9%]) but low for general chemistry (140 [0.8%]) and complete blood cell count variables (376 [2.2%]); vital sign data was missing in as many of 8309 cases (47.8%), and Glasgow Coma Scale scores were missing in 8872 cases (50.9%). Sensitivity analyses handling missing data in different ways, including limiting to patients with nonmissing data, yielded similar point estimates as the primary analysis (eTable 5 in the Supplement).

## Discussion

Prior studies have estimated the national and global burden of antimicrobial resistance,^29,30^ but our study is among the first to estimate the net prevalence of antibiotic resistance across all culture sites in patients with culture-positive community-onset sepsis. We found that approximately 1 in 8 patients had resistant gram-positive organisms (primarily MRSA and rarely VRE) and 1 in 8 had resistant gram-negative organisms (primarily ceftriaxone-resistant gram-negative organisms and rarely ESBL or CRE). More than two-thirds of patients received broad-spectrum therapy directed at resistant organisms, but MRSA was only isolated in 1 in 6 patients treated with vancomycin or linezolid, *P aeruginosa* or other ceftriaxone-resistant gram-negative organisms in 1 in 6 patients treated with anti-*Pseudomonal* agents, VRE in 1 in 16 patients treated with linezolid or daptomycin, and ESBLs in 1 in 70 patients treated with carbapenems. Both inadequate and unnecessarily broad empiric therapy were associated with higher mortality after detailed risk adjustment.

Our findings almost certainly overestimate the prevalence of resistant pathogens across the entire spectrum of patients treated for possible sepsis given the following: (1) we limited our analysis to bacterial, culture-positive sepsis, and between 30% and 50% of all patients with sepsis are culture negative^31,32^; (2) viruses are often implicated in severe pneumonia (the most common cause of sepsis), and (3) there are many noninfectious mimickers of sepsis that are treated as sepsis.^33,34^ All told, the net fraction of patients with sepsis who would benefit from broad-spectrum therapy, including agents active against both MRSA and *Pseudomonas*, is small. This may be an acceptable trade-off given the increased risk of death associated with inadequate therapy, but it underscores the need for rapid tests to more efficiently identify the small fraction of patients who truly need broad spectrum therapy.^35^ Alternatively, predictive models may soon allow clinicians to effectively select and tailor antibiotic regimens at the point of care.^36,37,38,39^

In our cohort, inadequate therapy was associated with a 20% to 40% higher odds of death depending on whether all cultures or only blood cultures were analyzed. Our estimates are in the range of the results of a meta-analysis of 48 studies that reported a pooled odds ratio of 1.6 for death associated with inadequate therapy in patients with sepsis.^40^ We also found that patients with antibiotic-resistant pathogens received inadequate empiric therapy 4 times as often compared with patients with nonresistant organisms; patients with resistant pathogens who received inadequate therapy had higher mortality rates. However, we did not find an association between antibiotic-resistant organisms and mortality after adjusting for baseline and clinical characteristics as well as adequacy of empiric antibiotics. This suggests that the higher crude mortality rates in patients with resistant organisms could be mediated by their higher comorbidity burden, greater severity of illness, and inadequate antibiotic therapy rather than intrinsic virulence of resistant organisms.^41,42,43^

While clinicians, guidelines, and policies understandably emphasize broad-spectrum antibiotics to ensure adequate empiric treatment,^44^ our findings suggest that the risk of inadequate therapy needs to be weighed against the risks of unnecessarily broad empiric antibiotics. Among patients who received adequate therapy, overtreatment was associated with a 20% increase in the odds of death. Other studies have also reported that more aggressive antibiotic regimens may be associated with higher mortality rates in critically ill patients.^13,14,15^ In our cohort, we found an association between overtreatment and mortality only among patients without shock. This may be because the morbidity of acute severe illness in patients with septic shock outweighs the possible morbidity of excessively broad antibiotic therapy, whereas in less critically ill patients, the morbidity of excessively broad antibiotics may be more significant. Another possibility is residual confounding among patients without shock because of the wide array of infections and organ dysfunction in this group that may make it more difficult to adequately adjust for all gradations in illness. The possibility of residual confounding is supported by the observation that more severely ill patients were more likely to receive broad-spectrum antibiotics.

There are several other potential explanations for the association between overtreatment and higher mortality. As many as 20% of hospitalized patients who receive antibiotics experience adverse effects.^45^ Even a single dose of antibiotics can increase the risk of *C difficile*; this risk is higher with broad- vs narrow-spectrum antibiotics.^46,47^ We found unnecessarily broad empiric therapy was associated with a 26% increased risk of *C difficile* infection in our study. Unnecessary antibiotics may also increase the risk of acute kidney injury.^48^ We observed a trend toward more acute kidney injury in patients treated with unnecessarily broad antibiotics. In particular, the combination of vancomycin and piperacillin-tazobactam, a common regimen during the study period, is associated with renal toxicity.^49^ Broad-spectrum antibiotics also disrupt the gut microbiome, an increasingly recognized modulator of the immune system and outcomes in sepsis.^50,51^ Lastly, broad-spectrum antibiotics may increase the risk of resistant hospital-acquired infections.

### Limitations

Our study has important limitations. First, we used a convenience sample of hospitals, which may limit generalizability. Antibiotic resistance rates vary substantially by region, hospital, and even within a facility. Second, our data did not allow us to calculate the time to antibiotics on the scale of hours, an important predictor of patient outcomes in some studies.^2,4^ Third, our primary analysis included pathogens isolated from all clinical cultures, but not all of these may be pathogenic. However, an analysis using only organisms isolated from blood demonstrated similar results with respect to the association between inadequate antibiotics and mortality. Fourth, we excluded patients with atypical pathogens owing to the complexity of determining adequate vs excessive treatment in this population. Fifth, *C difficile* assay results were unavailable in our data sets, so we had to use *ICD*-*9*-*CM* codes for this outcome; this prevented us from knowing whether *C difficile* infections developed while in the hospital or were present on admission and to what degree there was misclassification of true infections vs colonization.^52^ Sixth, we excluded patients transferred from other hospitals or health care facilities to focus on community-onset infections, but our data did not allow us to identify patients who might have been recently hospitalized. Seventh, our data sets did not allow us to examine the full array of potential complications of antibiotics, such as hepatitis, cytopenias, and drug eruptions. Data on patients’ allergies were also unavailable to us; therefore, we could not account for broad-spectrum antibiotics administered because patients were allergic to narrower agents (such as vancomycin or carbapenems for patients with β-lactam allergies). Furthermore, we only included patients with culture-positive sepsis, but a substantial fraction of patients with sepsis are culture negative.^31,32^ Determining the consequences of unnecessarily broad antibiotic therapy in the culture-negative population is an important topic for future research.

## Conclusions

In this study of a large US cohort, we found that most patients with culture-positive community-onset sepsis did not have resistant organisms; however, empiric, broad-spectrum antibiotics targeting these organisms were frequently prescribed. Both inadequate and unnecessarily broad empiric therapy were associated with higher mortality. These findings underscore the need for better diagnostic tests to rapidly identify resistant pathogens and an increased focus on judicious use of broad-spectrum antibiotics for the empiric treatment of sepsis.

## Notes

Supplement. **eAppendix 1.** List of *International Classification of Diseases, Ninth Revision, Clinical Modification* (*ICD*-*9*-*CM*) Codes Used to Determine Presumed Infectious Syndrome **eAppendix 2.** Imputations for Missing Antibiotic Susceptibilities by Pathogen **eAppendix 3**. Multiple Imputation Methods for Missing Data in Severity-of-Illness Covariates **eFigure 1.** Prevalence of Pathogens by Culture Site (Blood, Urine, Respiratory) for Patients With Community-Onset Sepsis **eFigure 2. **Prevalence of Resistant Organisms in Septic Shock vs Sepsis Without Shock **eFigure 3. **Frequency of Empiric Antibiotic Choices in Culture-Positive Community-Onset Sepsis **eFigure 4.** Quantity of Missing Data for Severity-of-Illness Covariates **eTable 1. **Characteristics of Sepsis Patients Who Received Adequate vs Inadequate Empiric Therapy **eTable 2. **Characteristics of Sepsis Patients Who Received Unnecessarily Broad vs Not Unnecessarily Broad Empiric Therapy **eTable 3. **Univariate and Multivariable Models Assessing Associations Between Inadequate or Unnecessarily Broad Empiric Antibiotic Therapy and In-Hospital Death **eTable 4. **Distribution of Values for Severity-of-Illness Covariates in Culture-Positive Sepsis Patients **eTable 5. **Sensitivity Analyses for Multivariable Models for In-Hospital Death Using Different Strategies to Account for Missing Severity-of-Illness Covariates
