# Supplementary material for: Prevalence of Antibiotic-Resistant Pathogens in Culture-Proven Sepsis and Outcomes Associated With Inadequate and Broad-Spectrum Empiric Antibiotic Use
Source: JAMA Netw Open. 2020 Apr 16;3(4):e202899. doi: 10.1001/jamanetworkopen.2020.2899 (PMC7163409; doi:10.1001/jamanetworkopen.2020.2899)
Supplement: Supplement. — eAppendix 1. List of International Classification of Diseases, Ninth Revision, Clinical Modification (ICD-9-CM) Codes Used to Determine Presumed Infectious Syndrome eAppendix 2. Imputations for Missing Antibiotic Susceptibilities by Pathogen eAppendix 3. Multiple Imputation Methods for Missing Data in Severity-of-Illness Covariates eFigure 1. Prevalence of Pathogens by Culture Site (Blood, Urine, Respiratory) for Patients With Community-Onset Sepsis eFigure 2. Prevalence of Resistant Organisms in Septic Shock vs Sepsis Without Shock eFigure 3. Frequency of Empiric Antibiotic Choices in Culture-Positive Community-Onset Sepsis eFigure 4. Quantity of Missing Data for Severity-of-Illness Covariates eTable 1. Characteristics of Sepsis Patients Who Received Adequate vs Inadequate Empiric Therapy eTable 2. Characteristics of Sepsis Patients Who Received Unnecessarily Broad vs Not Unnecessarily Broad Empiric Therapy eTable 3. Univariate and Multivariable Models Assessing Associations Between Inadequate or Unnecessarily Broad Empiric Antibiotic Therapy and In-Hospital Death eTable 4. Distribution of Values for Severity-of-Illness Covariates in Culture-Positive Sepsis Patients eTable 5. Sensitivity Analyses for Multivariable Models for In-Hospital Death Using Different Strategies to Account for Missing Severity-of-Illness Covariates [file jamanetwopen-3-e202899-s001.pdf]

Supplementary Online Content

Rhee C, Kadri SS, Dekker JP, et al. Prevalence of antibiotic-resistant pathogens in culture-proven sepsis and outcomes associated with inadequate and broad-spectrum empiric antibiotic use. *JAMA Netw Open*. 2020;3(4):e202899. doi:10.1001/jamanetworkopen.2020.2899

eAppendix 1. List of *International Classification of Diseases, Ninth Revision, Clinical Modification* (*ICD-9-CM*) Codes Used to Determine Presumed Infectious Syndrome

eAppendix 2. Imputations for Missing Antibiotic Susceptibilities by Pathogen

eAppendix 3. Multiple Imputation Methods for Missing Data in Severity-of-Illness Covariates

eFigure 1. Prevalence of Pathogens by Culture Site (Blood, Urine, Respiratory) for Patients With Community-Onset Sepsis

eFigure 2. Prevalence of Resistant Organisms in Septic Shock vs Sepsis Without Shock

eFigure 3. Frequency of Empiric Antibiotic Choices in Culture-Positive Community-Onset Sepsis

eFigure 4. Quantity of Missing Data for Severity-of-Illness Covariates

eTable 1. Characteristics of Sepsis Patients Who Received Adequate vs Inadequate Empiric Therapy

eTable 2. Characteristics of Sepsis Patients Who Received Unnecessarily Broad vs Not Unnecessarily Broad Empiric Therapy

eTable 3. Univariate and Multivariable Models Assessing Associations Between Inadequate or Unnecessarily Broad Empiric Antibiotic Therapy and In-Hospital Death

eTable 4. Distribution of Values for Severity-of-Illness Covariates in Culture-Positive Sepsis Patients

eTable 5. Sensitivity Analyses for Multivariable Models for In-Hospital Death Using Different Strategies to Account for Missing Severity-of-Illness Covariates

This supplementary material has been provided by the authors to give readers additional information about their work.

© 2020 Rhee C et al. *JAMA Network Open.*

eAppendix 1. List of *International Classification of Diseases, Ninth Revision, Clinical Modification* (*ICD-9-CM*) Codes Used to Determine Presumed Site of Infection

A. Bone/Joint

ICD-9-CM CODE	DESCRIPTION
'0955 '	SYPHILIS OF BONE
'09850'	GONOCOCCAL ARTHRITIS
'09853'	GONOCOCCAL SPONDYLITIS
'09859'	GC INFECT JOINT NEC
'00323'	SALMONELLA ARTHRITIS
'00324'	SALMONELLA OSTEOMYELITIS
'0261 '	STREPTOBACILLARY FEVER
'03682'	MENINGOCOCC ARTHROPATHY
'05671'	ARTHRITIS DUE TO RUBELLA
‘7300’	ACUTE OSTEOMYELITIS
'71100'	PYOGEN ARTHRITIS-UNSPEC
'71101'	PYOGEN ARTHRITIS-SHLDER
'71102'	PYOGEN ARTHRITIS-UP/ARM
'71103'	PYOGEN ARTHRITIS-FOREARM
'71104'	PYOGEN ARTHRITIS-HAND
'71105'	PYOGEN ARTHRITIS-PELVIS
'71106'	PYOGEN ARTHRITIS-L/LEG
'71107'	PYOGEN ARTHRITIS-ANKLE
'71108'	PYOGEN ARTHRITIS NEC
'71109'	PYOGEN ARTHRITIS-MULT
'71160'	MYCOTIC ARTHRITIS-UNSPEC
'71161'	MYCOTIC ARTHRITIS-SHLDER
'71162'	MYCOTIC ARTHRITIS-UP/ARM
'71163'	MYCOTIC ARTHRIT-FOREARM
'71164'	MYCOTIC ARTHRITIS-HAND
'71165'	MYCOTIC ARTHRITIS-PELVIS
'71166'	MYCOTIC ARTHRITIS-L/LEG
'71167'	MYCOTIC ARTHRITIS-ANKLE
'71168'	MYCOTIC ARTHRITIS NEC
'71169'	MYCOTIC ARTHRITIS-MULT
'71170'	HELMINTH ARTHRIT-UNSPEC
'71171'	HELMINTH ARTHRIT-SHLDER
'71172'	HELMINTH ARTHRIT-UP/ARM
'71173'	HELMINTH ARTHRIT-FOREARM
'71174'	HELMINTH ARTHRIT-HAND
'71175'	HELMINTH ARTHRIT-PELVIS
'71176'	HELMINTH ARTHRIT-L/LEG
'71177'	HELMINTH ARTHRIT-ANKLE

© 2020 Rhee C et al. *JAMA Network Open.*

'71178'	HELMINTH ARTHRIT NEC
'71179'	HELMINTH ARTHRIT-MULT
'71180'	INF ARTHRITIS NEC-UNSPEC
'71181'	INF ARTHRITIS NEC-SHLDER
'71182'	INF ARTHRITIS NEC-UP/ARM
'71183'	INF ARTHRIT NEC-FOREARM
'71184'	INF ARTHRITIS NEC-HAND
'71185'	INF ARTHRITIS NEC-PELVIS
'71186'	INF ARTHRITIS NEC-L/LEG
'71187'	INF ARTHRITIS NEC-ANKLE
'71188'	INF ARTHRIT NEC-OTH SITE
'71189'	INF ARTHRITIS NEC-MULT
'71190'	INF ARTHRITIS NOS-UNSPEC
'71191'	INF ARTHRITIS NOS-SHLDER
'71192'	INF ARTHRITIS NOS-UP/ARM
'71193'	INF ARTHRIT NOS-FOREARM
'71194'	INF ARTHRIT NOS-HAND
'71195'	INF ARTHRIT NOS-PELVIS
'71196'	INF ARTHRIT NOS-L/LEG
'71197'	INF ARTHRIT NOS-ANKLE
'71198'	INF ARTHRIT NOS-OTH SITE
'71199'	INF ARTHRITIS NOS-MULT
'73000'	AC OSTEOMYELITIS-UNSPEC
'73001'	AC OSTEOMYELITIS-SHLDER
'73002'	AC OSTEOMYELITIS-UP/ARM
'73003'	AC OSTEOMYELITIS-FOREARM
'73004'	AC OSTEOMYELITIS-HAND
'73005'	AC OSTEOMYELITIS-PELVIS
'73006'	AC OSTEOMYELITIS-L/LEG
'73007'	AC OSTEOMYELITIS-ANKLE
'73008'	AC OSTEOMYELITIS NEC
'73009'	AC OSTEOMYELITIS-MULT
'73010'	CHR OSTEOMYELITIS-UNSP
'73011'	CHR OSTEOMYELIT-SHLDER
'73012'	CHR OSTEOMYELIT-UP/ARM
'73013'	CHR OSTEOMYELIT-FOREARM
'73014'	CHR OSTEOMYELIT-HAND
'73015'	CHR OSTEOMYELIT-PELVIS
'73016'	CHR OSTEOMYELIT-L/LEG
'73017'	CHR OSTEOMYELIT-ANKLE
'73018'	CHR OSTEOMYELIT NEC
'73019'	CHR OSTEOMYELIT-MULT
'73020'	OSTEOMYELITIS NOS-UNSPEC

© 2020 Rhee C et al. *JAMA Network Open.*

'73021'	OSTEOMYELITIS NOS-SHLDER
'73022'	OSTEOMYELITIS NOS-UP/ARM
'73023'	OSTEOMYELIT NOS-FOREARM
'73024'	OSTEOMYELITIS NOS-HAND
'73025'	OSTEOMYELITIS NOS-PELVIS
'73026'	OSTEOMYELITIS NOS-L/LEG
'73027'	OSTEOMYELITIS NOS-ANKLE
'73028'	OSTEOMYELIT NOS-OTH SITE
'73029'	OSTEOMYELITIS NOS-MULT
'73030'	PERIOSTITIS-UNSPEC
'73031'	PERIOSTITIS-SHLDER
'73032'	PERIOSTITIS-UP/ARM
'73033'	PERIOSTITIS-FOREARM
'73034'	PERIOSTITIS-HAND
'73035'	PERIOSTITIS-PELVIS
'73036'	PERIOSTITIS-L/LEG
'73037'	PERIOSTITIS-ANKLE
'73038'	PERIOSTITIS NEC
'73039'	PERIOSTITIS-MULT
'73080'	BONE INFECT NEC-UNSPEC
'73081'	BONE INFECT NEC-SHLDER
'73082'	BONE INFECT NEC-UP/ARM
'73083'	BONE INFECT NEC-FOREARM
'73084'	BONE INFECT NEC-HAND
'73085'	BONE INFECT NEC-PELVIS
'73086'	BONE INFECT NEC-L/LEG
'73087'	BONE INFECT NEC-ANKLE
'73088'	BONE INFECT NEC-OTH SITE
'73089'	BONE INFECT NEC-MULT
'73090'	BONE INFEC NOS-UNSP SITE
'73091'	BONE INFECT NOS-SHLDER
'73092'	BONE INFECT NOS-UP/ARM
'73093'	BONE INFECT NOS-FOREARM
'73094'	BONE INFECT NOS-HAND
'73095'	BONE INFECT NOS-PELVIS
'73096'	BONE INFECT NOS-L/LEG
'73097'	BONE INFECT NOS-ANKLE
'73098'	BONE INFECT NOS-OTH SITE
'73099'	BONE INFECT NOS-MULT
'99666'	REACT-INTER JOINT PROST (Begin 1989)
'99667'	REACT-OTH INT ORTHO DEV (Begin 1989)

© 2020 Rhee C et al. *JAMA Network Open.*

B. Central Nervous System

ICD-9-CM CODE	DESCRIPTION
'09181'	ACUTE SYPHIL MENINGITIS
'0941 '	GENERAL PARESIS
'09481'	SYPHILITIC ENCEPHALITIS
'09489'	NEUROSYPHILIS NEC
'0949 '	NEUROSYPHILIS NOS
'09882'	GONOCOCCAL MENINGITIS
'00321'	SALMONELLA MENINGITIS
'0360 '	MENINGOCOCCAL MENINGITIS
'0470 '	COXSACKIE VIRUS MENING
'0471 '	ECHO VIRUS MENINGITIS
'0478 '	VIRAL MENINGITIS NEC
'0479 '	VIRAL MENINGITIS NOS
'0490 '	LYMPHOCYTIC CHORIOMENING
'0491 '	ADENOVIRAL MENINGITIS
'0530 '	HERPES ZOSTER MENINGITIS
'05472'	H SIMPLEX MENINGITIS
'0721 '	MUMPS MENINGITIS
'10081'	LEPTOSPIRAL MENINGITIS
'11283'	CANDIDAL MENINGITIS
'1142 '	COCCIDIOIDAL MENINGITIS
'11501'	HISTOPLASM CAPSUL MENING
'11511'	HISTOPLASM DUBOIS MENING
'11591'	HISTOPLASMOSIS MENINGIT
'3200 '	HEMOPHILUS MENINGITIS
'3201 '	PNEUMOCOCCAL MENINGITIS
'3202 '	STREPTOCOCCAL MENINGITIS
'3203 '	STAPHYLOCOCC MENINGITIS
'3207 '	MENING IN OTH BACT DIS
'3208 '	BACTERIAL MENINGITIS NEC (Begin 1980, End 1992)
'32081'	ANAEROBIC MENINGITIS (Begin 1992)
'32082'	GRAM NEG MENINGITIS NEC (Begin 1992)
'32089'	OTH BACTER MENINGITIS (Begin 1992)
'3209 '	BACTERIAL MENINGITIS NOS
'3210 '	CRYPTOCOCCAL MENINGITIS
'3211 '	MENING IN OTH FUNGAL DIS
'3212 '	MENING IN OTH VIRAL DIS
'3213 '	TRYPANOSOMIASIS MENINGIT
'3214 '	MENINGIT D/T SARCOIDOSIS

© 2020 Rhee C et al. *JAMA Network Open.*

'3218 '	MENING IN OTH NONBAC DIS
'3220 '	NONPYOGENIC MENINGITIS
'3221 '	EOSINOPHILIC MENINGITIS
'3222 '	CHRONIC MENINGITIS
'3229 '	MENINGITIS NOS
'0361 '	MENINGOCOCC ENCEPHALITIS
'0462 '	SUBAC SCLEROS PANENCEPH
'0498 '	VIRAL ENCEPHALITIS NEC
'0499 '	VIRAL ENCEPHALITIS NOS
'0520 '	POSTVARICELLA ENCEPHALIT
'0543 '	HERPETIC ENCEPHALITIS
'0550 '	POSTMEASLES ENCEPHALITIS
'05601'	RUBELLA ENCEPHALITIS
'05821'	HUMAN HERPESVIR 6 ENCEPH (Begin 2007)
'05829'	HUMAN HERPESVR ENCPH NEC (Begin 2007)
'0620 '	JAPANESE ENCEPHALITIS
'0621 '	WEST EQUINE ENCEPHALITIS
'0622 '	EAST EQUINE ENCEPHALITIS
'0623 '	ST LOUIS ENCEPHALITIS
'0624 '	AUSTRALIAN ENCEPHALITIS
'0625 '	CALIFORNIA ENCEPHALITIS
'0628 '	MOSQUIT-BORNE ENCEPH NEC
'0629 '	MOSQUIT-BORNE ENCEPH NOS
'0630 '	RUSSIA SPR-SUMMER ENCEPH
'0631 '	LOUPING ILL
'0632 '	CENT EUROPE ENCEPHALITIS
'0638 '	TICK-BORNE ENCEPH NEC
'0639 '	TICK-BORNE ENCEPH NOS
'064 '	VIR ENCEPH ARTHROPOD NEC
'0662 '	VENEZUELAN EQUINE FEVER
'0722 '	MUMPS ENCEPHALITIS
'1300 '	TOXOPLASM MENINGOENCEPH
'1390 '	LATE EFF VIRAL ENCEPHAL
'3230 '	ENCEPHALIT IN VIRAL DIS
'32301'	ENCEPH/ENCEPHMYE OTH DIS (Begin 2006)
'32302'	MYELITIS-OTH VIRAL DIS (Begin 2006)
'3231 '	RICKETTSIAL ENCEPHALITIS
'3232 '	PROTOZOAL ENCEPHALITIS
'3234 '	OTH ENCEPHALIT D/T INFEC
'32341'	ENCEPH/MYELITIS-OTH INF (Begin 2006)
'32342'	MYELITIS D/T OTH INFECT (Begin 2006)
'32351'	ENCEPH/MYEL FOLWG IMMUNE (Begin 2006)
'32352'	MYELITIS FOLLWG IMMUNE (Begin 2006)

© 2020 Rhee C et al. *JAMA Network Open.*

'3236 '	POSTINFECT ENCEPHALITIS
'32361'	INF AC DIS ENCEPHALOMYEL (Begin 2006)
'32362'	POSTINF ENCEPHALITIS NEC (Begin 2006)
'32363'	POSTINFECTIOUS MYELITIS (Begin 2006)
'3238 '	ENCEPHALITIS NEC
'32381'	ENCEPH & ENCEPHLALO NEC (Begin 2006)
'32382'	MYELITIS CAUSE NEC (Begin 2006)
'3239 '	ENCEPHALITIS NOS
'34120'	ACUTE MYELITIS NOS (Begin 2006)
'34121'	ACUTE MYELITIS OTH COND (Begin 2006)
'0468 '	CNS SLOW VIRUS INFEC NEC
'0469 '	CNS SLOW VIRUS INFEC NOS
'048 '	OTH ENTEROVIRAL CNS DIS
'3240 '	INTRACRANIAL ABSCESS
'3241 '	INTRASPINAL ABSCESS
'3249 '	CNS ABSCESS NOS
'326 '	LATE EFF CNS ABSCESS

C. Genitourinary

ICD-9-CM CODE	DESCRIPTION
'1122 '	CANDIDIAS UROGENITAL NEC
'0910 '	PRIMARY GENITAL SYPHILIS
'0911 '	PRIMARY ANAL SYPHILIS
'0912 '	PRIMARY SYPHILIS NEC
'0954 '	SYPHILIS OF KIDNEY
'0980 '	ACUTE GC INFECT LOWER GU
'09810'	GC (ACUTE) UPPER GU NOS
'09811'	GC CYSTITIS (ACUTE)
'09812'	GC PROSTATITIS (ACUTE)
'09813'	GC ORCHITIS (ACUTE)
'09814'	GC SEM VESICULIT (ACUTE)
'09819'	GC (ACUTE) UPPER GU NEC
'0982 '	CHR GC INFECT LOWER GU
'09830'	CHR GC UPPER GU NOS
'09831'	GC CYSTITIS- CHRONIC
'09832'	GC PROSTATITIS- CHRONIC
'09833'	GC ORCHITIS- CHRONIC
'09834'	GC SEM VESICULITIS- CHR
'09839'	CHR GC UPPER GU NEC
'0987 '	GC INFECT ANUS & RECTUM
'0990 '	CHANCROID
'0991 '	LYMPHOGRANULOMA VENEREUM

© 2020 Rhee C et al. *JAMA Network Open.*

'0992 '	GRANULOMA INGUINALE
'0994 '	NONGONOCOCC URETHRIT NEC (Begin 1980, End 1992)
'09940'	UNSPEC URETHRITIS (Begin 1992)
'09941'	CHLAMYDIA URETHRITIS (Begin 1992)
'09949'	NONGONOCOCC URETHRIT NEC (Begin 1992)
'09952'	CHLAMYDIA-ANUS RECTUM (Begin 1992)
'09953'	CHLAMYDIA-LOWER GU (Begin 1992)
'09954'	CHLAMYDIA-OTHER GU (Begin 1992)
'09955'	CHLAMYDIA-UNSPEC GU (Begin 1992)
'0998 '	VENEREAL DISEASE NEC
'0999 '	VENEREAL DISEASE NOS
'03284'	DIPHTHERITIC CYSTITIS
'59000'	CHR PYELONEPHRITIS NOS
'59001'	CHR PYELONEPH W MED NECR
‘5901’	ACUTE PYELONEPHRITIS
'59010'	AC PYELONEPHRITIS NOS
'59011'	AC PYELONEPHR W MED NECR
'5902 '	RENAL/PERIRENAL ABSCESS
'5903 '	PYELOURETERITIS CYSTICA
'59080'	PYELONEPHRITIS NOS
'59081'	PYELONEPHRIT IN OTH DIS
'5909 '	INFECTION OF KIDNEY NOS
'5950 '	ACUTE CYSTITIS
'5951 '	CHR INTERSTIT CYSTITIS
'5952 '	CHRONIC CYSTITIS NEC
'5953 '	TRIGONITIS
'5954 '	CYSTITIS IN OTH DIS
'59581'	CYSTITIS CYSTICA
'59589'	CYSTITIS NEC
'5959 '	CYSTITIS NOS
'5970 '	URETHRAL ABSCESS
'59780'	URETHRITIS NOS
'59781'	URETHRAL SYNDROME NOS
'59789'	URETHRITIS NEC
'5990 '	URIN TRACT INFECTION NOS
'6010 '	ACUTE PROSTATITIS
'6011 '	CHRONIC PROSTATITIS
'6012 '	ABSCESS OF PROSTATE
'6013 '	PROSTATOCYSTITIS
'6014 '	PROSTATITIS IN OTH DIS
'6018 '	PROSTATIC INFLAM DIS NEC
'6019 '	PROSTATITIS NOS
'6031 '	INFECTED HYDROCELE

© 2020 Rhee C et al. *JAMA Network Open.*

'6040 '	ORCHITIS WITH ABSCESS
'60490'	ORCHITIS/EPIDIDYMIT NOS
'60491'	ORCHITIS IN OTH DISEASE
'60499'	ORCHITIS/EPIDIDYMIT NEC
'6071 '	BALANOPOSTHITIS
'6072 '	INFLAM DIS- PENIS NEC
'6080 '	SEMINAL VESICULITIS
'6084 '	MALE GEN INFLAM DIS NEC
'99664'	REACT-INDWELL URIN CATH (Begin 1989)
'99665'	REACT-OTH GENITOURIN DEV (Begin 1989)

D. Intra-Abdominal

ICD-9-CM CODE	DESCRIPTION
'11285'	CANDIDAL ENTERITIS (Begin 1992)
'0952 '	SYPHILITIC PERITONITIS
'0953 '	SYPHILIS OF LIVER
'09886'	GONOCOCCAL PERITONITIS
'09956'	CHLAMYDIA-PERITONEUM (Begin 1992)
'0010 '	CHOLERA D/T VIB CHOLERAE
'0011 '	CHOLERA D/T VIB EL TOR
'0019 '	CHOLERA NOS
'0020 '	TYPHOID FEVER
'0021 '	PARATYPHOID FEVER A
'0022 '	PARATYPHOID FEVER B
'0023 '	PARATYPHOID FEVER C
'0029 '	PARATYPHOID FEVER NOS
'0030 '	SALMONELLA ENTERITIS
'00320'	LOCAL SALMONELLA INF NOS
'00329'	LOCAL SALMONELLA INF NEC
'0038 '	SALMONELLA INFECTION NEC
'0039 '	SALMONELLA INFECTION NOS
'0040 '	SHIGELLA DYSENTERIAE
'0041 '	SHIGELLA FLEXNERI
'0042 '	SHIGELLA BOYDII
'0043 '	SHIGELLA SONNEI
'0048 '	SHIGELLA INFECTION NEC
'0049 '	SHIGELLOSIS NOS
'0050 '	STAPH FOOD POISONING
'0051 '	BOTULISM
'0052 '	FOOD POIS D/T C. PERFRIN

© 2020 Rhee C et al. *JAMA Network Open.*

'0053 '	FOOD POIS: CLOSTRID NEC
'0054 '	FOOD POIS: V. PARAHAEM
'0058 '	BACT FOOD POISONING NEC (End 1995)
'00581'	FOOD POISONING VIBRIO (Begin 1995)
'00589'	OTH BACT FOOD POISONING (Begin 1995)
'0059 '	FOOD POISONING NOS
'0060 '	AC AMEBIASIS W/O ABSCESS
'0061 '	CHR AMEBIASIS W/O ABSCES
'0062 '	AMEBIC NONDYSENT COLITIS
'0063 '	AMEBIC LIVER ABSCESS
'0064 '	AMEBIC LUNG ABSCESS
'0065 '	AMEBIC BRAIN ABSCESS
'0066 '	AMEBIC SKIN ULCERATION
'0068 '	AMEBIC INFECTION NEC
'0069 '	AMEBIASIS NOS
'0070 '	BALANTIDIASIS
'0071 '	GIARDIASIS
'0072 '	COCCIDIOSIS
'0073 '	INTEST TRICHOMONIASIS
'0074 '	OT PROTOZ INTEST DX- CRYP (Begin 1997)
'0075 '	CYCLOSPORIASIS (Begin 2000)
'0078 '	PROTOZOAL INTEST DIS NEC
'0079 '	PROTOZOAL INTEST DIS NOS
'0080 '	E. COLI ENTERITIS (Begin 1980, End 1992)
'00800'	E. COLI ENTERITIS-NOS (Begin 1992)
'00801'	E. COLI ENTERITIS-PATH (Begin 1992)
'00802'	E. COLI ENTERITIS-TOX (Begin 1992)
'00803'	E. COLI ENTERITIS-INVAS (Begin 1992)
'00804'	E. COLI ENTERITIS-HEMOR (Begin 1992)
'00809'	E. COLI ENTERITIS-OTHER (Begin 1992)
'0081 '	ARIZONA ENTERITIS
'0082 '	AEROBACTER ENTERITIS
'0083 '	PROTEUS ENTERITIS
'00841'	STAPHYLOCOCC ENTERITIS
'00842'	PSEUDOMONAS ENTERITIS
'00843'	CAMPYLOBACTER (Begin 1992)
'00844'	YERSINIA (Begin 1992)
'00845'	CLOSTRIDIUM DIF (Begin 1992)
'00846'	OTHER ANAEROBES (Begin 1992)
'00847'	OTH GRAM NEG BACT (Begin 1992)
'00849'	BACTERIAL ENTERITIS NEC
'0085 '	BACTERIAL ENTERITIS NOS
'0086 '	VIRAL ENTERITIS NEC (Begin 1980, End 1992)

© 2020 Rhee C et al. *JAMA Network Open.*

'00861'	ROTAVIRUS ENTERITIS (Begin 1992)
'00862'	ADENOVIR ENTERITIS (Begin 1992)
'00863'	NORWALK VIR ENTERITIS (Begin 1992)
'00864'	SML ROUND VIR ENTERITIS (Begin 1992)
'00865'	CALCIVIRUS ENTERITIS (Begin 1992)
'00866'	ASTROVIRUS ENTERITIS (Begin 1992)
'00867'	ENTERITIS NEC (Begin 1992)
'00869'	ENTERITIS NOS (Begin 1992)
'0088 '	VIRAL ENTERITIS NOS
'0090 '	INFECTIOUS ENTERITIS NOS
'0091 '	ENTERITIS OF INFECT ORIG
'0092 '	INFECTIOUS DIARRHEA NOS
'0093 '	DIARRHEA OF INFECT ORIG
'0211 '	ENTERIC TULAREMIA
'0222 '	GASTROINTESTINAL ANTHRAX
'5400 '	AC APPEND W PERITONITIS
'5401 '	ABSCESS OF APPENDIX
'5409 '	ACUTE APPENDICITIS NOS
'541 '	APPENDICITIS NOS
'542 '	OTHER APPENDICITIS
'55000'	UNILAT ING HERNIA W GANG
'55001'	RECUR UNIL ING HERN-GANG
'55002'	BILAT ING HERNIA W GANG
'55003'	RECUR BIL ING HERN-GANG
'55100'	UNIL FEMORAL HERN W GANG
'55101'	REC UNIL FEM HERN W GANG
'55102'	BILAT FEM HERN W GANG
'55103'	RECUR BIL FEM HERN-GANG
'5511 '	UMBILICAL HERNIA W GANGR
'55120'	GANGR VENTRAL HERNIA NOS
'55121'	GANGR INCISIONAL HERNIA
'55129'	GANG VENTRAL HERNIA NEC
'5513 '	DIAPHRAGM HERNIA W GANGR
'5518 '	HERNIA- SITE NEC W GANGR
'5519 '	HERNIA- SITE NOS W GANGR
'56201'	DVRTCLI SML INT (W/O HMRG) (Begin 1980)
'56203'	DVRTCLI SML INT W HMRHG (Begin 1991)
'56211'	DVRTCLI COLON (W/O HMRHG) (Begin 1980)
'56213'	DVRTCLI COLON W HMRHG (Begin 1991)
'566 '	ANAL & RECTAL ABSCESS
'03283'	DIPHTHERITIC PERITONITIS
'5670 '	PERITONITIS IN INFEC DIS
'5671 '	PNEUMOCOCCAL PERITONITIS

© 2020 Rhee C et al. *JAMA Network Open.*

'5672 '	SUPPURAT PERITONITIS NEC (End 2005)
'56721'	PERITONITIS (ACUTE) GEN (Begin 2005)
'56722'	PERITONEAL ABSCESS (Begin 2005)
'56723'	SPONTAN BACT PERITONITIS (Begin 2005)
'56729'	SUPPURAT PERITONITIS NEC (Begin 2005)
'56738'	RETROPERITON ABSCESS NEC (Begin 2005)
'56739'	RETROPERITON INFECT NEC (Begin 2005)
'5678 '	PERITONITIS NEC (End 2005)
'56781'	CHOLEPERITONITIS (Begin 2005)
'56789'	PERITONITIS NEC (Begin 2005)
'5679 '	PERITONITIS NOS
'5695 '	INTESTINAL ABSCESS
'57400'	CHOLELITH W AC CHOLECYST
'57401'	CHOLELITH/AC GB INF-OBST
'57410'	CHOLELITH W CHOLECYS NEC
'57411'	CHOLELITH/GB INF NEC-OBS
'57421'	CHOLELITHIAS NOS W OBSTR
'57430'	CHOLEDOCHOLITH/AC GB INF
'57431'	CHOLEDOCHLITH/AC GB-OBST
'57440'	CHOLEDOCHLITH/GB INF NEC
'57441'	CHOLEDOCHLITH/GB NEC-OBS
'57451'	CHOLEDOCHLITH NOS W OBST
'57460'	CHOLELITH W/ AC W/O OBS (Begin 1996)
'57461'	CHOLELITH W/ AC W/ OBS (Begin 1996)
'57470'	CHOLELITH W/ OT W/O OBS (Begin 1996)
'57471'	CHOLELITH W/ OT W/ OBS (Begin 1996)
'57480'	CHOLELIT AC/CHR W/O OBS (Begin 1996)
'57481'	CHOLELIT AC/CHR W/ OBS (Begin 1996)
'5750 '	ACUTE CHOLECYSTITIS
'5751 '	CHOLECYSTITIS NEC (End 1996)
'57510'	CHOLECYSTITIS NOS (Begin 1996)
'57511'	CHRON CHOLECYSTITIS (Begin 1996)
'57512'	AC/CHR CHOLECYSTITIS (Begin 1996)
'5752 '	OBSTRUCTION GALLBLADDER
'5754 '	PERFORATION GALLBLADDER
'5755 '	FISTULA OF GALLBLADDER
'5761 '	CHOLANGITIS
'5762 '	OBSTRUCTION OF BILE DUCT
'5763 '	PERFORATION OF BILE DUCT
'5764 '	FISTULA OF BILE DUCT
'5720 '	ABSCESS OF LIVER
'5721 '	PORTAL PYEMIA
'56983'	PERFORATION OF INTESTINE

© 2020 Rhee C et al. *JAMA Network Open.*

'99668'	INFXN PERITON DIALY CATHET (Begin 1998)

E. Obstetric/Gynecologic

ICD-9-CM CODE	DESCRIPTION
'09815'	GC CERVICITIS (ACUTE)
'09816'	GC ENDOMETRITIS (ACUTE)
'09817'	ACUTE GC SALPINGITIS
'09835'	GC CERVICITIS- CHRONIC
'09836'	GC ENDOMETRITIS- CHRONIC
'09837'	GC SALPINGITIS (CHRONIC)
'6140 '	AC SALPINGO-OOPHORITIS
'6141 '	CHR SALPINGO-OOPHORITIS
'6142 '	SALPINGO-OOPHORITIS NOS
'6143 '	ACUTE PARAMETRITIS
'6144 '	CHRONIC PARAMETRITIS
'6145 '	AC PELV PERITONITIS-FEM
'6146 '	FEM PELVIC PERITON ADHES
'6147 '	CHR PELV PERITON NEC-FEM
'6148 '	FEM PELV INFLAM DIS NEC
'6149 '	FEM PELV INFLAM DIS NOS
'6150 '	AC UTERINE INFLAMMATION
'6151 '	CHR UTERINE INFLAMMATION
'6159 '	UTERINE INFLAM DIS NOS
'6160 '	CERVICITIS
'61610'	VAGINITIS NOS
'61611'	VAGINITIS IN OTH DISEASE
'6162 '	BARTHOLIN-s GLAND CYST
'6163 '	BARTHOLIN-s GLND ABSCESS
'6164 '	ABSCESS OF VULVA NEC
'61650'	ULCERATION OF VULVA NOS
'61651'	VULVAR ULCER IN OTH DIS
'6168 '	FEMALE GEN INFLAM NEC
'61681'	MUCOSITIS CERV
'61689'	INFLM CERV
'6169 '	FEMALE GEN INFLAM NOS
'63700'	ABORT NOS W PEL INF-UNSP
'63701'	ABORT NOS W PEL INF-INC
'63702'	ABORT NOS W PEL INF-COMP
'6380 '	ATTEM ABORT W PELVIC INF
'6390 '	POSTABORTION GU INFECT
'64660'	GU INFECT IN PREG-UNSPEC
'64661'	GU INFECTION-DELIVERED

© 2020 Rhee C et al. *JAMA Network Open.*

'64662'	GU INFECTION-DELIV W P/P
'64663'	GU INFECTION-ANTEPARTUM
'64664'	GU INFECTION-POSTPARTUM
'64700'	SYPHILIS IN PREG-UNSPEC
'64701'	SYPHILIS-DELIVERED
'64702'	SYPHILIS-DELIVERED W P/P
'64703'	SYPHILIS-ANTEPARTUM
'64704'	SYPHILIS-POSTPARTUM
'64710'	GONORRHEA IN PREG-UNSPEC
'64711'	GONORRHEA-DELIVERED
'64712'	GONORRHEA-DELIVER W P/P
'64713'	GONORRHEA-ANTEPARTUM
'64714'	GONORRHEA-POSTPARTUM
'64720'	OTHER VD IN PREG-UNSPEC
'64721'	OTHER VD-DELIVERED
'64722'	OTHER VD-DELIVERED W P/P
'64723'	OTHER VD-ANTEPARTUM
'64724'	OTHER VD-POSTPARTUM
'64730'	TB IN PREG-UNSPECIFIED
'64731'	TUBERCULOSIS-DELIVERED
'64732'	TUBERCULOSIS-DELIV W P/P
'64733'	TUBERCULOSIS-ANTEPARTUM
'64734'	TUBERCULOSIS-POSTPARTUM
'64740'	MALARIA IN PREG-UNSPEC
'64741'	MALARIA-DELIVERED
'64742'	MALARIA-DELIVERED W P/P
'64743'	MALARIA-ANTEPARTUM
'64744'	MALARIA-POSTPARTUM
'64781'	INFECT DIS NEC-DELIVERED
'64782'	INFECT DIS NEC-DEL W P/P
'64783'	INFECT DIS NEC-ANTEPART
'64784'	INFECT DIS NEC-POSTPART
'64790'	INFECT IN PREG NOS-UNSP
'64791'	INFECT NOS-DELIVERED
'64792'	INFECT NOS-DELIVER W P/P
'64793'	INFECT NOS-ANTEPARTUM
'64794'	INFECT NOS-POSTPARTUM
'67000'	MAJOR PUERP INFECT-UNSP (Begin 1991)
'67002'	MAJOR PUERP INF-DEL P/P (Begin 1991)
'67004'	MAJOR PUERP INF-POSTPART (Begin 1991)
'67330'	OB PYEMIC EMBOL-UNSPEC
'67331'	OB PYEMIC EMBOL-DELIVER
'67332'	OB PYEM EMBOL-DEL W P/P

© 2020 Rhee C et al. *JAMA Network Open.*

'67333'	OB PYEMIC EMBOL-ANTEPART
'67334'	OB PYEMIC EMBOL-POSTPART
'67500'	INFECT NIPPLE PREG-UNSP
'67501'	INFECT NIPPLE-DELIVERED
'67502'	INFECT NIPPLE-DEL W P/P
'67503'	INFECT NIPPLE-ANTEPARTUM
'67504'	INFECT NIPPLE-POSTPARTUM
'67510'	BREAST ABSCESS PREG-UNSP
'67511'	BREAST ABSCESS-DELIVERED
'67512'	BREAST ABSCESS-DEL W P/P
'67513'	BREAST ABSCESS-ANTEPART
'67514'	BREAST ABSCESS-POSTPART
'67520'	MASTITIS IN PREG-UNSPEC
'67521'	MASTITIS-DELIVERED
'67522'	MASTITIS-DELIV W P/P
'67523'	MASTITIS-ANTEPARTUM
'67524'	MASTITIS-POSTPARTUM
'67580'	BREAST INF PREG NEC-UNSP
'67581'	BREAST INFECT NEC-DELIV
'67582'	BREAST INF NEC-DEL W P/P
'67583'	BREAST INF NEC-ANTEPART
'67584'	BREAST INF NEC-POSTPART
'67590'	BREAST INF PREG NOS-UNSP
'67591'	BREAST INFECT NOS-DELIV
'67592'	BREAST INF NOS-DEL W P/P
'67593'	BREAST INF NOS-ANTEPART
'67594'	BREAST INF NOS-POSTPART

F. Pulmonary

ICD-9-CM CODE	DESCRIPTION
'0951 '	SYPHILIS OF LUNG
'00322'	SALMONELLA PNEUMONIA
'0203 '	PRIMARY PNEUMONIC PLAGUE
'0204 '	SECONDARY PNEUMON PLAGUE
'0205 '	PNEUMONIC PLAGUE NOS
'0212 '	PULMONARY TULAREMIA
'0221 '	PULMONARY ANTHRAX
'0310 '	PULMONARY MYCOBACTERIA
'0391 '	PULMONARY ACTINOMYCOSIS
'0521 '	VARICELLA PNEUMONITIS
'0551 '	POSTMEASLES PNEUMONIA
'0730 '	ORNITHOSIS PNEUMONIA

© 2020 Rhee C et al. *JAMA Network Open.*

'0830 '	Q FEVER
'1124 '	CANDIDIASIS OF LUNG
'1140 '	PRIMARY COCCIDIOIDOMYCOS
'1144 '	CHRONIC PULMON COCCIDIOIDOMYCOSIS (Begin 1993)
'1145 '	UNSPEC PULMON COCCIDIOIDOMYCOSIS (Begin 1993)
'11505'	HISTOPLASM CAPS PNEUMON
'11515'	HISTOPLASM DUB PNEUMONIA
'11595'	HISTOPLASMOSIS PNEUMONIA
'1304 '	TOXOPLASMA PNEUMONITIS
'1363 '	PNEUMOCYSTOSIS
'4800 '	ADENOVIRAL PNEUMONIA
'4801 '	RESP SYNCYT VIRAL PNEUM
'4802 '	PARINFLUENZA VIRAL PNEUM
'4803 '	PNEUMONIA DUE TO SARS-ASSOCIATED CORONAVIRUS (Begin 2003)
'4808 '	VIRAL PNEUMONIA NEC
'4809 '	VIRAL PNEUMONIA NOS
'481 '	PNEUMOCOCCAL PNEUMONIA
‘482’	OTHER BACTERIAL PNEUMONIA
'4820 '	K. PNEUMONIAE PNEUMONIA
'4821 '	PSEUDOMONAL PNEUMONIA
'4822 '	H.INFLUENZAE PNEUMONIA
'4823 '	STREPTOCOCCAL PNEUMONIA (Begin 1980, End 1992)
'48230'	STREP PNEUMONIA UNSPEC (Begin 1992)
'48231'	GRP A STREP PNEUMONIA (Begin 1992)
'48232'	GRP B STREP PNEUMONIA (Begin 1992)
'48239'	OTH STREP PNEUMONIA (Begin 1992)
'4824 '	STAPHYLOCOCCAL PNEUMONIA (End 1998)
'48240'	STAPH PNEUMONIA UNSP (Begin 1998)
'48241'	STAPH AUREUS PNEUMON (Begin 1998)
'48249'	STAPH PNEUMON OTH (Begin 1998)
'4828 '	BACTERIAL PNEUMONIA NEC (Begin 1980, End 1992)
'48281'	ANAEROBIC PNEUMONIA (Begin 1992)
'48282'	E COLI PNEUMONIA (Begin 1992)
'48283'	OTH GRAM NEG PNEUMONIA (Begin 1992)
'48284'	LEGIONNAIRES DX (Begin 1997)
'48289'	BACT PNEUMONIA NEC (Begin 1992)
'4829 '	BACTERIAL PNEUMONIA NOS
'483 '	PNEUMONIA: ORGANISM NEC (Begin 1980, End 1992)
'4830 '	MYCOPLASMA PNEUMONIA (Begin 1992)
'4831 '	CHLAMYDIA PNEUMONIA (Begin 1996)
'4838 '	OTH SPEC ORG PNEUMONIA (Begin 1992)
'4841 '	PNEUM W CYTOMEG INCL DIS
'4843 '	PNEUMONIA IN WHOOP COUGH

© 2020 Rhee C et al. *JAMA Network Open.*

'4845 '	PNEUMONIA IN ANTHRAX
'4846 '	PNEUM IN ASPERGILLOSIS
'4847 '	PNEUM IN OTH SYS MYCOSES
'4848 '	PNEUM IN INFECT DIS NEC
'485 '	BRONCOPNEUMONIA ORG NOS
'486 '	PNEUMONIA- ORGANISM NOS
'5130 '	ABSCESS OF LUNG
'5171 '	RHEUMATIC PNEUMONIA
'4870 '	INFLUENZA WITH PNEUMONIA
'4871 '	FLU W RESP MANIFEST NEC
'4878 '	FLU W MANIFESTATION NEC
'488 '	FLU D/T AVIAN FLU VIRUS (Begin 2007)
'463 '	ACUTE TONSILLITIS
'4740 '	CHRONIC TONSILLITIS (End 1997)
'47400'	CHRON TONSILLITIS (Begin 1997)
'47401'	CHRON ADENOIDITIS (Begin 1997)
'47402'	CHRON TONSIL ADENOID (Begin 1997)
'475 '	PERITONSILLAR ABSCESS
'4660 '	ACUTE BRONCHITIS
'4661 '	ACUTE BRONCHIOLITIS (End 1996)
'46611'	RSV BRONCHIOLITIS (Begin 1996)
'46619'	OTH ACUTE BRONCHIOL (Begin 1996)
'0320 '	FAUCIAL DIPHTHERIA
'0321 '	NASOPHARYNX DIPHTHERIA
'0322 '	ANT NASAL DIPHTHERIA
'0323 '	LARYNGEAL DIPHTHERIA
'0340 '	STREP SORE THROAT
'460 '	ACUTE NASOPHARYNGITIS
'4610 '	AC MAXILLARY SINUSITIS
'4611 '	AC FRONTAL SINUSITIS
'4612 '	AC ETHMOIDAL SINUSITIS
'4613 '	AC SPHENOIDAL SINUSITIS
'4618 '	OTHER ACUTE SINUSITIS
'4619 '	ACUTE SINUSITIS NOS
'462 '	ACUTE PHARYNGITIS
'4640 '	ACUTE LARYNGITIS (End 2001)
'46400'	ACUTE LARYNGITIS- W/O OBSTR (Begin 2001)
'46401'	ACUTE LARYNGITIS- W OBSTR (Begin 2001)
'46410'	AC TRACHEITIS NO OBSTRUC
'46411'	AC TRACHEITIS W OBSTRUCT
'46420'	AC LARYNGOTRACH NO OBSTR
'46421'	AC LARYNGOTRACH W OBSTR
'46430'	AC EPIGLOTTITIS NO OBSTR

© 2020 Rhee C et al. *JAMA Network Open.*

'46431'	AC EPIGLOTTITIS W OBSTR
'4644 '	CROUP
'46450'	SUPRAGLOTTIS NOS- W/O OBSTR (Begin 2001)
'46451'	SUPRAGLOTTIS NOS- W/ OBSTR (Begin 2001)
'4650 '	ACUTE LARYNGOPHARYNGITIS
'4658 '	ACUTE URI MULT SITES NEC
'4659 '	ACUTE URI NOS
'4730 '	CHR MAXILLARY SINUSITIS
'4731 '	CHR FRONTAL SINUSITIS
'4732 '	CHR ETHMOIDAL SINUSITIS
'4733 '	CHR SPHENOIDAL SINUSITIS
'4738 '	CHRONIC SINUSITIS NEC
'4739 '	CHRONIC SINUSITIS NOS-
'490 '	BRONCHITIS NOS
'49121'	OBS CHR BRNC W ACT EXA (Begin 1991)
'49122'	OBS CHR BRONC W AC BRONC (Begin 2004)
'4941 '	BRONCHIECTASIS W/ACUTE EXACERBATION (Begin 2000)
'5070 '	FOOD/VOMIT PNEUMONITIS-
'5100 '	EMPYEMA WITH FISTULA
'5109 '	EMPYEMA W/O FISTULA
'5111 '	BACT PLEUR/EFFUS NOT TB
'5131 '	ABSCESS OF MEDIASTINUM
'47871'	LARYNGEAL CELLULITIS
'5192 '	MEDIASTINITIS
‘99731’	VENTLTR ASSOC PNEUMOMINA

G. Skin/Soft Tissue

ICD-9-CM CODE	DESCRIPTION
'0392 '	ABDOMINAL ACTINOMYCOSIS
'0393 '	CERVICOFAC ACTINOMYCOSIS
'0394 '	MADURA FOOT
'0400 '	GAS GANGRENE
'04042'	WOUND BOTULISM (Begin 2007)
'04081'	TROPICAL PYOMYOSITIS
'1123 '	CUTANEOUS CANDIDIASIS
'11282'	CANDIDAL OTITIS EXTERNA
'1141 '	PRIM CUTAN COCCIDIOID
'0956 '	SYPHILIS OF MUSCLE
'0957 '	SYPHILIS OF TENDON/BURSA
'09851'	GONOCOCCAL SYNOVITIS
'09852'	GONOCOCCAL BURSITIS

© 2020 Rhee C et al. *JAMA Network Open.*

'38000'	PERICHONDRITIS PINNA NOS
'38001'	AC PERICHONDRITIS PINNA
'38002'	CHR PERICHONDRITIS PINNA
'38003'	CHONDRITIS OF PINNA (Begin 2004)
'38010'	INFEC OTITIS EXTERNA NOS
'38011'	ACUTE INFECTION OF PINNA
'38012'	ACUTE SWIMMERS EAR
'38013'	AC INFECT EXTERN EAR NEC
'38014'	MALIGNANT OTITIS EXTERNA
'38015'	CHR MYCOT OTITIS EXTERNA
'38016'	CHR INF OTIT EXTERNA NEC
'38022'	ACUTE OTITIS EXTERNA NEC
'38023'	CHR OTITIS EXTERNA NEC
'0201 '	CELLULOCUTANEOUS PLAGUE
'0210 '	ULCEROGLANDUL TULAREMIA
'0220 '	CUTANEOUS ANTHRAX
'0311 '	CUTANEOUS MYCOBACTERIA
'03285'	CUTANEOUS DIPHTHERIA
'035 '	ERYSIPELAS
'0390 '	CUTANEOUS ACTINOMYCOSIS
'6800 '	CARBUNCLE OF FACE
'6801 '	CARBUNCLE OF NECK
'6802 '	CARBUNCLE OF TRUNK
'6803 '	CARBUNCLE OF ARM
'6804 '	CARBUNCLE OF HAND
'6805 '	CARBUNCLE OF BUTTOCK
'6806 '	CARBUNCLE OF LEG
'6807 '	CARBUNCLE OF FOOT
'6808 '	CARBUNCLE- SITE NEC
'6809 '	CARBUNCLE NOS
'68100'	CELLULITIS- FINGER NOS
'68101'	FELON
'68102'	ONYCHIA OF FINGER
'68110'	CELLULITIS- TOE NOS
'68111'	ONYCHIA OF TOE
'6819 '	CELLULITIS OF DIGIT NOS
'6820 '	CELLULITIS OF FACE
'6821 '	CELLULITIS OF NECK
'6822 '	CELLULITIS OF TRUNK
'6823 '	CELLULITIS OF ARM
'6824 '	CELLULITIS OF HAND
'6825 '	CELLULITIS OF BUTTOCK
'6826 '	CELLULITIS OF LEG

© 2020 Rhee C et al. *JAMA Network Open.*

'6827 '	CELLULITIS OF FOOT
'6828 '	CELLULITIS- SITE NEC
'6829 '	CELLULITIS NOS
'684 '	IMPETIGO
'6850 '	PILONIDAL CYST W ABSCESS
'6851 '	PILONIDAL CYST W/O ABSC
'6860 '	PYODERMA (End 1997)
'68600'	PYODERMA NOS (Begin 1997)
'68601'	PYODERMA GANGREN (Begin 1997)
'68609'	PYODERMA NEC (Begin 1997)
'6861 '	PYOGENIC GRANULOMA
'6868 '	LOCAL SKIN INFECTION NEC
'6869 '	LOCAL SKIN INFECTION NOS
'56731'	PSOAS MUSCLE ABSCESS (Begin 2005)
'7280 '	INFECTIVE MYOSITIS
'72886'	NECROTIZING FASCIITIS (Begin 1995)
'99762'	INFECTION AMPUTAT STUMP
'7854 '	GANGRENE

H. Other

ICD-9-CM CODE	DESCRIPTION
'01000'	PRIM TB COMPLEX-UNSPEC
'01001'	PRIM TB COMPLEX-NO EXAM
'01002'	PRIM TB COMPLEX-EXM UNKN
'01003'	PRIM TB COMPLEX-MICRO DX
'01004'	PRIM TB COMPLEX-CULT DX
'01005'	PRIM TB COMPLEX-HISTO DX
'01006'	PRIM TB COMPLEX-OTH TEST
'01010'	PRIM TB PLEURISY-UNSPEC
'01011'	PRIM TB PLEURISY-NO EXAM
'01012'	PRIM TB PLEUR-EXAM UNKN
'01013'	PRIM TB PLEURIS-MICRO DX
'01014'	PRIM TB PLEURISY-CULT DX
'01015'	PRIM TB PLEURIS-HISTO DX
'01016'	PRIM TB PLEURIS-OTH TEST
'01080'	PRIM PROG TB NEC-UNSPEC
'01081'	PRIM PROG TB NEC-NO EXAM
'01082'	PRIM PR TB NEC-EXAM UNKN
'01083'	PRIM PRG TB NEC-MICRO DX
'01084'	PRIM PROG TB NEC-CULT DX
'01085'	PRIM PRG TB NEC-HISTO DX
'01086'	PRIM PRG TB NEC-OTH TEST
'01090'	PRIMARY TB NOS-UNSPEC
'01091'	PRIMARY TB NOS-NO EXAM

© 2020 Rhee C et al. *JAMA Network Open.*

'01092'	PRIMARY TB NOS-EXAM UNKN
'01093'	PRIMARY TB NOS-MICRO DX
'01094'	PRIMARY TB NOS-CULT DX
'01095'	PRIMARY TB NOS-HISTO DX
'01096'	PRIMARY TB NOS-OTH TEST
'01100'	TB LUNG INFILTR-UNSPEC
'01101'	TB LUNG INFILTR-NO EXAM
'01102'	TB LUNG INFILTR-EXM UNKN
'01103'	TB LUNG INFILTR-MICRO DX
'01104'	TB LUNG INFILTR-CULT DX
'01105'	TB LUNG INFILTR-HISTO DX
'01106'	TB LUNG INFILTR-OTH TEST
'01110'	TB LUNG NODULAR-UNSPEC
'01111'	TB LUNG NODULAR-NO EXAM
'01112'	TB LUNG NODUL-EXAM UNKN
'01113'	TB LUNG NODULAR-MICRO DX
'01114'	TB LUNG NODULAR-CULT DX
'01115'	TB LUNG NODULAR-HISTO DX
'01116'	TB LUNG NODULAR-OTH TEST
'01120'	TB LUNG W CAVITY-UNSPEC
'01121'	TB LUNG W CAVITY-NO EXAM
'01122'	TB LUNG CAVITY-EXAM UNKN
'01123'	TB LUNG W CAVIT-MICRO DX
'01124'	TB LUNG W CAVITY-CULT DX
'01125'	TB LUNG W CAVIT-HISTO DX
'01126'	TB LUNG W CAVIT-OTH TEST
'01130'	TB OF BRONCHUS-UNSPEC
'01131'	TB OF BRONCHUS-NO EXAM
'01132'	TB OF BRONCHUS-EXAM UNKN
'01133'	TB OF BRONCHUS-MICRO DX
'01134'	TB OF BRONCHUS-CULT DX
'01135'	TB OF BRONCHUS-HISTO DX
'01136'	TB OF BRONCHUS-OTH TEST
'01140'	TB LUNG FIBROSIS-UNSPEC
'01141'	TB LUNG FIBROSIS-NO EXAM
'01142'	TB LUNG FIBROS-EXAM UNKN
'01143'	TB LUNG FIBROS-MICRO DX
'01144'	TB LUNG FIBROSIS-CULT DX
'01145'	TB LUNG FIBROS-HISTO DX
'01146'	TB LUNG FIBROS-OTH TEST
'01150'	TB BRONCHIECTASIS-UNSPEC
'01151'	TB BRONCHIECT-NO EXAM
'01152'	TB BRONCHIECT-EXAM UNKN

© 2020 Rhee C et al. *JAMA Network Open.*

'01153'	TB BRONCHIECT-MICRO DX
'01154'	TB BRONCHIECT-CULT DX
'01155'	TB BRONCHIECT-HISTO DX
'01156'	TB BRONCHIECT-OTH TEST
'01160'	TB PNEUMONIA-UNSPEC
'01161'	TB PNEUMONIA-NO EXAM
'01162'	TB PNEUMONIA-EXAM UNKN
'01163'	TB PNEUMONIA-MICRO DX
'01164'	TB PNEUMONIA-CULT DX
'01165'	TB PNEUMONIA-HISTO DX
'01166'	TB PNEUMONIA-OTH TEST
'01170'	TB PNEUMOTHORAX-UNSPEC
'01171'	TB PNEUMOTHORAX-NO EXAM
'01172'	TB PNEUMOTHORX-EXAM UNKN
'01173'	TB PNEUMOTHORAX-MICRO DX
'01174'	TB PNEUMOTHORAX-CULT DX
'01175'	TB PNEUMOTHORAX-HISTO DX
'01176'	TB PNEUMOTHORAX-OTH TEST
'01180'	PULMONARY TB NEC-UNSPEC
'01181'	PULMONARY TB NEC-NO EXAM
'01182'	PULMON TB NEC-EXAM UNKN
'01183'	PULMON TB NEC-MICRO DX
'01184'	PULMON TB NEC-CULT DX
'01185'	PULMON TB NEC-HISTO DX
'01186'	PULMON TB NEC-OTH TEST
'01190'	PULMONARY TB NOS-UNSPEC
'01191'	PULMONARY TB NOS-NO EXAM
'01192'	PULMON TB NOS-EXAM UNKN
'01193'	PULMON TB NOS-MICRO DX
'01194'	PULMON TB NOS-CULT DX
'01195'	PULMON TB NOS-HISTO DX
'01196'	PULMON TB NOS-OTH TEST
'01200'	TB PLEURISY-UNSPEC
'01201'	TB PLEURISY-NO EXAM
'01202'	TB PLEURISY-EXAM UNKN
'01203'	TB PLEURISY-MICRO DX
'01204'	TB PLEURISY-CULT DX
'01205'	TB PLEURISY-HISTOLOG DX
'01206'	TB PLEURISY-OTH TEST
'01210'	TB THORACIC NODES-UNSPEC
'01211'	TB THORAX NODE-NO EXAM
'01212'	TB THORAX NODE-EXAM UNKN
'01213'	TB THORAX NODE-MICRO DX

© 2020 Rhee C et al. *JAMA Network Open.*

'01214'	TB THORAX NODE-CULT DX
'01215'	TB THORAX NODE-HISTO DX
'01216'	TB THORAX NODE-OTH TEST
'01220'	ISOL TRACHEAL TB-UNSPEC
'01221'	ISOL TRACHEAL TB-NO EXAM
'01222'	ISOL TRACH TB-EXAM UNKN
'01223'	ISOLAT TRACH TB-MICRO DX
'01224'	ISOL TRACHEAL TB-CULT DX
'01225'	ISOLAT TRACH TB-HISTO DX
'01226'	ISOLAT TRACH TB-OTH TEST
'01230'	TB LARYNGITIS-UNSPEC
'01231'	TB LARYNGITIS-NO EXAM
'01232'	TB LARYNGITIS-EXAM UNKN
'01233'	TB LARYNGITIS-MICRO DX
'01234'	TB LARYNGITIS-CULT DX
'01235'	TB LARYNGITIS-HISTO DX
'01236'	TB LARYNGITIS-OTH TEST
'01280'	RESP TB NEC-UNSPEC
'01281'	RESP TB NEC-NO EXAM
'01282'	RESP TB NEC-EXAM UNKN
'01283'	RESP TB NEC-MICRO DX
'01284'	RESP TB NEC-CULT DX
'01285'	RESP TB NEC-HISTO DX
'01286'	RESP TB NEC-OTH TEST
'01300'	TB MENINGITIS-UNSPEC
'01301'	TB MENINGITIS-NO EXAM
'01302'	TB MENINGITIS-EXAM UNKN
'01303'	TB MENINGITIS-MICRO DX
'01304'	TB MENINGITIS-CULT DX
'01305'	TB MENINGITIS-HISTO DX
'01306'	TB MENINGITIS-OTH TEST
'01310'	TUBRCLMA MENINGES-UNSPEC
'01311'	TUBRCLMA MENING-NO EXAM
'01312'	TUBRCLMA MENIN-EXAM UNKN
'01313'	TUBRCLMA MENING-MICRO DX
'01314'	TUBRCLMA MENING-CULT DX
'01315'	TUBRCLMA MENING-HISTO DX
'01316'	TUBRCLMA MENING-OTH TEST
'01320'	TUBERCULOMA BRAIN-UNSPEC
'01321'	TUBRCLOMA BRAIN-NO EXAM
'01322'	TUBRCLMA BRAIN-EXAM UNKN
'01323'	TUBRCLOMA BRAIN-MICRO DX
'01324'	TUBRCLOMA BRAIN-CULT DX

'01325'	TUBRCLOMA BRAIN-HISTO DX
'01326'	TUBRCLOMA BRAIN-OTH TEST
'01330'	TB BRAIN ABSCESS-UNSPEC
'01331'	TB BRAIN ABSCESS-NO EXAM
'01332'	TB BRAIN ABSC-EXAM UNKN
'01333'	TB BRAIN ABSC-MICRO DX
'01334'	TB BRAIN ABSCESS-CULT DX
'01335'	TB BRAIN ABSC-HISTO DX
'01336'	TB BRAIN ABSC-OTH TEST
'01340'	TUBRCLMA SP CORD-UNSPEC
'01341'	TUBRCLMA SP CORD-NO EXAM
'01342'	TUBRCLMA SP CD-EXAM UNKN
'01343'	TUBRCLMA SP CRD-MICRO DX
'01344'	TUBRCLMA SP CORD-CULT DX
'01345'	TUBRCLMA SP CRD-HISTO DX
'01346'	TUBRCLMA SP CRD-OTH TEST
'01350'	TB SP CRD ABSCESS-UNSPEC
'01351'	TB SP CRD ABSC-NO EXAM
'01352'	TB SP CRD ABSC-EXAM UNKN
'01353'	TB SP CRD ABSC-MICRO DX
'01354'	TB SP CRD ABSC-CULT DX
'01355'	TB SP CRD ABSC-HISTO DX
'01356'	TB SP CRD ABSC-OTH TEST
'01360'	TB ENCEPHALITIS-UNSPEC
'01361'	TB ENCEPHALITIS-NO EXAM
'01362'	TB ENCEPHALIT-EXAM UNKN
'01363'	TB ENCEPHALITIS-MICRO DX
'01364'	TB ENCEPHALITIS-CULT DX
'01365'	TB ENCEPHALITIS-HISTO DX
'01366'	TB ENCEPHALITIS-OTH TEST
'01380'	CNS TB NEC-UNSPEC
'01381'	CNS TB NEC-NO EXAM
'01382'	CNS TB NEC-EXAM UNKN
'01383'	CNS TB NEC-MICRO DX
'01384'	CNS TB NEC-CULT DX
'01385'	CNS TB NEC-HISTO DX
'01386'	CNS TB NEC-OTH TEST
'01390'	CNS TB NOS-UNSPEC
'01391'	CNS TB NOS-NO EXAM
'01392'	CNS TB NOS-EXAM UNKN
'01393'	CNS TB NOS-MICRO DX
'01394'	CNS TB NOS-CULT DX
'01395'	CNS TB NOS-HISTO DX

© 2020 Rhee C et al. *JAMA Network Open.*

'01396'	CNS TB NOS-OTH TEST
'01400'	TB PERITONITIS-UNSPEC
'01401'	TB PERITONITIS-NO EXAM
'01402'	TB PERITONITIS-EXAM UNKN
'01403'	TB PERITONITIS-MICRO DX
'01404'	TB PERITONITIS-CULT DX
'01405'	TB PERITONITIS-HISTO DX
'01406'	TB PERITONITIS-OTH TEST
'01480'	INTESTINAL TB NEC-UNSPEC
'01481'	INTESTIN TB NEC-NO EXAM
'01482'	INTEST TB NEC-EXAM UNKN
'01483'	INTESTIN TB NEC-MICRO DX
'01484'	INTESTIN TB NEC-CULT DX
'01485'	INTESTIN TB NEC-HISTO DX
'01486'	INTESTIN TB NEC-OTH TEST
'01500'	TB OF VERTEBRA-UNSPEC
'01501'	TB OF VERTEBRA-NO EXAM
'01502'	TB OF VERTEBRA-EXAM UNKN
'01503'	TB OF VERTEBRA-MICRO DX
'01504'	TB OF VERTEBRA-CULT DX
'01505'	TB OF VERTEBRA-HISTO DX
'01506'	TB OF VERTEBRA-OTH TEST
'01510'	TB OF HIP-UNSPEC
'01511'	TB OF HIP-NO EXAM
'01512'	TB OF HIP-EXAM UNKN
'01513'	TB OF HIP-MICRO DX
'01514'	TB OF HIP-CULT DX
'01515'	TB OF HIP-HISTO DX
'01516'	TB OF HIP-OTH TEST
'01520'	TB OF KNEE-UNSPEC
'01521'	TB OF KNEE-NO EXAM
'01522'	TB OF KNEE-EXAM UNKN
'01523'	TB OF KNEE-MICRO DX
'01524'	TB OF KNEE-CULT DX
'01525'	TB OF KNEE-HISTO DX
'01526'	TB OF KNEE-OTH TEST
'01550'	TB OF LIMB BONES-UNSPEC
'01551'	TB LIMB BONES-NO EXAM
'01552'	TB LIMB BONES-EXAM UNKN
'01553'	TB LIMB BONES-MICRO DX
'01554'	TB LIMB BONES-CULT DX
'01555'	TB LIMB BONES-HISTO DX
'01556'	TB LIMB BONES-OTH TEST

© 2020 Rhee C et al. *JAMA Network Open.*

'01560'	TB OF MASTOID-UNSPEC
'01561'	TB OF MASTOID-NO EXAM
'01562'	TB OF MASTOID-EXAM UNKN
'01563'	TB OF MASTOID-MICRO DX
'01564'	TB OF MASTOID-CULT DX
'01565'	TB OF MASTOID-HISTO DX
'01566'	TB OF MASTOID-OTH TEST
'01570'	TB OF BONE NEC-UNSPEC
'01571'	TB OF BONE NEC-NO EXAM
'01572'	TB OF BONE NEC-EXAM UNKN
'01573'	TB OF BONE NEC-MICRO DX
'01574'	TB OF BONE NEC-CULT DX
'01575'	TB OF BONE NEC-HISTO DX
'01576'	TB OF BONE NEC-OTH TEST
'01580'	TB OF JOINT NEC-UNSPEC
'01581'	TB OF JOINT NEC-NO EXAM
'01582'	TB JOINT NEC-EXAM UNKN
'01583'	TB OF JOINT NEC-MICRO DX
'01584'	TB OF JOINT NEC-CULT DX
'01585'	TB OF JOINT NEC-HISTO DX
'01586'	TB OF JOINT NEC-OTH TEST
'01590'	TB BONE/JOINT NOS-UNSPEC
'01591'	TB BONE/JT NOS-NO EXAM
'01592'	TB BONE/JT NOS-EXAM UNKN
'01593'	TB BONE/JT NOS-MICRO DX
'01594'	TB BONE/JT NOS-CULT DX
'01595'	TB BONE/JT NOS-HISTO DX
'01596'	TB BONE/JT NOS-OTH TEST
'01600'	TB OF KIDNEY-UNSPEC
'01601'	TB OF KIDNEY-NO EXAM
'01602'	TB OF KIDNEY-EXAM UNKN
'01603'	TB OF KIDNEY-MICRO DX
'01604'	TB OF KIDNEY-CULT DX
'01605'	TB OF KIDNEY-HISTO DX
'01606'	TB OF KIDNEY-OTH TEST
'01610'	TB OF BLADDER-UNSPEC
'01611'	TB OF BLADDER-NO EXAM
'01612'	TB OF BLADDER-EXAM UNKN
'01613'	TB OF BLADDER-MICRO DX
'01614'	TB OF BLADDER-CULT DX
'01615'	TB OF BLADDER-HISTO DX
'01616'	TB OF BLADDER-OTH TEST
'01620'	TB OF URETER-UNSPEC

© 2020 Rhee C et al. *JAMA Network Open.*

'01621'	TB OF URETER-NO EXAM
'01622'	TB OF URETER-EXAM UNKN
'01623'	TB OF URETER-MICRO DX
'01624'	TB OF URETER-CULT DX
'01625'	TB OF URETER-HISTO DX
'01626'	TB OF URETER-OTH TEST
'01630'	TB URINARY NEC-UNSPEC
'01631'	TB URINARY NEC-NO EXAM
'01632'	TB URINARY NEC-EXAM UNKN
'01633'	TB URINARY NEC-MICRO DX
'01634'	TB URINARY NEC-CULT DX
'01635'	TB URINARY NEC-HISTO DX
'01636'	TB URINARY NEC-OTH TEST
'01640'	TB EPIDIDYMIS-UNSPEC
'01641'	TB EPIDIDYMIS-NO EXAM
'01642'	TB EPIDIDYMIS-EXAM UNKN
'01643'	TB EPIDIDYMIS-MICRO DX
'01644'	TB EPIDIDYMIS-CULT DX
'01645'	TB EPIDIDYMIS-HISTO DX
'01646'	TB EPIDIDYMIS-OTH TEST
'01650'	TB MALE GENIT NEC-UNSPEC
'01651'	TB MALE GEN NEC-NO EXAM
'01652'	TB MALE GEN NEC-EX UNKN
'01653'	TB MALE GEN NEC-MICRO DX
'01654'	TB MALE GEN NEC-CULT DX
'01655'	TB MALE GEN NEC-HISTO DX
'01656'	TB MALE GEN NEC-OTH TEST
'01660'	TB OVARY & TUBE-UNSPEC
'01661'	TB OVARY & TUBE-NO EXAM
'01662'	TB OVARY/TUBE-EXAM UNKN
'01663'	TB OVARY & TUBE-MICRO DX
'01664'	TB OVARY & TUBE-CULT DX
'01665'	TB OVARY & TUBE-HISTO DX
'01666'	TB OVARY & TUBE-OTH TEST
'01670'	TB FEMALE GEN NEC-UNSPEC
'01671'	TB FEM GEN NEC-NO EXAM
'01672'	TB FEM GEN NEC-EXAM UNKN
'01673'	TB FEM GEN NEC-MICRO DX
'01674'	TB FEM GEN NEC-CULT DX
'01675'	TB FEM GEN NEC-HISTO DX
'01676'	TB FEM GEN NEC-OTH TEST
'01690'	GU TB NOS-UNSPEC
'01691'	GU TB NOS-NO EXAM

© 2020 Rhee C et al. *JAMA Network Open.*

'01692'	GU TB NOS-EXAM UNKN
'01693'	GU TB NOS-MICRO DX
'01694'	GU TB NOS-CULT DX
'01695'	GU TB NOS-HISTO DX
'01696'	GU TB NOS-OTH TEST
'01700'	TB SKIN/SUBCUTAN-UNSPEC
'01701'	TB SKIN/SUBCUT-NO EXAM
'01702'	TB SKIN/SUBCUT-EXAM UNKN
'01703'	TB SKIN/SUBCUT-MICRO DX
'01704'	TB SKIN/SUBCUT-CULT DX
'01705'	TB SKIN/SUBCUT-HISTO DX
'01706'	TB SKIN/SUBCUT-OTH TEST
'01710'	ERYTHEMA NODOS TB-UNSPEC
'01711'	ERYTHEM NODOS TB-NO EXAM
'01712'	ERYTHEM NOD TB-EXAM UNKN
'01713'	ERYTHEM NOD TB-MICRO DX
'01714'	ERYTHEM NODOS TB-CULT DX
'01715'	ERYTHEM NOD TB-HISTO DX
'01716'	ERYTHEM NOD TB-OTH TEST
'01720'	TB PERIPH LYMPH-UNSPEC
'01721'	TB PERIPH LYMPH-NO EXAM
'01722'	TB PERIPH LYMPH-EXAM UNK
'01723'	TB PERIPH LYMPH-MICRO DX
'01724'	TB PERIPH LYMPH-CULT DX
'01725'	TB PERIPH LYMPH-HISTO DX
'01726'	TB PERIPH LYMPH-OTH TEST
'01730'	TB OF EYE-UNSPEC
'01731'	TB OF EYE-NO EXAM
'01732'	TB OF EYE-EXAM UNKN
'01733'	TB OF EYE-MICRO DX
'01734'	TB OF EYE-CULT DX
'01735'	TB OF EYE-HISTO DX
'01736'	TB OF EYE-OTH TEST
'01740'	TB OF EAR-UNSPEC
'01741'	TB OF EAR-NO EXAM
'01742'	TB OF EAR-EXAM UNKN
'01743'	TB OF EAR-MICRO DX
'01744'	TB OF EAR-CULT DX
'01745'	TB OF EAR-HISTO DX
'01746'	TB OF EAR-OTH TEST
'01750'	TB OF THYROID-UNSPEC
'01751'	TB OF THYROID-NO EXAM
'01752'	TB OF THYROID-EXAM UNKN

© 2020 Rhee C et al. *JAMA Network Open.*

'01753'	TB OF THYROID-MICRO DX
'01754'	TB OF THYROID-CULT DX
'01755'	TB OF THYROID-HISTO DX
'01756'	TB OF THYROID-OTH TEST
'01760'	TB OF ADRENAL-UNSPEC
'01761'	TB OF ADRENAL-NO EXAM
'01762'	TB OF ADRENAL-EXAM UNKN
'01763'	TB OF ADRENAL-MICRO DX
'01764'	TB OF ADRENAL-CULT DX
'01765'	TB OF ADRENAL-HISTO DX
'01766'	TB OF ADRENAL-OTH TEST
'01770'	TB OF SPLEEN-UNSPEC
'01771'	TB OF SPLEEN-NO EXAM
'01772'	TB OF SPLEEN-EXAM UNKN
'01773'	TB OF SPLEEN-MICRO DX
'01774'	TB OF SPLEEN-CULT DX
'01775'	TB OF SPLEEN-HISTO DX
'01776'	TB OF SPLEEN-OTH TEST
'01780'	TB ESOPHAGUS-UNSPEC
'01781'	TB ESOPHAGUS-NO EXAM
'01782'	TB ESOPHAGUS-EXAM UNKN
'01783'	TB ESOPHAGUS-MICRO DX
'01784'	TB ESOPHAGUS-CULT DX
'01785'	TB ESOPHAGUS-HISTO DX
'01786'	TB ESOPHAGUS-OTH TEST
'01790'	TB OF ORGAN NEC-UNSPEC
'01791'	TB OF ORGAN NEC-NO EXAM
'01792'	TB ORGAN NEC-EXAM UNKN
'01793'	TB OF ORGAN NEC-MICRO DX
'01794'	TB OF ORGAN NEC-CULT DX
'01795'	TB OF ORGAN NEC-HISTO DX
'01796'	TB OF ORGAN NEC-OTH TEST
'01800'	ACUTE MILIARY TB-UNSPEC
'01801'	ACUTE MILIARY TB-NO EXAM
'01802'	AC MILIARY TB-EXAM UNKN
'01803'	AC MILIARY TB-MICRO DX
'01804'	ACUTE MILIARY TB-CULT DX
'01805'	AC MILIARY TB-HISTO DX
'01806'	AC MILIARY TB-OTH TEST
'01880'	MILIARY TB NEC-UNSPEC
'01881'	MILIARY TB NEC-NO EXAM
'01882'	MILIARY TB NEC-EXAM UNKN
'01883'	MILIARY TB NEC-MICRO DX

© 2020 Rhee C et al. *JAMA Network Open.*

'01884'	MILIARY TB NEC-CULT DX
'01885'	MILIARY TB NEC-HISTO DX
'01886'	MILIARY TB NEC-OTH TEST
'01890'	MILIARY TB NOS-UNSPEC
'01891'	MILIARY TB NOS-NO EXAM
'01892'	MILIARY TB NOS-EXAM UNKN
'01893'	MILIARY TB NOS-MICRO DX
'01894'	MILIARY TB NOS-CULT DX
'01895'	MILIARY TB NOS-HISTO DX
'01896'	MILIARY TB NOS-OTH TEST
'1370 '	LATE EFFECT TB- RESP/NOS
'1371 '	LATE EFFECT CNS TB
'1372 '	LATE EFFECT GU TB
'1373 '	LATE EFF BONE & JOINT TB
'1374 '	LATE EFFECT TB NEC
'V1201'	HX TUBERCULOSIS (Begin 1994)
'0200 '	BUBONIC PLAGUE
'0208 '	OTHER TYPES OF PLAGUE
'0209 '	PLAGUE NOS
'0218 '	TULAREMIA NEC
'0219 '	TULAREMIA NOS
'0228 '	OTHER ANTHRAX MANIFEST
'0229 '	ANTHRAX NOS
'0230 '	BRUCELLA MELITENSIS
'0231 '	BRUCELLA ABORTUS
'0232 '	BRUCELLA SUIS
'0233 '	BRUCELLA CANIS
'0238 '	BRUCELLOSIS NEC
'0239 '	BRUCELLOSIS NOS
'024 '	GLANDERS
'025 '	MELIOIDOSIS
'0260 '	SPIRILLARY FEVER
'0269 '	RAT-BITE FEVER NOS
'0270 '	LISTERIOSIS
'0271 '	ERYSIPELOTHRIX INFECTION
'0272 '	PASTEURELLOSIS
'0278 '	ZOONOTIC BACT DIS NEC
'0279 '	ZOONOTIC BACT DIS NOS
'0300 '	LEPROMATOUS LEPROSY
'0301 '	TUBERCULOID LEPROSY
'0302 '	INDETERMINATE LEPROSY
'0303 '	BORDERLINE LEPROSY
'0308 '	LEPROSY NEC

© 2020 Rhee C et al. *JAMA Network Open.*

'0309 '	LEPROSY NOS
'0312 '	DX DUE TO DISSEM MYCOBACT (Begin 1997)
'0318 '	MYCOBACTERIAL DIS NEC
'0319 '	MYCOBACTERIAL DIS NOS
'03289'	DIPHTHERIA NEC
'0329 '	DIPHTHERIA NOS
'0330 '	BORDETELLA PERTUSSIS
'0331 '	BORDETELLA PARAPERTUSSIS
'0338 '	WHOOPING COUGH NEC
'0339 '	WHOOPING COUGH NOS
'0341 '	SCARLET FEVER
'0363 '	MENINGOCOCC ADRENAL SYND
'03681'	MENINGOCOCC OPTIC NEURIT
'03689'	MENINGOCOCCAL INFECT NEC
'0369 '	MENINGOCOCCAL INFECT NOS
'037 '	TETANUS
'0398 '	ACTINOMYCOSIS NEC
'0399 '	ACTINOMYCOSIS NOS
'0401 '	RHINOSCLEROMA
'0402 '	WHIPPLE-S DISEASE
'0403 '	NECROBACILLOSIS
'04082'	TOXIC SHOCK SYNDROME (Begin 2002)
'04089'	BACTERIAL DISEASES NEC
'0410 '	STREPTOCOCCUS INFECT NOS (Begin 1980, End 1992)
'04100'	STREPTOCOCCUS UNSPEC (Begin 1992)
'04101'	GROUP A STREPTOCOCCUS (Begin 1992)
'04102'	GROUP B STREPTOCOCCUS (Begin 1992)
'04103'	GROUP C STREPTOCOCCUS (Begin 1992)
'04104'	GROUP D STREPTOCOCCUS (Begin 1992)
'04105'	GROUP G STREPTOCOCCUS (Begin 1992)
'04109'	OTHER STREPTOCOCCUS (Begin 1992)
'0411 '	STAPH INFECTION NOS (Begin 1980, End 1992)
'04110'	STAPH UNSPEC (Begin 1992)
'04111'	STAPH AUREUS (Begin 1992)
'04119'	OTHER STAPH (Begin 1992)
'0412 '	PNEUMOCOCCUS INFECT NOS
'0413 '	KLEBSIELLA INFECT NOS
'0414 '	E. COLI INFECT NOS
'0415 '	H. INFLUENZAE INFECT NOS
'0416 '	PROTEUS INFECTION NOS
'0417 '	PSEUDOMONAS INFECT NOS
'0418 '	BACTERIAL INFECTION NEC (Begin 1980, End 1992)
'04181'	MYCOPLASMA (Begin 1992)

© 2020 Rhee C et al. *JAMA Network Open.*

'04182'	BACILLUS FRAGILIS (Begin 1992)
'04183'	CLOSTRID PERFRINGENS (Begin 1992)
'04184'	OTHER ANAEROBES (Begin 1992)
'04185'	OTHER GRAM NEG ORGS (Begin 1992)
'04186'	HELICOBACTER PYLORI INFECTION (Begin 1995)
'04189'	OTHER SPEC BACTERIA (Begin 1992)
'0419 '	BACTERIAL INFECTION NOS
'390 '	RHEUM FEV W/O HRT INVOLV
'3929 '	RHEUMATIC CHOREA NOS
'7953 '	POSITIVE CULTURE FINDING (End 2002)
'79531'	NONSPECIFIC POSITIVE FINDINGS/ANTHRAX (Begin 2002)
'79539'	OTHER NONSPECIFIC POSITIVE CULTURE (Begin 2002)
'V090 '	INFEC-PENICILLIN-RESIST ORGS (Begin 1993)
'V091 '	INFEC-CEPHALOSPORIN-RESIST ORGS (Begin 1993)
'V092 '	INFEC-MACROLIDES-RESIST ORGS (Begin 1993)
'V093 '	INFEC-TETRACYCLINE-RESIST ORGS (Begin 1993)
'V094 '	INFEC-AMINOGLYCOSIDE-RESIST ORGS (Begin 1993)
'V0950'	INFEC-UNRESIST TO MULT QUINOLONES (Begin 1993)
'V0951'	INFEC-MULT QUINOLONES-RESIST ORGS (Begin 1993)
'V096 '	INFEC-SULFONAMIDES-RESIST ORGS (Begin 1993)
'V0970'	INFEC-NOT MULTI-ANTIMYCOBACT-RESIS (Begin 1993)
'V0971'	INFEC-MULTI-ANTIMYCOBAC-RESIS ORGS (Begin 1993)
'V0980'	INFEC-OTH ORGS NOT MULTIDRUG-RESIS (Begin 1993)
'V0981'	INFEC-OTH MULTI-DRUG-RESIST ORGS (Begin 1993)
'V0990'	INFEC-UNSP ORGS NOT MULTDRUG-RESIS (Begin 1993)
'V0991'	INFEC-UNSPEC MULTIDRUG-RESIST ORGS (Begin 1993)
'1125 '	DISSEMINATED CANDIDIASIS
'11284'	CANDIDAL ESOPHAGITIS (Begin 1992)
'11289'	CANDIDIASIS SITE NEC
'1129 '	CANDIDIASIS SITE NOS
'1143 '	PROGRESS COCCIDIOID NEC
'1149 '	COCCIDIOIDOMYCOSIS NOS
'11500'	HISTOPLASMA CAPSULAT NOS
'11509'	HISTOPLASMA CAPSULAT NEC
'11510'	HISTOPLASMA DUBOISII NOS
'11519'	HISTOPLASMA DUBOISII NEC
'11590'	HISTOPLASMOSIS NOS
'11599'	HISTOPLASMOSIS NEC
'1160 '	BLASTOMYCOSIS
'1161 '	PARACOCCIDIOIDOMYCOSIS
'1162 '	LOBOMYCOSIS
'1170 '	RHINOSPORIDIOSIS
'1171 '	SPOROTRICHOSIS

© 2020 Rhee C et al. *JAMA Network Open.*

'1172 '	CHROMOBLASTOMYCOSIS
'1173 '	ASPERGILLOSIS
'1174 '	MYCOTIC MYCETOMAS
'1175 '	CRYPTOCOCCOSIS
'1176 '	ALLESCHERIOSIS
'1177 '	ZYGOMYCOSIS
'1178 '	DEMATIACIOUS FUNGI INF
'1179 '	MYCOSES NEC & NOS
'118 '	OPPORTUNISTIC MYCOSES
'0500 '	VARIOLA MAJOR
'0501 '	ALASTRIM
'0502 '	MODIFIED SMALLPOX
'0509 '	SMALLPOX NOS
'0510 '	COWPOX
'0511 '	PSEUDOCOWPOX
'0512 '	CONTAGIOUS PUSTULAR DERM
'0519 '	PARAVACCINIA NOS
'0522 '	POSTVARICELLA MYELITIS (Begin 2006)
'0527 '	VARICELLA COMPLICAT NEC
'0528 '	VARICELLA COMPLICAT NOS
'0529 '	VARICELLA UNCOMPLICATED
'05310'	H ZOSTER NERV SYST NOS
'05311'	GENICULATE HERPES ZOSTER
'05312'	POSTHERPES TRIGEM NEURAL
'05313'	POSTHERPES POLYNEUROPATH
'05314'	HERPES ZOSTER MYELITIS (Begin 2006)
'05319'	H ZOSTER NERV SYST NEC
'05371'	H ZOSTER OTITIS EXTERNA
'05379'	H ZOSTER COMPLICATED NEC
'0538 '	H ZOSTER COMPLICATED NOS
'0539 '	HERPES ZOSTER NOS
'0540 '	ECZEMA HERPETICUM
'05410'	GENITAL HERPES NOS
'05411'	HERPETIC VULVOVAGINITIS
'05412'	HERPETIC ULCER OF VULVA
'05413'	HERPETIC INFECT OF PENIS
'05419'	GENITAL HERPES NEC
'0542 '	HERPETIC GINGIVOSTOMAT
'0546 '	HERPETIC WHITLOW
'05471'	VISCERAL HERPES SIMPLEX
'05473'	H SIMPLEX OTITIS EXTERNA
'05474'	HERPES SIMPLEX MYELITIS (Begin 2006)
'05479'	H SIMPLEX COMPLICAT NEC

© 2020 Rhee C et al. *JAMA Network Open.*

'0548 '	H SIMPLEX COMPLICAT NOS
'0549 '	HERPES SIMPLEX NOS
'05579'	MEASLES COMPLICATION NEC
'0558 '	MEASLES COMPLICATION NOS
'0559 '	MEASLES UNCOMPLICATED
'05600'	RUBELLA NERVE COMPL NOS
'05609'	RUBELLA NERVE COMPL NEC
'05679'	RUBELLA COMPLICATION NEC
'0568 '	RUBELLA COMPLICATION NOS
'0569 '	RUBELLA UNCOMPLICATED
'0570 '	ERYTHEMA INFECTIOSUM
'0578 '	VIRAL EXANTHEMATA NEC
'0579 '	VIRAL EXANTHEMATA NOS
'05810'	ROSEOLA INFANTUM NOS (Begin 2007)
'05811'	ROSEOLA INFANT D/T HHV-6 (Begin 2007)
'05812'	ROSEOLA INFANT D/T HHV-7 (Begin 2007)
'05881'	HUMAN HERPESVIRUS 6 INFC (Begin 2007)
'05882'	HUMAN HERPESVIRUS 7 INFC (Begin 2007)
'05889'	HUMAN HERPESVIRS INF NEC (Begin 2007)
'0600 '	SYLVATIC YELLOW FEVER
'0601 '	URBAN YELLOW FEVER
'0609 '	YELLOW FEVER NOS
'061 '	DENGUE
'0650 '	CRIMEAN HEMORRHAGIC FEV
'0651 '	OMSK HEMORRHAGIC FEVER
'0652 '	KYASANUR FOREST DISEASE
'0653 '	TICK-BORNE HEM FEVER NEC
'0654 '	MOSQUITO-BORNE HEM FEVER
'0658 '	ARTHROPOD HEM FEVER NEC
'0659 '	ARTHROPOD HEM FEVER NOS
'0660 '	PHLEBOTOMUS FEVER
'0661 '	TICK-BORNE FEVER
'0663 '	MOSQUITO-BORNE FEVER NEC
'0664 '	WEST NILE FEVER (Begin 2002, End 2004)
'06640'	WEST NILE FEVER NOS (Begin 2004)
'06641'	WEST NILE FEVER W/ENCEPH (Begin 2004)
'06642'	WEST NILE NEURO MAN NEC (Begin 2004)
'06649'	WEST NILE W COMPLIC NEC (Begin 2004)
'0668 '	ARTHROPOD VIRUS NEC
'0669 '	ARTHROPOD VIRUS NOS
'071 '	RABIES
'0720 '	MUMPS ORCHITIS
'0723 '	MUMPS PANCREATITIS

© 2020 Rhee C et al. *JAMA Network Open.*

'07271'	MUMPS HEPATITIS
'07272'	MUMPS POLYNEUROPATHY
'07279'	MUMPS COMPLICATION NEC
'0728 '	MUMPS COMPLICATION NOS
'0729 '	MUMPS UNCOMPLICATED
'0737 '	ORNITHOSIS COMPLICAT NEC
'0738 '	ORNITHOSIS COMPLICAT NOS
'0739 '	ORNITHOSIS NOS
'0740 '	HERPANGINA
'0741 '	EPIDEMIC PLEURODYNIA
'0743 '	HAND- FOOT & MOUTH DIS
'0748 '	COXSACKIE VIRUS NEC
'075 '	INFECTIOUS MONONUCLEOSIS
'0783 '	CAT-SCRATCH DISEASE
'0784 '	FOOT & MOUTH DISEASE
'0785 '	CYTOMEGAL INCLUSION DIS
'0786 '	HEM NEPHROSONEPHRITIS
'0787 '	ARENAVIRAL HEM FEVER
'07888'	OTH SPEC DISEASE DUE TO CHLAMYDIAE (Begin 1993)
'07889'	VIRAL DISEASE NEC
'0790 '	ADENOVIRUS INFECT NOS
'0791 '	ECHO VIRUS INFECT NOS
'0792 '	COXSACKIE VIRUS INF NOS
'0793 '	RHINOVIRUS INFECT NOS
'0796 '	RESPIR SYNCITIAL VIRUS (Begin 1996)
'0798 '	VIRAL INFECTION NEC (End 1993)
'07981'	HANTAVIRUS INFECTION (Begin 1995)
'07982'	SARS-ASSOCIATED CORONAVIRUS (Begin 2003)
'07983'	PARVOVIRUS B19 (Begin 2007)
'07988'	OTH SPEC CHLAMYDIAL INFECTION (Begin 1993)
'07989'	OTH SPEC VIRAL INFECTION (Begin 1993)
'0799 '	VIRAL INFECTION NOS (End 1993)
'07998'	CHLAMYDIAL INFECTION NOS (Begin 1993)
'07999'	VIRAL INFECTION NOS (Begin 1993)
'7908 '	VIREMIA NOS-
'080 '	LOUSE-BORNE TYPHUS
'0810 '	MURINE TYPHUS
'0811 '	BRILL-S DISEASE
'0812 '	SCRUB TYPHUS
'0819 '	TYPHUS NOS
'0820 '	SPOTTED FEVERS
'0821 '	BOUTONNEUSE FEVER
'0822 '	NORTH ASIAN TICK FEVER

© 2020 Rhee C et al. *JAMA Network Open.*

'0823 '	QUEENSLAND TICK TYPHUS
'08240'	EHRLICHIOSIS- UNSPECIFIED (Begin 2000)
'08241'	EHRLICHIOSIS CHAFEENSIS (Begin 2000)
'08249'	OTHER EHRLICHIOSIS (Begin 2000)
'0828 '	TICK-BORNE RICKETTS NEC
'0829 '	TICK-BORNE RICKETTS NOS
'0831 '	TRENCH FEVER
'0832 '	RICKETTSIALPOX
'0838 '	RICKETTSIOSES NEC
'0839 '	RICKETTSIOSIS NOS
'0840 '	FALCIPARUM MALARIA
'0841 '	VIVAX MALARIA
'0842 '	QUARTAN MALARIA
'0843 '	OVALE MALARIA
'0844 '	MALARIA NEC
'0845 '	MIXED MALARIA
'0846 '	MALARIA NOS
'0847 '	INDUCED MALARIA
'0848 '	BLACKWATER FEVER
'0849 '	MALARIA COMPLICATED NEC
'0850 '	VISCERAL LEISHMANIASIS
'0851 '	CUTAN LEISHMANIAS URBAN
'0852 '	CUTAN LEISHMANIAS ASIAN
'0853 '	CUTAN LEISHMANIAS ETHIOP
'0854 '	CUTAN LEISHMANIAS AMER
'0855 '	MUCOCUTAN LEISHMANIASIS
'0859 '	LEISHMANIASIS NOS
'0860 '	CHAGAS DISEASE OF HEART
'0861 '	CHAGAS DIS OF OTH ORGAN
'0862 '	CHAGAS DISEASE NOS
'0863 '	GAMBIAN TRYPANOSOMIASIS
'0864 '	RHODESIAN TRYPANOSOMIAS
'0865 '	AFRICAN TRYPANOSOMA NOS
'0869 '	TRYPANOSOMIASIS NOS
'0870 '	LOUSE-BORNE RELAPS FEVER
'0871 '	TICK-BORNE RELAPS FEVER
'0879 '	RELAPSING FEVER NOS
'0880 '	BARTONELLOSIS
'0888 '	ARTHROPOD-BORNE DIS NEC (Begin 1980, End 1989)
'08881'	LYME DISEASE (Begin 1989)
'08882'	BABESIOSIS (Begin 1993)
'08889'	OTH ARTHROPOD-BORNE DIS (Begin 1989)
'0889 '	ARTHROPOD-BORNE DIS NOS

'1000 '	LEPTOSPIROS ICTEROHEM
'10089'	LEPTOSPIRAL INFECT NEC
'1009 '	LEPTOSPIROSIS NOS
'101 '	VINCENT-S ANGINA
'1020 '	INITIAL LESIONS YAWS
'1021 '	MULTIPLE PAPILLOMATA
'1022 '	EARLY SKIN YAWS NEC
'1023 '	HYPERKERATOSIS OF YAWS
'1024 '	GUMMATA AND ULCERS- YAWS
'1025 '	GANGOSA
'1026 '	YAWS OF BONE & JOINT
'1027 '	YAWS MANIFESTATIONS NEC
'1028 '	LATENT YAWS
'1029 '	YAWS NOS
'1030 '	PINTA PRIMARY LESIONS
'1031 '	PINTA INTERMED LESIONS
'1032 '	PINTA LATE LESIONS
'1033 '	PINTA MIXED LESIONS
'1039 '	PINTA NOS
'1040 '	NONVENEREAL ENDEMIC SYPH
'1048 '	SPIROCHETAL INFECT NEC
'1049 '	SPIROCHETAL INFECT NOS
'1200 '	SCHISTOSOMA HAEMATOBIUM
'1201 '	SCHISTOSOMA MANSONI
'1202 '	SCHISTOSOMA JAPONICUM
'1203 '	CUTANEOUS SCHISTOSOMA
'1208 '	SCHISTOSOMIASIS NEC
'1209 '	SCHISTOSOMIASIS NOS
'1210 '	OPISTHORCHIASIS
'1211 '	CLONORCHIASIS
'1212 '	PARAGONIMIASIS
'1213 '	FASCIOLIASIS
'1214 '	FASCIOLOPSIASIS
'1215 '	METAGONIMIASIS
'1216 '	HETEROPHYIASIS
'1218 '	TREMATODE INFECTION NEC
'1219 '	TREMATODE INFECTION NOS
'1220 '	ECHINOCOCC GRANUL LIVER
'1221 '	ECHINOCOCC GRANUL LUNG
'1222 '	ECHINOCOCC GRAN THYROID
'1223 '	ECHINOCOCC GRANUL NEC
'1224 '	ECHINOCOCC GRANUL NOS
'1225 '	ECHINOCOC MULTILOC LIVER

© 2020 Rhee C et al. *JAMA Network Open.*

'1226 '	ECHINOCOCC MULTILOC NEC
'1227 '	ECHINOCOCC MULTILOC NOS
'1228 '	ECHINOCOCCOSIS NOS LIVER
'1229 '	ECHINOCOCCOSIS NEC/NOS
'1230 '	TAENIA SOLIUM INTESTINE
'1231 '	CYSTICERCOSIS
'1232 '	TAENIA SAGINATA INFECT
'1233 '	TAENIASIS NOS
'1234 '	DIPHYLLOBOTHRIAS INTEST
'1235 '	SPARGANOSIS
'1236 '	HYMENOLEPIASIS
'1238 '	CESTODE INFECTION NEC
'1239 '	CESTODE INFECTION NOS
'124 '	TRICHINOSIS
'1250 '	BANCROFTIAN FILARIASIS
'1251 '	MALAYAN FILARIASIS
'1252 '	LOIASIS
'1253 '	ONCHOCERCIASIS
'1254 '	DIPETALONEMIASIS
'1255 '	MANSONELLA OZZARDI INFEC
'1256 '	FILARIASIS NEC
'1257 '	DRACONTIASIS
'1259 '	FILARIASIS NOS
'1260 '	ANCYLOSTOMA DUODENALE
'1261 '	NECATOR AMERICANUS
'1262 '	ANCYLOSTOMA BRAZILIENSE
'1263 '	ANCYLOSTOMA CEYLANICUM
'1268 '	ANCYLOSTOMA NEC
'1269 '	ANCYLOSTOMIASIS NOS
'1270 '	ASCARIASIS
'1271 '	ANISAKIASIS
'1272 '	STRONGYLOIDIASIS
'1273 '	TRICHURIASIS
'1274 '	ENTEROBIASIS
'1275 '	CAPILLARIASIS
'1276 '	TRICHOSTRONGYLIASIS
'1277 '	INTEST HELMINTHIASIS NEC
'1278 '	MIXED INTESTINE HELMINTH
'1279 '	INTEST HELMINTHIASIS NOS
'1280 '	TOXOCARIASIS
'1281 '	GNATHOSTOMIASIS
'1288 '	HELMINTHIASIS NEC
'1289 '	HELMINTHIASIS NOS

© 2020 Rhee C et al. *JAMA Network Open.*

'129 '	INTESTIN PARASITISM NOS
'1305 '	TOXOPLASMA HEPATITIS
'1307 '	TOXOPLASMOSIS SITE NEC
'1308 '	MULTISYSTEM TOXOPLASMOS
'1309 '	TOXOPLASMOSIS NOS
'13100'	UROGENITAL TRICHOMON NOS
'13101'	TRICHOMONAL VAGINITIS
'13102'	TRICHOMONAL URETHRITIS
'13103'	TRICHOMONAL PROSTATITIS
'13109'	UROGENITAL TRICHOMON NEC
'1318 '	TRICHOMONIASIS NEC
'1319 '	TRICHOMONIASIS NOS
'1338 '	ACARIASIS NEC
'1339 '	ACARIASIS NOS
'1340 '	MYIASIS
'1341 '	ARTHROPOD INFEST NEC
'1342 '	HIRUDINIASIS
'1362 '	FREE-LIVING AMEBA INFECT
'1364 '	PSOROSPERMIASIS
'1365 '	SARCOSPORIDIOSIS
'1368 '	INFECT/PARASITE DIS NEC
'1369 '	INFECT/PARASITE DIS NOS
'0903 '	SYPHILITIC KERATITIS
'0913 '	SECONDARY SYPH SKIN
'0914 '	SYPHILITIC ADENOPATHY
'09150'	SYPHILITIC UVEITIS NOS
'09151'	SYPHILIT CHORIORETINITIS
'09152'	SYPHILITIC IRIDOCYCLITIS
'09161'	SYPHILITIC PERIOSTITIS
'09162'	SYPHILITIC HEPATITIS
'09169'	SECOND SYPH VISCERA NEC
'0917 '	SECOND SYPHILIS RELAPSE
'09182'	SYPHILITIC ALOPECIA
'09189'	SECONDARY SYPHILIS NEC
'0919 '	SECONDARY SYPHILIS NOS
'0930 '	AORTIC ANEURYSM- SYPHIL
'0931 '	SYPHILITIC AORTITIS
'09320'	SYPHIL ENDOCARDITIS NOS
'09321'	SYPHILITIC MITRAL VALVE
'09322'	SYPHILITIC AORTIC VALVE
'09323'	SYPHIL TRICUSPID VALVE
'09324'	SYPHIL PULMONARY VALVE
'09381'	SYPHILITIC PERICARDITIS

© 2020 Rhee C et al. *JAMA Network Open.*

'09382'	SYPHILITIC MYOCARDITIS
'09389'	CARDIOVASCULAR SYPH NEC
'0939 '	CARDIOVASCULAR SYPH NOS
'09482'	SYPHILITIC PARKINSONISM
'09483'	SYPH DISSEM RETINITIS
'09484'	SYPHILITIC OPTIC ATROPHY
'09485'	SYPH RETROBULB NEURITIS
'09486'	SYPHIL ACOUSTIC NEURITIS
'09487'	SYPH RUPT CEREB ANEURYSM
'0950 '	SYPHILITIC EPISCLERITIS
'0958 '	LATE SYMPT SYPHILIS NEC
'0959 '	LATE SYMPT SYPHILIS NOS
'0970 '	LATE SYPHILIS NOS
'0979 '	SYPHILIS NOS
'09840'	GONOCOCCAL CONJUNCTIVIT
'09841'	GONOCOCCAL IRIDOCYCLITIS
'09842'	GONOCOCCAL ENDOPHTHALMIA
'09843'	GONOCOCCAL KERATITIS
'09849'	GONOCOCCAL EYE NEC
'0986 '	GONOCOCCAL INFEC PHARYNX
'09881'	GONOCOCCAL KERATOSIS
'09883'	GONOCOCCAL PERICARDITIS
'09884'	GONOCOCCAL ENDOCARDITIS
'09885'	GONOCOCCAL HEART DIS NEC
'09889'	GONOCOCCAL INF SITE NEC
'09950'	CHLAMYDIA-UNSPEC SITE (Begin 1992)
'09951'	CHLAMYDIA-PHARYNX (Begin 1992)
'09959'	CHLAMYDIA-NSC (Begin 1992)
'0213 '	OCULOGLANDULAR TULAREMIA
'03281'	CONJUNCTIVAL DIPHTHERIA
'05320'	HERPES ZOSTER OF EYELID
'05321'	H ZOSTER KERATOCONJUNCT
'05322'	H ZOSTER IRIDOCYCLITIS
'05329'	HERPES ZOSTER OF EYE NEC
'05440'	HERPES SIMPLEX EYE NOS
'05441'	HERPES SIMPLEX OF EYELID
'05442'	DENDRITIC KERATITIS
'05443'	H SIMPLEX KERATITIS
'05444'	H SIMPLEX IRIDOCYCLITIS
'05449'	HERPES SIMPLEX EYE NEC
'05571'	MEASLES KERATITIS
'0760 '	TRACHOMA- INITIAL STAGE
'0761 '	TRACHOMA- ACTIVE STAGE

© 2020 Rhee C et al. *JAMA Network Open.*

'0769 '	TRACHOMA NOS
'0770 '	INCLUSION CONJUNCTIVITIS
'0771 '	EPIDEM KERATOCONJUNCTIV
'0772 '	PHARYNGOCONJUNCT FEVER
'0773 '	ADENOVIRAL CONJUNCT NEC
'0774 '	EPIDEM HEM CONJUNCTIVIT
'0778 '	VIRAL CONJUNCTIVITIS NEC
'0779 '	VIRAL CONJUNCTIVITIS NOS (End 1993)
'07798'	DIS OF CONJUNCT DUE TO CHLAMYDIAE (Begin 1993)
'07799'	DIS OF CONJUNCT DUE TO VIRUSES (Begin 1993)
'11502'	HISTOPLASM CAPSUL RETINA
'11512'	HISTOPLASM DUBOIS RETINA
'11592'	HISTOPLASMOSIS RETINITIS
'1301 '	TOXOPLASM CONJUNCTIVITIS
'1302 '	TOXOPLASM CHORIORETINIT
'36000'	PURULENT ENDOPHTHALM NOS
'36001'	ACUTE ENDOPHTHALMITIS
'36002'	PANOPHTHALMITIS
'36003'	CHRONIC ENDOPHTHALMITIS
'36004'	VITREOUS ABSCESS
'36013'	PARASITIC ENDOPHTHAL NOS
'36019'	ENDOPHTHALMITIS NEC
'37055'	CORNEAL ABSCESS
'37200'	ACUTE CONJUNCTIVITIS NOS
'37201'	SEROUS CONJUNCTIVITIS
'37202'	AC FOLLIC CONJUNCTIVITIS
'37203'	MUCOPUR CONJUNCTIVIT NEC
'37204'	PSEUDOMEMB CONJUNCTIVIT
'37210'	CHR CONJUNCTIVITIS NOS
'37211'	SIMPL CHR CONJUNCTIVITIS
'37212'	CHR FOLLIC CONJUNCTIVIT
'37213'	VERNAL CONJUNCTIVITIS
'37215'	PARASITIC CONJUNCTIVITIS
'37220'	BLEPHAROCONJUNCTIVIT NOS
'37221'	ANGULAR BLEPHAROCONJUNCT
'37222'	CONTACT BLEPHAROCONJUNCT
'37230'	CONJUNCTIVITIS NOS
'37239'	CONJUNCTIVITIS NEC
'37300'	BLEPHARITIS NOS
'37311'	HORDEOLUM EXTERNUM
'37312'	HORDEOLUM INTERNUM
'37313'	ABSCESS OF EYELID
'3734 '	INFECT DERM LID W DEFORM

© 2020 Rhee C et al. *JAMA Network Open.*

'3735 '	INFEC DERMATITIS LID NEC
'3736 '	PARASITIC INFEST EYELID
'37500'	DACRYOADENITIS NOS
'37501'	ACUTE DACRYOADENITIS
'37502'	CHRONIC DACRYOADENITIS
'37530'	DACRYOCYSTITIS NOS
'37531'	ACUTE CANALICULITIS
'37532'	ACUTE DACRYOCYSTITIS
'37533'	PHLEGMON DACRYOCYSTITIS
'37541'	CHRONIC CANALICULITIS
'37542'	CHRONIC DACRYOCYSTITIS
'37601'	ORBITAL CELLULITIS
'37602'	ORBITAL PERIOSTITIS
'37603'	ORBITAL OSTEOMYELITIS
'37604'	ORBITAL TENONITIS
'37612'	ORBITAL MYOSITIS
'37613'	PARASITE INFEST- ORBIT
'0552 '	POSTMEASLES OTITIS MEDIA
'38100'	AC NONSUP OTITIS MED NOS
'38101'	AC SEROUS OTITIS MEDIA
'38102'	AC MUCOID OTITIS MEDIA
'38103'	AC SANGUIN OTITIS MEDIA
'38110'	CHR SEROUS OM SIMP/NOS
'38119'	CHR SEROUS OM NEC
'38120'	CHR MUCOID OM SIMP/NOS
'38129'	CHR MUCOID OM NEC
'3813 '	CHR NONSUP OM NOS/NEC
'3814 '	NONSUPP OTITIS MEDIA NOS
'38150'	EUSTACHIAN SALPING NOS
'38151'	AC EUSTACHIAN SALPING
'38152'	CHR EUSTACHIAN SALPING
'38200'	AC SUPP OTITIS MEDIA NOS
'38201'	AC SUPP OM W DRUM RUPT
'38202'	AC SUPP OM IN OTH DIS
'3821 '	CHR TUBOTYMPAN SUPPUR OM
'3822 '	CHR ATTICOANTRAL SUP OM
'3823 '	CHR SUP OTITIS MEDIA NOS
'3824 '	SUPPUR OTITIS MEDIA NOS
'3829 '	OTITIS MEDIA NOS
'38300'	AC MASTOIDITIS W/O COMPL
'38301'	SUBPERI MASTOID ABSCESS
'38302'	AC MASTOIDITIS-COMPL NEC
'3831 '	CHRONIC MASTOIDITIS

© 2020 Rhee C et al. *JAMA Network Open.*

'38320'	PETROSITIS NOS
'38321'	ACUTE PETROSITIS
'38322'	CHRONIC PETROSITIS
'3839 '	MASTOIDITIS NOS
'42490'	ENDOCARDITIS NOS
'42491'	ENDOCARDITIS IN OTH DIS
'42499'	ENDOCARDITIS NEC
'03282'	DIPHTHERITIC MYOCARDITIS
'03640'	MENINGOCOCC CARDITIS NOS
'03641'	MENINGOCOCC PERICARDITIS
'03642'	MENINGOCOCC ENDOCARDITIS
'03643'	MENINGOCOCC MYOCARDITIS
'11281'	CANDIDAL ENDOCARDITIS
'11503'	HISTOPLASM CAPS PERICARD
'11504'	HISTOPLASM CAPS ENDOCARD
'11513'	HISTOPLASM DUB PERICARD
'11514'	HISTOPLASM DUB ENDOCARD
'11593'	HISTOPLASMOSIS PERICARD
'11594'	HISTOPLASMOSIS ENDOCARD
'1303 '	TOXOPLASMA MYOCARDITIS
'4210 '	AC/SUBAC BACT ENDOCARD
'4211 '	AC ENDOCARDIT IN OTH DIS
'4219 '	AC/SUBAC ENDOCARDIT NOS
'42292'	SEPTIC MYOCARDITIS
'41512'	SEPTIC PULMONARY EMBOLSM (Begin 2007)
'47822'	PARAPHARYNGEAL ABSCESS
'47824'	RETROPHARYNGEAL ABSCESS
'5225 '	PERIAPICAL ABSCESS
'5273 '	SALIVARY GLAND ABSCESS
'5283 '	CELLULITIS/ABSCESS MOUTH
'9966 '	INFEC/INFLAM-DEVIC/GRAFT (Begin 1980, End 1989)
'99660'	REACTION-UNSP DEVIC/GRFT (Begin 1989)
'99661'	REACT-CARDIAC DEV/GRAFT (Begin 1989)
'99662'	REACT-OTH VASC DEV/GRAFT (Begin 1989)
'99663'	REACT-NERV SYS DEV/GRAFT (Begin 1989)
'99669'	REACT-INT PROS DEVIC NEC (Begin 1989)
'99931'	INFECT D/T CENT VEN CATH (Begin 2007)
'9985 '	POSTOPERATIVE INFECTION (End 1996)
'99851'	INFECTED POSTOP SEROMA (Begin 1996)
'99859'	OTH POSTOP INFECTION (Begin 1996)
'9993 '	INFEC COMPL MED CARE NEC (end 2007)
'99939'	INFECT FOL INFUS/INJ/VAC (Begin 2007)
'683 '	ACUTE LYMPHADENITIS

© 2020 Rhee C et al. *JAMA Network Open.*

eAppendix 2. Imputations for Missing Antibiotic Susceptibilities by Pathogen

Antibiotic susceptibilities were derived from *in vitro* reports generated by each institution. Individual institutional standards on susceptibility testing were unavailable. Intermediate susceptibilities were treated as resistant. When considering whether antibiotics were active against isolated pathogens, we assessed each potential pathogen-antibiotic combination using reported antibiotic susceptibilities when available. In many cases, however, susceptibilities to specific antibiotics administered were not explicitly listed in susceptibility reports. We therefore created a set of rules to impute these susceptibilities using microbiologic principles and knowledge of the spectrum of activity for each antibiotic-pathogen combination. A "key" to the abbreviations used in these imputations is shown below. In the following sections, the algorithms for each antibiotic-pathogen imputation are shown.

Imputation Abbrevation	Explanation
R	This means that an organism will always be considered to be resistant to the antibiotic.<br>• *Example: Ceftriaxone for Pseudomonas aeruginosa will always be treated as a non-active antibiotic*
S	This means that an organism will always be considered to be susceptible to the antibiotic.<br>• *Example: Ceftriaxone for Methicillin-sensitive Staphylococcus aureus will always be treated as an active antibiotic*
if missing then E (alternate antibiotic)	This means that if the susceptibility to the antibiotic of interest is missing, then the algorithm looks at the antibiotic listed next to "E" and transposes that susceptibility.<br>• *Example: For E.coli, "E imipenem" under meropenem means that if meropenem susceptibility is missing, but imipenem is reported as susceptible, then meropenem can be considered to be susceptible too.*<br>Some of these have a further logic built it to handle cases where the other antibiotic susceptibility is missing too. These follow a hierarchial order; for example, for "E imipenem; if missing then E ertapenem" then the algorithm first looks to see if there is susceptibility reported for imipenem; if missing, then the algorithm looks for susceptibility for ertapenem.
if missing then .	This means that if the susceptibility to the antibiotic is missing, then the algorithm will list that particular antibiotic-organism combination as missing (because an imputation could not be reliably performed). In several cases, this imputation to handle an antibiotic-organism combination as missing comes only if prior imputations were unable to produce a result. Patient encounters that have missing antibiotic-organism combinations were excluded from the analysis assessing adequacy of empiric antibiotics or unnecessarily broad antibiotics.*
if missing then R	This means that the algorithm will report the organism to be resistant to the antibiotic if the susceptibility is missing.
if missing then S	This means that the algorithm will report the organism to be susceptible to the antibiotic if the susceptibility is missing.

I. GRAM-NEGATIVE ORGANISMS

A. *Acinetobacter* species

Antibiotic	Susceptibility Imputation
Amikacin	if missing then .
Amoxicillin-Clavulanate	R
Ampicillin	R
Ampicillin-Sulbactam	if missing then R
Azithromycin	R
Aztreonam	R
Cefadroxil	R
Cefepime	if missing then S if ceftriaxone-S or cefotaxime-S; else .
Ceftazidime	if missing then S if ceftriaxone-S or cefotaxime-S; else .
Ceftriaxone	if missing then E cefotaxime; if missing then R
Cefazolin	R
Cefotaxime	if missing then E ceftriaxone; if missing then R
Cefotetan	if missing then R
Cefoxitin	if missing then R
Cefpodoxime	if missing then E ceftriaxone; if missing then E cefotaxime; if missing then R
Ceftaroline	if missing then .
Cefuroxime	R
Cephalexin	R
Ciprofloxacin	if missing then .
Clarithromycin	R
Clindamycin	R
Colistin	if missing then .
Daptomycin	R
Dicloxacillin	R
Doxycycline	if missing then R
Doripenem	E meropenem
Ertapenem	if missing then .
Gentamicin	if missing then .
Imipenem	if missing then .
Levofloxacin	if missing then .
Linezolid	R
Meropenem	if missing then S if imipenem-S; if missing then .
Metronidazole	R
Minocycline	if missing then R
Moxifloxacin	R
Nafcillin	R
Oxacillin	R
Penicillin	R
Piperacillin-Tazobactam	if missing then S if piperacillin-S; if missing then .
Quinupristin-Dalfopristin	R
Tetracycline	if missing then R
Tigecycline	if missing then .
Tobramycin	if missing then .
TMP-SMX	R
Vancomycin	R

B. *Citrobacter* species

Antibiotic	Susceptibility Imputation
Amikacin	if missing then .
Amoxicillin-Clavulanate	R
Ampicillin	R
Ampicillin-Sulbactam	R
Azithromycin	R
Aztreonam	if missing then .
Cefadroxil	R
Cefepime	if missing then S if ceftriaxone-S or cefotaxime-S; else .
Ceftazidime	if missing then S if ceftriaxone-S or cefotaxime-S; else .
Ceftriaxone	if missing then E cefotaxime; if missing then .
Cefazolin	R
Cefotaxime	if missing then E ceftriaxone; if missing then .
Cefotetan	R
Cefoxitin	R
Cefpodoxime	if missing then E ceftriaxone; if missing then E cefotaxime; if missing then R
Ceftaroline	if missing then .
Cefuroxime	R
Cephalexin	R
Ciprofloxacin	if missing then .
Clarithromycin	R
Clindamycin	R
Colistin	if missing then R
Daptomycin	R
Dicloxacillin	R
Doxycycline	if missing then S if tetracycline-S; else R
Doripenem	E meropenem
Ertapenem	if missing then S if ceftriaxone-S or cefotaxime-S; else .
Gentamicin	if missing then .
Imipenem	if missing then S if ceftriaxone-S or cefotaxime-S; else .
Levofloxacin	if missing then .
Linezolid	R
Meropenem	if missing then S if imipenem-S; if missing then S if ceftriaxone-S; or cefotaxime-S else .
Metronidazole	R
Minocycline	if missing then S if tetracycline-S; else R
Moxifloxacin	if missing then .
Nafcillin	R
Oxacillin	R
Penicillin	R
Piperacillin-Tazobactam	if missing then S if piperacillin-S; if missing then S if ceftriaxone-S or cefotaxime-S; else .
Quinupristin-Dalfopristin	R
Tetracycline	if missing then R
Tigecycline	if missing then .
Tobramycin	if missing then .
TMP-SMX	if missing then S if trimethoprim-S; else .
Vancomycin	R

© 2020 Rhee C et al. *JAMA Network Open.*

C. *Enterobacter* species

Antibiotic	Susceptibility Imputation
Amikacin	if missing then .
Amoxicillin-Clavulanate	R
Ampicillin	R
Ampicillin-Sulbactam	R
Azithromycin	R
Aztreonam	if missing then .
Cefadroxil	R
Cefepime	if missing then S if ceftriaxone-S or cefotaxime-S; else .
Ceftazidime	if missing then S if ceftriaxone-S or cefotaxime-S; else .
Ceftriaxone	if missing then E cefotaxime; if missing then .
Cefazolin	R
Cefotaxime	if missing then E ceftriaxone; if missing then .
Cefotetan	R
Cefoxitin	R
Cefpodoxime	if missing then E ceftriaxone; if missing then E cefotaxime; if missing then .
Ceftaroline	if missing then .
Cefuroxime	if missing then .
Cephalexin	R
Ciprofloxacin	if missing then .
Clarithromycin	R
Clindamycin	R
Colistin	if missing then .
Daptomycin	R
Dicloxacillin	R
Doxycycline	if missing then S if tetracycline-S; else R
Doripenem	E meropenem
Ertapenem	if missing then S if ceftriaxone-S or cefotaxime-S; else .
Gentamicin	if missing then .
Imipenem	if missing then S if ceftriaxone-S or cefotaxime-S; else .
Levofloxacin	if missing then .
Linezolid	R
Meropenem	if missing then S if imipenem-S; if missing then S if ceftriaxone-S or cefotaxime-S; else .
Metronidazole	R
Minocycline	if missing then S if tetracycline-S; else R
Moxifloxacin	if missing then .
Nafcillin	R
Oxacillin	R
Penicillin	R
Piperacillin-Tazobactam	if missing then S if piperacillin-S; if missing then S if ceftriaxone-S or cefotaxime-S; else .
Quinupristin-Dalfopristin	R
Tetracycline	if missing then R
Tigecycline	if missing then .
Tobramycin	if missing then .
TMP-SMX	if missing then S if trimethoprim-S; else .

© 2020 Rhee C et al. *JAMA Network Open.*

Vancomycin	R

D. Escherichia coli

Antibiotic	Susceptibility Imputation
Amikacin	if missing then .
Amoxicillin-Clavulanate	if missing then S if ampicillin-S; else .
Ampicillin	if missing then .
Ampicillin-Sulbactam	if missing then E amoxicillin-clavulanate; if missing then S if ampicillin-S; else .
Azithromycin	if missing then E erythromycin; if missing then E clarithromycin; if missing then R
Aztreonam	if missing then E ceftriaxone; if missing then E cefotaxime; if missing then .
Cefadroxil	if missing then E cefazolin; if missing then .
Cefepime	if missing then S if ceftriaxone-S or cefotaxime-S or cefazolin-S; else .
Ceftazidime	if missing then S if ceftriaxone-S or cefotaxime-S or cefazolin-S; else .
Ceftriaxone	if missing then E cefotaxime; if missing then S if cefazolin-S; else .
Cefazolin	if missing then .
Cefotaxime	if missing then E ceftriaxone; if missing then S if cefazolin-S; else .
Cefotetan	if missing then S if cefazolin-S; else .
Cefoxitin	if missing then S if cefazolin-S; else .
Cefpodoxime	if missing then E ceftriaxone; if missing then E cefotaxime; if missing then S if cefazolin-S; if missing then .
Ceftaroline	if missing then .
Cefuroxime	if missing then S if cefazolin-S; else .
Cephalexin	if missing then .
Ciprofloxacin	if missing then .
Clarithromycin	if missing then R
Clindamycin	R
Colistin	if missing then .
Daptomycin	R
Dicloxacillin	R
Doxycycline	if missing then S if tetracycline-S; else R
Doripenem	E meropenem
Ertapenem	if missing then S if ceftriaxone-S or cefotaxime-S or cefazolin-S; else .
Gentamicin	if missing then .
Imipenem	if missing then S if ceftriaxone-S or cefotaxime-S or cefazolin-S; else .
Levofloxacin	if missing then .
Linezolid	R
Meropenem	if missing then S if imipenem-S; if missing then S if ceftriaxone-S or cefotaxime-S or cefazolin-S; else .
Metronidazole	R
Minocycline	if missing then S if tetracycline-S; else R
Moxifloxacin	if missing then .
Nafcillin	R
Oxacillin	R
Penicillin	R
Piperacillin-Tazobactam	if missing then S if piperacillin-S; if missing then S if ceftriaxone-S or cefotaxime-S or cefazolin-S; else .

© 2020 Rhee C et al. *JAMA Network Open.*

Quinupristin-Dalfopristin	R
Tetracycline	if missing then R
Tigecycline	if missing then .
Tobramycin	if missing then .
TMP-SMX	if missing then S if trimethoprim-S; else .
Vancomycin	R

E. *Klebsiella* species

Antibiotic	Susceptibility Imputation
Amikacin	if missing then .
Amoxicillin-Clavulanate	if missing then .
Ampicillin	R
Ampicillin-Sulbactam	if missing then E amoxicillin-clavulanate; if missing then .
Azithromycin	if missing then E erythromycin; if missing then E clarithromycin; if missing then R
Aztreonam	if missing then E ceftriaxone; if missing then E cefotaxime; if missing then .
Cefadroxil	if missing then E cefazolin; if missing then .
Cefepime	if missing then S if ceftriaxone-S or cefotaxime-S or cefazolin-S; else .
Ceftazidime	if missing then S if ceftriaxone-S or cefotaxime-S or cefazolin-S; else .
Ceftriaxone	if missing then E cefotaxime; if missing then S if cefazolin-S; else .
Cefazolin	if missing then .
Cefotaxime	if missing then E ceftriaxone; if missing then S if cefazolin-S; else .
Cefotetan	if missing then S if cefazolin-S; else .
Cefoxitin	if missing then S if cefazolin-S; else .
Cefpodoxime	if missing then E ceftriaxone; if missing then E cefotaxime; if missing then S if cefazolin-S; if missing then .
Ceftaroline	if missing then .
Cefuroxime	if missing then S if cefazolin-S; else .
Cephalexin	if missing then .
Ciprofloxacin	if missing then .
Clarithromycin	if missing then E erythromycin; if missing then E azithromycin; if missing then R
Clindamycin	R
Colistin	if missing then .
Daptomycin	R
Dicloxacillin	R
Doxycycline	if missing then S if tetracycline-S; else R
Doripenem	E meropenem
Ertapenem	if missing then S if ceftriaxone-S or cefotaxime-S or cefazolin-S; else .
Gentamicin	if missing then .
Imipenem	if missing then S if ceftriaxone-S or cefotaxime-S or cefazolin-S; else .
Levofloxacin	if missing then .
Linezolid	R
Meropenem	if missing then S if imipenem-S; if missing then S if ceftriaxone-S or cefotaxime-S or cefazolin-S; else .
Metronidazole	R
Minocycline	if missing then S if tetracycline-S; else .
Moxifloxacin	if missing then .
Nafcillin	R
Oxacillin	R
Penicillin	R

Piperacillin-Tazobactam	if missing then S if piperacillin-S; if missing then S if ceftriaxone-S or cefotaxime-S or cefazolin-S; else .
Quinupristin-Dalfopristin	R
Tetracycline	if missing then R
Tigecycline	if missing then .
Tobramycin	if missing then .
TMP-SMX	if missing then S if trimethoprim-S; else .
Vancomycin	R

F. *Proteus* species

Antibiotic	Susceptibility Imputation
Amikacin	if missing then .
Amoxicillin-Clavulanate	if missing then S if ampicillin-S; else .
Ampicillin	if missing then .
Ampicillin-Sulbactam	if missing then E amoxicillin-clavulanate; if missing then S if ampicillin-S; else .
Azithromycin	if missing then E erythromycin; if missing then E clarithromycin; if missing then R
Aztreonam	if missing then .
Cefadroxil	R
Cefepime	if missing then S if ceftriaxone-S or cefotaxime-S or cefazolin-S; else .
Ceftazidime	if missing then S if ceftriaxone-S or cefotaxime-S or cefazolin-S; else .
Ceftriaxone	if missing then E cefotaxime; if missing then S if cefazolin-S; else .
Cefazolin	if missing then .
Cefotaxime	if missing then E ceftriaxone; if missing then S if cefazolin-S; else .
Cefotetan	if missing then S if cefazolin-S; else .
Cefoxitin	if missing then S if cefazolin-S; else .
Cefpodoxime	if missing then E ceftriaxone; if missing then E cefotaxime; if missing then S if cefazolin-S; if missing then .
Ceftaroline	if missing then .
Cefuroxime	if missing then .
Cephalexin	if missing then E cefazolin; if missing then .
Ciprofloxacin	if missing then .
Clarithromycin	if missing then R
Clindamycin	R
Colistin	if missing then R
Daptomycin	R
Dicloxacillin	R
Doxycycline	R
Doripenem	E meropenem
Ertapenem	if missing then S if ceftriaxone-S or cefotaxime-S or cefazolin-S; else .
Gentamicin	if missing then .
Imipenem	if missing then S if ceftriaxone-S or cefotaxime-S or cefazolin-S; else .
Levofloxacin	if missing then .
Linezolid	R
Meropenem	if missing then S if imipenem-S; if missing then S if ceftriaxone-S or cefotaxime-S or cefazolin-S; else .
Metronidazole	R
Minocycline	R
Moxifloxacin	if missing then .
Nafcillin	R

© 2020 Rhee C et al. *JAMA Network Open.*

Oxacillin	R
Penicillin	R
Piperacillin-Tazobactam	if missing then S if piperacillin-S; if missing then S if ceftriaxone-S or cefotaxime-S or cefazolin-S; else .
Quinupristin-Dalfopristin	R
Tetracycline	R
Tigecycline	R
Tobramycin	if missing then .
TMP-SMX	if missing then S if trimethoprim-S; else R
Vancomycin	R

G. Pseudomonas aeruginosa

Antibiotic	Susceptibility Imputation
Amikacin	if missing then .
Amoxicillin-Clavulanate	R
Ampicillin	R
Ampicillin-Sulbactam	R
Azithromycin	R
Aztreonam	if missing then .
Cefadroxil	R
Cefepime	if missing then .
Ceftazidime	if missing then .
Ceftriaxone	R
Cefazolin	R
Cefotaxime	R
Cefotetan	R
Cefoxitin	R
Cefpodoxime	R
Ceftaroline	R
Cefuroxime	R
Cephalexin	R
Ciprofloxacin	if missing then .
Clarithromycin	R
Clindamycin	R
Colistin	if missing then .
Daptomycin	R
Dicloxacillin	R
Doxycycline	R
Doripenem	E meropenem
Ertapenem	R
Gentamicin	if missing then .
Imipenem	if missing then .
Levofloxacin	if missing then .
Linezolid	R
Meropenem	if missing then S if imipenem-S; if missing then .
Metronidazole	R
Minocycline	R
Moxifloxacin	R
Nafcillin	R
Oxacillin	R
Penicillin	R

Piperacillin-Tazobactam	if missing then S if piperacillin-S; if missing then .
Quinupristin-Dalfopristin	R
Tetracycline	R
Tigecycline	R
Tobramycin	if missing then .
TMP-SMX	R
Vancomycin	R

H. *Serratia* species

Antibiotic	Susceptibility Imputation
Amikacin	if missing then .
Amoxicillin-Clavulanate	R
Ampicillin	R
Ampicillin-Sulbactam	R
Azithromycin	R
Aztreonam	if missing then .
Cefadroxil	R
Cefepime	if missing then S if ceftriaxone-S or cefotaxime-S; else .
Ceftazidime	if missing then S if ceftriaxone-S or cefotaxime-S; else .
Ceftriaxone	if missing then E cefotaxime; if missing then .
Cefazolin	R
Cefotaxime	if missing then E ceftriaxone; if missing then .
Cefotetan	R
Cefoxitin	R
Cefpodoxime	if missing then E ceftriaxone; if missing then E cefotaxime; if missing then .
Ceftaroline	if missing then .
Cefuroxime	R
Cephalexin	R
Ciprofloxacin	if missing then .
Clarithromycin	R
Clindamycin	R
Colistin	if missing then R
Daptomycin	R
Dicloxacillin	R
Doxycycline	if missing then S if tetracycline-S; else R
Doripenem	E meropenem
Ertapenem	if missing then S if ceftriaxone-S or cefotaxime-S; else .
Gentamicin	if missing then .
Imipenem	if missing then S if ceftriaxone-S or cefotaxime-S; else .
Levofloxacin	if missing then .
Linezolid	R
Meropenem	if missing then S if imipenem-S; if missing then S if ceftriaxone-S or cefotaxime-S; else .
Metronidazole	R
Minocycline	if missing then S if tetracycline-S; else R
Moxifloxacin	if missing then .

© 2020 Rhee C et al. *JAMA Network Open.*

Nafcillin	R
Oxacillin	R
Penicillin	R
Piperacillin-Tazobactam	if missing then S if piperacillin-S; if missing then S if ceftriaxone-S or cefotaxime-S; if missing then .
Quinupristin-Dalfopristin	R
Tetracycline	if missing then R
Tigecycline	if missing then .
Tobramycin	if missing then .
TMP-SMX	if missing then S if trimethoprim-S; else R
Vancomycin	R

© 2020 Rhee C et al. *JAMA Network Open.*

II. GRAM-POSITIVE ORGANISMS

A. Penicillin-sensitive *Staphylococcus aureus*

Antibiotic	Susceptibility Imputation
Amikacin	R
Amoxicillin-Clavulanate	S
Ampicillin	S
Ampicillin-Sulbactam	S
Azithromycin	if missing then E erythromycin; if missing then .
Aztreonam	R
Cefadroxil	S
Cefepime	S
Ceftazidime	R
Ceftriaxone	S
Cefazolin	S
Cefotaxime	S
Cefotetan	S
Cefoxitin	S
Cefpodoxime	S
Ceftaroline	S
Cefuroxime	S
Cephalexin	S
Ciprofloxacin	R
Clarithromycin	if missing then E erythromycin; if missing then E azithromycin; if missing then .
Clindamycin	if missing then .
Colistin	R
Daptomycin	if missing then S
Dicloxacillin	S
Doxycycline	if missing then S if tetracycline-S; else .
Doripenem	S
Ertapenem	S
Gentamicin	R
Imipenem	S
Levofloxacin	if missing then E moxifloxacin; if missing then .
Linezolid	if missing then S
Meropenem	S
Metronidazole	R
Minocycline	if missing then S if tetracycline-S; else .
Moxifloxacin	if missing then E levofloxacin; if missing then .
Nafcillin	S
Oxacillin	S
Penicillin	S
Piperacillin-Tazobactam	S
Quinupristin-Dalfopristin	if missing then S
Tetracycline	if missing then .
Tigecycline	if missing then S
Tobramycin	R
TMP-SMX	if missing then S if trimethoprim-S; if missing then .
Vancomycin	if missing then S

© 2020 Rhee C et al. *JAMA Network Open.*

B. Methicillin-sensitive *Staphylococcus aureus*

Antibiotic	Susceptibility Imputation
Amikacin	R
Amoxicillin-Clavulanate	S
Ampicillin	R
Ampicillin-Sulbactam	S
Azithromycin	if missing then E erythromycin; if missing then .
Aztreonam	R
Cefadroxil	S
Cefepime	S
Ceftazidime	R
Ceftriaxone	S
Cefazolin	S
Cefotaxime	S
Cefotetan	S
Cefoxitin	S
Cefpodoxime	S
Ceftaroline	S
Cefuroxime	S
Cephalexin	S
Ciprofloxacin	R
Clarithromycin	if missing then E erythromycin; if missing then E azithromycin; if missing then .
Clindamycin	if missing then .
Colistin	R
Daptomycin	if missing then S
Dicloxacillin	S
Doxycycline	if missing then S if tetracycline-S; else .
Doripenem	S
Ertapenem	S
Gentamicin	R
Imipenem	S
Levofloxacin	if missing then E moxifloxacin; if missing then .
Linezolid	if missing then S
Meropenem	S
Metronidazole	R
Minocycline	if missing then S if tetracycline-S; else .
Moxifloxacin	if missing then E levofloxacin; if missing then .
Nafcillin	S
Oxacillin	S
Penicillin	R
Piperacillin-Tazobactam	S
Quinupristin-Dalfopristin	if missing then S
Tetracycline	if missing then .
Tigecycline	if missing then S
Tobramycin	R
TMP-SMX	if missing then S if trimethoprim-S; if missing then .
Vancomycin	if missing then S

© 2020 Rhee C et al. *JAMA Network Open.*

C. Methicillin-Resistant *Staphylococcus aureus*

Antibiotic	Susceptibility Imputation
Amikacin	R
Amoxicillin-Clavulanate	R
Ampicillin	R
Ampicillin-Sulbactam	R
Azithromycin	if missing then E erythromycin; if missing then .
Aztreonam	R
Cefadroxil	R
Cefepime	R
Ceftazidime	R
Ceftriaxone	R
Cefazolin	R
Cefotaxime	R
Cefotetan	R
Cefoxitin	R
Cefpodoxime	R
Ceftaroline	S
Cefuroxime	R
Cephalexin	R
Ciprofloxacin	R
Clarithromycin	if missing then E erythromycin; if missing then E azithromycin; if missing then .
Clindamycin	if missing then .
Colistin	R
Daptomycin	if missing then S
Dicloxacillin	R
Doxycycline	if missing then S if tetracycline-S; else .
Doripenem	R
Ertapenem	R
Gentamicin	R
Imipenem	R
Levofloxacin	if missing then E moxifloxacin; if missing then .
Linezolid	if missing then S
Meropenem	R
Metronidazole	R
Minocycline	if missing then S if tetracycline-S; else .
Moxifloxacin	if missing then E levofloxacin; if missing then .
Nafcillin	R
Oxacillin	R
Penicillin	R
Piperacillin-Tazobactam	R
Quinupristin-Dalfopristin	if missing then S
Tetracycline	if missing then .
Tigecycline	if missing then S
Tobramycin	R
TMP-SMX	if missing then S if trimethoprim-S; if missing then .

© 2020 Rhee C et al. *JAMA Network Open.*

Vancomycin	if missing then S

D. ***Streptococcus pneumoniae***

Antibiotic	Susceptibility Imputation
Amikacin	R
Amoxicillin-Clavulanate	if missing then S if penicillin-S; if missing then .
Ampicillin	if missing then E penicillin; if missing then .
Ampicillin-Sulbactam	if missing then S if penicillin-S; if missing then .
Azithromycin	if missing then E erythromycin; if missing then .
Aztreonam	R
Cefadroxil	if missing then E cefazolin; if missing then S if penicillin-S; if missing then .
Cefepime	if missing then E ceftriaxone; if missing then S if penicillin-S; if missing then .
Ceftazidime	R
Ceftriaxone	if missing then E cefotaxime; if missing then S if penicillin-S; if missing then .
Cefazolin	if missing then S if penicillin-S; if missing then .
Cefotaxime	if missing then E ceftriaxone; if missing then S if penicillin-S; if missing then .
Cefotetan	if missing then R
Cefoxitin	if missing then R
Cefpodoxime	if missing then E ceftriaxone; if missing then E cefotaxime; if missing then S if penicillin-S; if missing then .
Ceftaroline	if missing then S
Cefuroxime	if missing then S if penicillin-S; if missing then .
Cephalexin	if missing then S if penicillin-S; if missing then .
Ciprofloxacin	R
Clarithromycin	if missing then E erythromycin; if missing then E azithromycin; if missing then .
Clindamycin	if missing then .
Colistin	R
Daptomycin	if missing then S
Dicloxacillin	if missing then S if penicillin-S; if missing then .
Doxycycline	if missing then S if tetracycline-S; else .
Doripenem	E meropenem
Ertapenem	if missing then S if ceftriaxone-S; if missing then S if cefotaxime-S; if missing then S if penicillin-S; if missing then .
Gentamicin	R
Imipenem	if missing then S if ceftriaxone-S; if missing then S if cefotaxime-S; if missing then S if penicillin-S; if missing then .
Levofloxacin	if missing then .
Linezolid	if missing then S
Meropenem	if missing then S if ceftriaxone-S; if missing then S if cefotaxime-S; if missing then S if penicillin-S; if missing then .
Metronidazole	R
Minocycline	if missing then S if tetracycline-S; else .
Moxifloxacin	if missing then E levofloxacin; if missing then .
Nafcillin	if missing then S if penicillin-S; if missing then .
Oxacillin	if missing then S if penicillin-S; if missing then .
Penicillin	if missing then .
Piperacillin-Tazobactam	if missing then S if ceftriaxone-S; if missing then S if cefotaxime-S; if missing

© 2020 Rhee C et al. *JAMA Network Open.*

	then S if penicillin-S; if missing then .
Quinupristin-Dalfopristin	S
Tetracycline	if missing then .
Tigecycline	S
Tobramycin	R
TMP-SMX	if missing then S if trimethoprim-S; if missing then .
Vancomycin	S

E. Beta-Hemolytic *Streptococcus* species

Antibiotic	Susceptibility Imputation
Amikacin	R
Amoxicillin-Clavulanate	if missing then S if penicillin-S; if missing then .
Ampicillin	if missing then E penicillin; if missing then .
Ampicillin-Sulbactam	if missing then S if penicillin-S; if missing then .
Azithromycin	if missing then E erythromycin; if missing then .
Aztreonam	R
Cefadroxil	if missing then E cefazolin; if missing then S if penicillin-S; if missing then .
Cefepime	S
Ceftazidime	R
Ceftriaxone	S
Cefazolin	if missing then S if penicillin-S; if missing then .
Cefotaxime	S
Cefotetan	R
Cefoxitin	R
Cefpodoxime	S
Ceftaroline	S
Cefuroxime	S
Cephalexin	if missing then S if penicillin-S; if missing then .
Ciprofloxacin	R
Clarithromycin	if missing then E erythromycin; if missing then E azithromycin; if missing then .
Clindamycin	if missing then .
Colistin	R
Daptomycin	if missing then S
Dicloxacillin	if missing then S if penicillin-S; if missing then .
Doxycycline	if missing then S if tetracycline-S; else .
Doripenem	S
Ertapenem	S
Gentamicin	R
Imipenem	S
Levofloxacin	if missing then S
Linezolid	S
Meropenem	S
Metronidazole	R
Minocycline	if missing then S if tetracycline-S; else .
Moxifloxacin	if missing then E levofloxacin; if missing then S
Nafcillin	if missing then S if penicillin-S; if missing then .
Oxacillin	if missing then S if penicillin-S; if missing then .
Penicillin	if missing then S
Piperacillin-Tazobactam	if missing then S
Quinupristin-Dalfopristin	S
Tetracycline	if missing then .

© 2020 Rhee C et al. *JAMA Network Open.*

Tigecycline	S
Tobramycin	R
TMP-SMX	if missing then S if trimethoprim-S; if missing then .
Vancomycin	S

F. Alpha-hemolytic / Viridans *Streptococcus* species

Antibiotic	Susceptibility Imputation
Amikacin	R
Amoxicillin-Clavulanate	if missing then S if penicillin-S; if missing then .
Ampicillin	if missing then E penicillin; if missing then .
Ampicillin-Sulbactam	if missing then S if penicillin-S; if missing then .
Azithromycin	if missing then E erythromycin; if missing then .
Aztreonam	R
Cefadroxil	if missing then E cefazolin; if missing then S if penicillin-S; if missing then .
Cefepime	if missing then E ceftriaxone; if missing then S if penicillin-S; if missing then .
Ceftazidime	R
Ceftriaxone	if missing then E cefotaxime; if missing then S if penicillin-S; if missing then .
Cefazolin	if missing then S if penicillin-S; if missing then .
Cefotaxime	if missing then E ceftriaxone; if missing then S if penicillin-S; if missing then .
Cefotetan	R
Cefoxitin	if missing then R
Cefpodoxime	if missing then E ceftriaxone; if missing then E cefotaxime; if missing then S if penicillin-S; if missing then .
Ceftaroline	if missing then S
Cefuroxime	if missing then S if penicillin-S; if missing then .
Cephalexin	if missing then S if penicillin-S; if missing then .
Ciprofloxacin	R
Clarithromycin	if missing then .
Clindamycin	if missing then .
Colistin	R
Daptomycin	if missing then S
Dicloxacillin	if missing then S if penicillin-S; if missing then .
Doxycycline	if missing then S if tetracycline-S; else R
Doripenem	E meropenem
Ertapenem	if missing then S if ceftriaxone-S; if missing then S if cefotaxime-S; if missing then S if penicillin-S; if missing then .
Gentamicin	R
Imipenem	if missing then S if ceftriaxone-S; if missing then S if cefotaxime-S; if missing then S if penicillin-S; if missing then .
Levofloxacin	if missing then .
Linezolid	S
Meropenem	if missing then S if ceftriaxone-S; if missing then S if cefotaxime-S; if missing then S if penicillin-S; if missing then .
Metronidazole	R
Minocycline	if missing then S if tetracycline-S; else .
Moxifloxacin	if missing then E levofloxacin; if missing then .
Nafcillin	if missing then S if penicillin-S; if missing then .

© 2020 Rhee C et al. *JAMA Network Open.*

Oxacillin	if missing then S if penicillin-S; if missing then .
Penicillin	if missing then .
Piperacillin-Tazobactam	if missing then S if ceftriaxone-S; if missing then S if cefotaxime-S; if missing then S if penicillin-S; if missing then .
Quinupristin-Dalfopristin	S
Tetracycline	if missing then R
Tigecycline	if missing then S
Tobramycin	R
TMP-SMX	if missing then S if trimethoprim-S; if missing then .
Vancomycin	if missing then S

G. *Enterococcus* species

Antibiotic	Susceptibility Imputation
Amikacin	R
Amoxicillin-Clavulanate	if missing then E ampicillin; if missing then E penicillin; if missing then .
Ampicillin	if missing then E penicillin; if missing then .
Ampicillin-Sulbactam	if missing then E ampicillin; if missing then E penicillin; if missing then .
Azithromycin	R
Aztreonam	R
Cefadroxil	R
Cefepime	R
Ceftazidime	R
Ceftriaxone	R
Cefazolin	R
Cefotaxime	R
Cefotetan	R
Cefoxitin	R
Cefpodoxime	R
Ceftaroline	if missing then S
Cefuroxime	R
Cephalexin	R
Ciprofloxacin	R
Clarithromycin	R
Clindamycin	R
Colistin	R
Daptomycin	if missing then S
Dicloxacillin	R
Doxycycline	R
Doripenem	E meropenem
Ertapenem	R
Gentamicin	R
Imipenem	if missing then E ampicillin; if missing then E penicillin; if missing then .
Levofloxacin	R
Linezolid	if missing then S
Meropenem	if missing then E ampicillin; if missing then E penicillin; if missing then .
Metronidazole	R
Minocycline	R
Moxifloxacin	R
Nafcillin	R
Oxacillin	R
Penicillin	if missing then .

Piperacillin-Tazobactam	if missing then E ampicillin; if missing then E penicillin; if missing then .
Quinupristin-Dalfopristin	if missing then S
Tetracycline	R
Tigecycline	if missing then S
Tobramycin	R
TMP-SMX	R
Vancomycin	if missing then .

© 2020 Rhee C et al. *JAMA Network Open.*

eAppendix 3. Multiple Imputation Methods for Missing Data in Severity-of-Illness Covariates

For missing severity-of-illness covariates (all of which were continuous variables) included in the logistic regression models, we used the predictive mean matching method for imputation to avoid biologically impossible predictions (e.g., outside of plausible range), implemented using the MI procedure in SAS. For each missing value, the method imputes a value randomly selected from a set of observed values whose predicted values are close to the predicted value for the missing value from the simulated regression model (Heitjan and Little 1991; Schenker and Taylor 1996). Five imputed datasets were generated. Results were pooled using Proc MIANALYZE where the final estimates were the average of estimates from 5 model fits and the covariance was calculated based on Rubin's rule (Rubin 1987, p.137).

Heitjan, F. and Little, R. J. A. (1991), "Multiple Imputation for the Fatal Accident Reporting System," *Applied Statistics*, 40, 13–29.
Schenker, N. and Taylor, J. M. G. (1996), "Partially Parametric Techniques for Multiple Imputation," *Computational Statistics and Data Analysis*, 22, 425–446.
Rubin, D. B. (1987). *Multiple Imputation for Nonresponse in Surveys*. New York: John Wiley & Sons.

© 2020 Rhee C et al. *JAMA Network Open.*

eFigure 1. Prevalence of Pathogens by Culture Site (Blood, Urine, Respiratory) for Patients With Community-Onset Sepsis

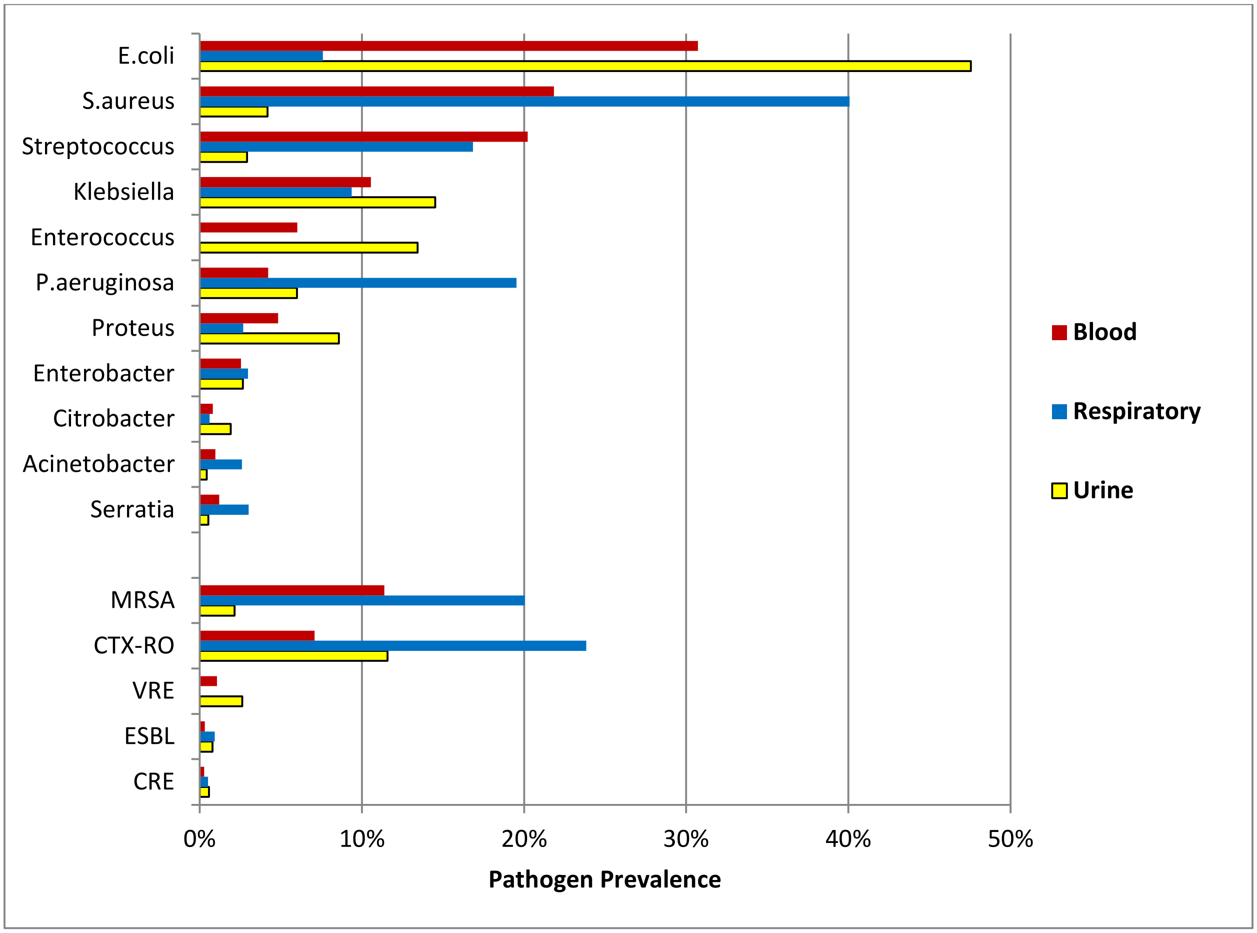

Abbreviations: MRSA = methicillin-resistant *Staphylococcus aureus*, CTX-RO = ceftriaxone-resistant gram-negative organism, VRE = vancomycin-resistant Enterococcus, ESBL = extended-spectrum beta-lactamase producing gram-negative organism, CRE = carbapenem-resistant Enterobacteriaceae.

Note: the denominators varied for each culture site: blood, n=6,968; respiratory, n=2,912; urine, n=9,077. Only cultures taken within the first two days of hospitalization were included in the analysis. Pathogens from other culture sites (deep tissue, central nervous system, body fluid, and superficial tissue) are not shown as they accounted for a very low proportion of all positive cultures.

© 2020 Rhee C et al. *JAMA Network Open.*

eFigure 2. Prevalence of Resistant Organisms in Septic Shock vs Sepsis Without Shock

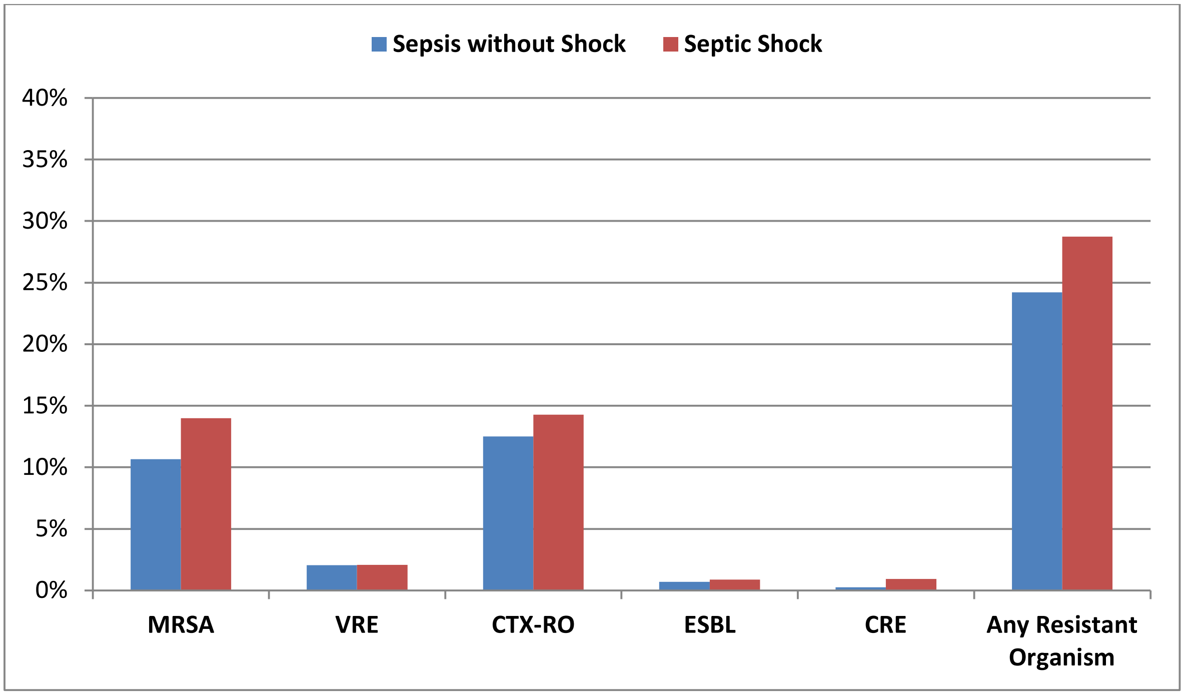

Abbreviations: MRSA = Methicillin-resistant *Staphylococcus aureus*, VRE = Vancomycin-resistant Enterococcus, CTX-RO = ceftriaxone-resistant gram-negative organisms (including *Pseudomonas aeruginosa*), ESBL = extended-spectrum beta-lactamase producing gram-negative, CRE = carbapenem-resistant Enterobacteriaceae. Septic shock was defined by the need for vasopressors among patients with presumed serious infection on admission.

© 2020 Rhee C et al. *JAMA Network Open.*

eFigure 3. Frequency of Empiric Antibiotic Choices in Culture-Positive Community-Onset Sepsis

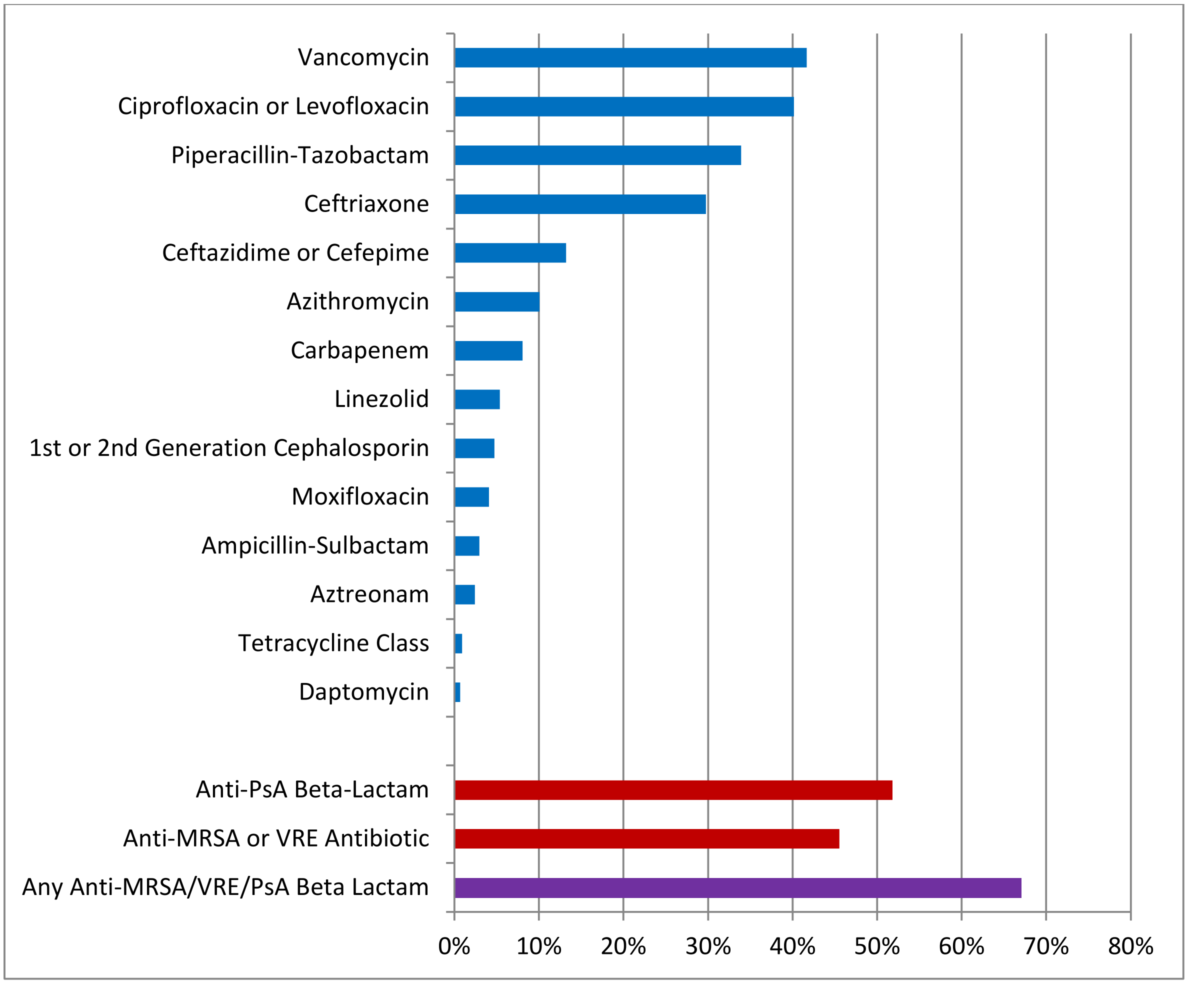

Methicillin-resistant *Staphylococcus aureus* (MRSA) antibiotics = vancomycin, linezolid, and daptomycin.

Vancomycin-resistant Enterococcus (VRE) antibiotics = linezolid or daptomycin.

Anti-Pseudomonal (PsA) beta-lactams = ceftazidime, cefepime, piperacillin-tazobactam, aztreonam, imipenem, meropenem, or doripenem.

© 2020 Rhee C et al. *JAMA Network Open.*

eFigure 4. Quantity of Missing Data for Severity-of-Illness Covariates

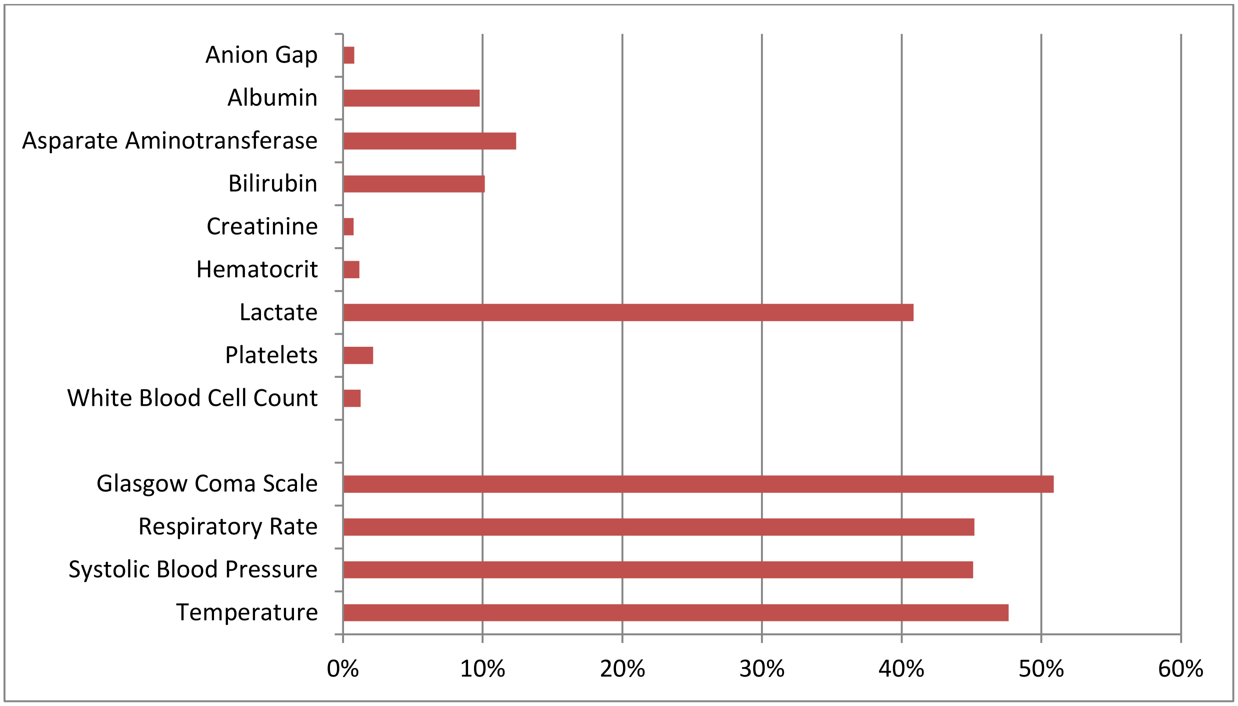

Note: missingness refers to whether or not covariates were measured within the 1st two days of hospital admission. Vital sign missingness was higher than expected as certain hospitals did not report vital signs in the database; however, vital sign availability increased in later years of the study. To address missingness, different sensitivity analyses were performed in addition to using multiple imputation (also see eTable 5).

© 2020 Rhee C et al. *JAMA Network Open.*

eTable 1. Characteristics of Sepsis Patients Who Received Adequate vs Inadequate Empiric Therapy

Characteristics	Inadequate Therapy (n=2,785)	Adequate Therapy (n=12,398)	p-value
**Median Age (IQR)**	71 (60-83)	68 (56-80)	<0.001
**Sex**			0.73
**Male**	1,247 (44.7%)	5,501 (44.4%)	
**Female**	1,539 (55.3%)	6,896 (55.6%)	
**Race**			<0.001
**White**	1,936 (69.5%)	9,239 (74.5%)	
**Black**	668 (24.0%)	2,174 (17.5%)	
**Other**	181 (6.5%)	985 (7.9%)	
**Select Comorbidities**			
**Cancer (Solid, Mets, Lymph)**	244 (8.8%)	1,042 (8.4%)	0.54
**Chronic Lung Disease**	717 (25.8%)	2,361 (19.0%)	<0.001
**Congestive Heart Failure**	676 (24.3%)	2,334 (18.8%)	<0.001
**Diabetes**	921 (33.1%)	3,801 (30.7%)	0.01
**Liver Disease**	134 (4.8%)	783 (5.9%)	0.03
**Neurologic Disease**	635 (22.8%)	2,127 (17.2%)	<0.001
**Peripheral Vascular Disease**	248 (8.9%)	855 (6.9%)	<0.001
**Renal Disease**	632 (22.7%)	2,345 (18.9%)	<0.001
**AHRQ Elixhauser Score**	13 (5-21)	11 (2-19)	<0.001
**Infectious Syndrome**			
**Pulmonary**	3,063 (32.4%)	1,868 (32.6%)	0.85
**Urinary**	4,057 (42.9%)	3,232 (56.4%)	<0.001
**Intra-abdominal**	1,347 (14.3%)	696 (12.1%)	<0.001
**Skin/Soft Tissue**	1,154 (12.2%)	427 (7.5%)	<0.001
**Bone/Joint**	397 (4.2%)	141 (2.5%)	<0.001
**Central Nervous System**	125 (1.3%)	43 (0.8%)	0.001
**Obstetric/Gynecologic**	63 (0.7%)	25 (0.4%)	0.07
**Other**	2,728 (28.9%)	1,689 (29.5%)	0.45
**Sepsis Organ Dysfunction**			
**Vasopressors**	762 (27.4%)	4,168 (33.6%)	<0.001
**Mechanical ventilation**	632 (22.7%)	2,588 (20.9%)	0.03
**Renal**	1,480 (53.1%)	6,465 (52.2%)	0.34
**Lactate**	1,158 (41.6%)	5,485 (44.2%)	0.01
**Hepatic**	222 (8.0%)	1,455 (11.7%)	<0.001
**Platelets**	272 (9.8%)	1,583 (12.8%)	<0.001
**Median SOFA Score on Admission**	4 (2-7)	4 (2-7)	0.001
**Hospital LOS**	9 (6-14)	8 (5-13)	<0.001
**Admitted to ICU**	1,248 (44.8%)	5,710 (46.1%)	0.23
**ICU LOS**	4 (3-7)	4 (3-7)	0.47
**In-Hospital Death**	488 (17.5%)	2,011 (16.2%)	0.09

© 2020 Rhee C et al. *JAMA Network Open.*

eTable 2. Characteristics of Sepsis Patients Who Received Unnecessarily Broad vs Not Unnecessarily Broad Empiric Therapy

Characteristics	Unnecessarily Broad (n=8,405)	Not Unnecessarily Broad (n=3,993)	p-value
**Median Age (IQR)**	67 (55-79)	71 (58-82)	<0.001
**Sex**			<0.001
**Male**	3989 (47.5%)	1,513 (37.9%)	
**Female**	4,416 (52.5%)	2,480 (62.1%)	
**Race**			<0.001
**White**	6,186 (73.6%)	3,053 (76.5%)	
**Black**	1,552 (18.5%)	622 (15.6%)	
**Other**	667 (7.9%)	318 (8.0%)	
**Select Comorbidities**			
**Cancer (Solid, Mets, Lymph)**	771 (9.2%)	271 (6.8%)	<0.001
**Chronic Lung Disease**	1,484 (17.7%)	877 (22.0%)	<0.001
**Congestive Heart Failure**	1,572 (18.7%)	762 (19.1%)	0.61
**Diabetes**	2,558 (30.4%)	1,243 (31.1%)	0.43
**Liver Disease**	508 (6.0%)	224 (5.6%)	0.34
**Neurologic Disease**	1,381 (16.4%)	746 (18.7%)	0.002
**Peripheral Vascular Disease**	575 (6.8%)	280 (7.0%)	0.73
**Renal Disease**	1,573 (18.7%)	772 (19.3%)	0.41
**AHRQ Elixhauser Score**	11 (2-19)	11 (3-18)	0.110
**Infectious Syndrome**			
**Pulmonary**	2,678 (31.9%)	1,228 (30.8%)	0.21
**Urinary**	3,528 (42.0%)	2,295 (57.5%)	<0.001
**Intra-abdominal**	1,166 (13.9%)	417 (10.4%)	<0.001
**Skin/Soft Tissue**	1,019 (12.1%)	279 (7.0%)	<0.001
**Bone/Joint**	338 (4.0%)	82 (2.1%)	<0.001
**Central Nervous System**	122 (1.5%)	27 (0.7%)	<0.001
**Obstetric/Gynecologic**	59 (0.7%)	16 (0.4%)	0.04
**Other**	2,397 (28.5%)	1,124 (28.2%)	0.67
**Sepsis Organ Dysfunction**			
**Vasopressors**	3,310 (39.4%)	858 (21.5%)	<0.001
**Mechanical ventilation**	1,987 (23.6%)	601 (15.1%)	<0.001
**Renal**	4,247 (50.5%)	2,218 (55.6%)	<0.001
**Lactate**	4,068 (48.4%)	1,417 (35.5%)	<0.001
**Hepatic**	1,033 (12.3%)	422 (10.6%)	0.005
**Platelets**	1,182 (14.1%)	401 (10.0%)	<0.001
**Median SOFA Score on Admission**	5(3-7)	4 (2-6)	<0.001
**Hospital LOS**	9 (6-14)	7 (5-11)	<0.001
**Admitted to ICU**	4,276 (50.9%)	1,434 (35.9%)	<0.001
**ICU LOS**	4 (3-7)	4 (3-6)	<0.001
**In-Hospital Death**	1,575 (18.7%)	436 (10.9%)	<0.001

© 2020 Rhee C et al. *JAMA Network Open.*

eTable 3. Univariate and Multivariable Models Assessing Associations Between Inadequate or Unnecessarily Broad Empiric Antibiotic Therapy and In-Hospital Death

Covariate	Univariate Model [a]		Model Assessing Inadequate Therapy[b]		Model Assessing Unnecessarily Broad Therapy[c]	
	Univariate OR [95% CI]	p-value	Multivariable OR [95% CI]	p-value	Multivariable OR [95% CI]	p-value
Age	1.01 [1.01-1.01]	<0.001	1.02 [1.02-1.02]	<0.001	1.03 [1.02-1.03]	<0.001
Female Sex	1.08 [0.99-1.18]	0.08	1.12 [1.00-1.25]	0.04	1.13 [1.00-1.28]	0.05
Race		<0.001				
White	Referent		Referent	-	-	-
Black	1.29 [1.16-1.43]		1.08 [1.04-1.12]	<0.001	1.16 [0.99-1.37]	0.07
Other	0.91 [0.76-1.07]		0.81 [0.77-0.85]	<0.001	0.82 [0.65-1.04]	0.10
AHRQ Elixhauser Index	1.03 [1.02-1.03]	<0.001	1.01 [1.01-1.02]	<0.001	1.01 [1.01-1.02]	<0.001
Positive Culture Site						
Blood Culture	1.24 [1.13-1.35]	<0.001	1.02 [0.89-1.16]	0.83	0.93 [0.79-1.08]	0.35
Body Fluid	0.94 [0.78-1.13]	0.50	-	-	-	-
CNS	1.75 [0.91-3.37]	0.010	1.69 [0.75-3.81]	0.20	1.91 [0.83-4.38]	0.13
Deep Culture	0.66 [0.40-1.08]	0.010	0.73 [0.40-1.30]	0.28	0.44 [0.21-0.93]	0.03
Other Culture	1.08 [0.70-1.65]	0.74	-	-	-	-
Respiratory Culture	2.01 [1.18-2.22]	<0.001	0.68 [0.57-0.81]	<0.001	0.67 [0.55-0.82[	<0.001
Superficial	0.62 [0.53-0.74]	<0.001	0.68 [0.54-0.86]	0.001	0.59 [0.45-0.78]	<0.001
Urine	0.69 [0.63-0.75]	<0.001	1.21 [1.04-1.41]	0.02	1.26 [1.05-1.51]	0.01
Pathogen						
Acinetobacter	1.14 [0.76-1.71]	0.52	-	-	-	-
Citrobacter	1.29 [0.92-1.81]	0.14	-	-	-	-
E.coli	0.65 [0.59-0.72]	<0.001	0.77 [0.66-0.89]	<0.001	0.78 [0.66-0.93]	0.004
Enterobacter	1.09 [0.85-1.39]	0.51	-	-	-	-
Klebsiella	1.17 [1.03-1.33]	0.01	1.11 [0.93-1.32]	0.23	1.18 [0.97-1.44]	0.10
Serratia	1.05 [0.72-1.53]	0.81	-	-	-	-
Proteus	0.86 [0.71-1.04]	0.12	-	-	-	-
P.aeruginosa	1.23 [1.06-1.43]	0.008	1.13 [0.90-1.44]	0.30	1.22 [0.90-1.67]	0.20
S.aureus	1.49 [1.35-1.64]	<0.001	1.53 [1.29-1.80]	<0.001	1.63 [1.34-1.98]	<0.001
Streptococcus	1.10 [0.98-1.25]	0.09	0.94 [0.79-1.13]	0.52	1.01 [0.83-1.23]	0.91
Enterococcus	1.08 [0.94-1.23]	0.28	-	-	-	-
Antibiotic-Resistant Pathogen	1.33 [1.21-1.47]	<0.001	1.06 [0.90-1.24]	0.51	1.11 [0.91-1.35]	0.30
Infectious Syndrome						
Bone/Joint	0.41 [0.30-0.56]	<0.001	0.72 [0.50-1.04]	0.08	0.85 [0.57-1.27]	0.44
CNS	0.97 [0.64-1.47]	0.89	-	-	-	-
Genitourinary	0.46 [0.42-0.51]	<0.001	0.55 [0.48-0.64]	<0.001	0.52 [0.45-0.61]	<0.001
Intra-abdominal	0.87 [0.76-0.99]	0.03	0.67 [0.57-0.79]	<0.001	0.66 [0.55-0.80]	<0.001
OB/GYN	0.72 [0.38-1.36]	0.32	-	-	-	-
Other	0.41 [0.37-0.46]	<0.001	0.56 [0.49-0.64]	<0.001	0.57 [0.49-0.67]	<0.001
Pulmonary	1.54 [1.41-1.69]	<0.001	1.12 [0.99-1.27]	0.06	1.08 [0.94-1.25]	0.26
Skin/Soft Tissue	0.5 [0.50-0.70]	<0.001	0.82 [0.67-1.01]	0.06	0.90 [0.71-1.13]	0.35
ICU on Admission	2.47 [2.26-2.69]	<0.001	0.93 [0.82-1.05]	0.26	0.90 [0.79-1.04]	0.15

(Table continues on next page)

© 2020 Rhee C et al. *JAMA Network Open.*

Covariate	Univariate Model [a]		Model Assessing Inadequate Therapy[b]		Model Assessing Unnecessarily Broad Therapy[c]	
	Univariate OR [95% CI]	p-value	Multivariable OR [95% CI]	p-value	Multivariable OR [95% CI]	p-value
Severity of Illness						
Albumin (min)	0.37 [0.35-0.40]	<0.001	0.55 [0.50-0.61]	<0.001	0.56 [0.50-0.62]	<0.001
AST (max)	1.00 [1.00-1.00]	<0.001	1.00 [1.00-1.00]	0.14	1.00 [1.00-1.00]	0.19
Anion Gap (max)	1.10 [1.09-1.11]	<0.001	1.02 [1.01-1.04]	<0.001	1.02 [1.00-1.03]	0.008
Creatinine (max)	1.10 [1.08-1.12]	<0.001	1.02 [0.99-1.05]	0.18	1.03 [0.99-1.06]	0.13
GCS (min)	0.81 [0.80-0.82]	<0.001	0.94 [0.92-0.95]	<0.001	0.93 [0.91-0.96]	<0.001
Hematocrit (min)	0.97 [0.97-0.98]	<0.001	1.01 [1.00-1.02]	0.003	1.01 [1.00-1.02]	0.01
Lactate (max)	1.36 [1.32-1.37]	<0.001	1.13 [1.11-1.56]	<0.001	1.14 [1.11-1.17]	<0.001
Mech Ventilation	4.86 [4.43-5.32]	<0.001	2.14 [1.85-2.49]	<0.001	2.18 [1.84-2.59]	<0.001
Platelet count (min)	1.00 [1.00-1.00]	<0.001	1.00 [1.00-1.00]	0.007	1.00 [1.00-1.00]	0.02
Resp Rate (max)	1.02 [1.02-1.03]	<0.001	1.00 [1.00-1.01]	0.03	1.00 [1.00-1.01]	0.08
Total Bilirubin (max)	1.10 [1.08-1.12]	<0.001	1.06 [1.04-1.09]	<0.001	1.07 [1.05-1.10]	<0.001
Sys Bld Press (min)	0.96 [0.96-0.96]	<0.001	0.99 [0.99-1.00]	<0.001	0.99 [0.99-1.00]	0.01
Temperature (max)	0.89 [0.86-0.91]	<0.001	0.93 [0.89-0.96]	<0.001	0.93 [0.89-0.96]	<0.001
Vasopressor	4.59 [4.19-5.02]	<0.001	2.02 [1.77-2.30]	<0.001	2.10 [1.81-2.43]	<0.001
WBC (max)	1.01 [1.01-1.01]	<0.001	1.00 [1.00-1.01]	0.25	1.00 [1.00-1.01]	0.17
Inadequate Empiric Therapy	1.10 [0.98-1.22]	0.09	1.19 [1.03-1.37]	0.02	-	-
Unnecessarily Broad Therapy	1.88 [1.68-2.11]	<0.001	-	-	1.22 [1.06-1.40]	0.007

Abbreviations: CNS = central nervous system, ICU = intensive care unit, AST = asparate aminotransferase, GCS = Glasgow Coma Scale, Mech Ventilation = mechanical ventilation, Resp Rate = Respiratory Rate, Sys Bld Press = systolic blood pressure, WBC = white blood cell count.

[a] Univariate models show the association between each covariate and in-hospital death among all culture-positive community-onset sepsis patients in the cohort (n=17,430). Admission year and hospital characteristics (bed size, region, teaching status) were significant at p<0.001 in the univariate screens but are not shown in the table.

[b] Multivariable model 1 shows the full model assessing the association between inadequate empiric antibiotic therapy (at least one organism recovered from a clinical culture site that was non-susceptible to all administered empiric antibiotics) and in-hospital death. This analysis was performed among culture-positive sepsis patients in whom each antibiotic-pathogen combination could be either directly measured or imputed (n=15,183).

[c] Multivariable model 2 shows the full model assessing the association between unnecessarily broad empiric antibiotic therapy and in-hospital death. This model was done among culture-positive sepsis patients who received adequate empiric antibiotic therapy and in whom each antibiotic-pathogen combination could be either directly measured or imputed (n=12,398). Unnecessarily broad empiric therapy indicates patients who received anti-MRSA (vancomycin, linezolid, daptomycin), anti-VRE (linezolid or daptomycin), anti-Pseudomonal beta-lactams (ceftazidime, cefepime, piperacillin-tazobactam, aztreonam, imipenem, meropenem, doripenem), or anti-ESBL therapy (any carbapenem) when none of those resistant organisms were isolated.

© 2020 Rhee C et al. *JAMA Network Open.*

eTable 4. Distribution of Values for Severity-of-Illness Covariates in Culture-Positive Sepsis Patients

Covariate	Mean (SD	Median (IQR)	Units
Laboratory			
Anion Gap (max)	14.0 (5.5)	13.0 (10.0-16.3)	mEq/L
Albumin (min)	2.7 (0.7)	2.7 (2.2-3.7)	g/dL
Aspartate Aminotransferase (max)	155.2 (732.4)	37 (23-75)	units/L
Bilirubin (max)	1.5 (2.2)	0.8 (0.5-1.5)	mg/dL
Creatinine (max)	2.3 (2.0)	1.7 (1.1-2.7)	mg/dL
Hematocrit (min)	31.3 (6.5)	31.3 (27.0-35.6)	%
Lactate (max)	3.6 (3.3)	2.6 (1.8-4.1)	mmol/L
Platelet Count (min)	193.8 (113.7)	176 (117-249)	10^9 cells/L
White Blood Cell Count (max)	17.0 (12.4)	15.0 (10.4-21.0)	10^9 cells/L
Vital Signs or Mental Status			
Glasgow Coma Scale (min)	11.6 (4.2)	14 (8-15)	-
Respiratory Rate (max)	32.0 (14.6)	28 (22-36)	breaths/min
Systolic Blood Pressure (min)	85.1 (21.5)	85 (70-99)	mmHg
Temperature (max)	100.4 (2.0)	100.1 (98.8-101.8)	°Farenheit

Note: the most abnormal (minimum or maximum) values of covariates within the first 2 days of hospitalization were used.

© 2020 Rhee C et al. *JAMA Network Open.*

eTable 5. Sensitivity Analyses for Multivariable Models for In-Hospital Death Using Different Strategies to Account for Missing Severity-of-Illness Covariates

Empiric Antibiotic Therapy Pattern	Strategy 1: Median Value Imputation	Strategy 2: Multiple Imputation, No missing vital signs	Strategy 3: No Missing Covariates
	**OR [95% CI] (p-value**	**OR [95% CI] (p-value)**	**OR [95% CI] (p-value)**
**Inadequate Empiric Therapy**	1.17 [1.03-1.34] (p=0.02)	1.22 [1.02-1.48] (p=0.04)	1.19 [1.00-1.42] (p=0.06)
**Unnecessarily Broad Therapy**	1.21 [1.06-1.39] (p=0.006)	1.26 [1.04-1.53] (p=0.02)	1.20 [0.93-1.55] (p=0.15)

Inadequate empiric therapy indicates patients who had at least organism recovered from a clinical culture site that was non-susceptible to all administered empiric antibiotics. Unnecessarily broad empiric therapy indicates patients who received anti-MRSA, anti-VRE, anti-Pseudomonal, or anti-ESBL therapy when none of those resistant organisms were isolated. Anti-MRSA agents included vancomycin, linezolid, and daptomycin; anti-VRE agents included linezolid and daptomycin; anti-Pseudomonal beta-lactams included ceftazidime, cefepime, piperacillin-tazobactam, imipenem, meropenem, and doripenem; anti-ESBL agents included imipenem, meropenem, doripenem, and ertapenem..

All models were adjusted for age, sex, race, AHRQ Elixhauser comorbidity score, site of positive clinical cultures, pathogen, presence of a resistant organism (MRSA, VRE, Ceftriaxone-resistant gram-negative organism, ESBL, or CRE), severity of illness on admission (ICU care, vasopressors, mechanical ventilation, serum lactate, creatinine, total bilirubin, white blood cell count, albumin, platelet count, hematocrit, anion gap, aspartate aminotransferase, temperature, systolic blood pressure, respiratory rate, and Glasgow Coma Scale), admission year, and hospital characteristics (region, bed size, and teaching status).

Strategy 1 for handling missing data imputed median values. Strategy 2 was limited to patients with no missing vital signs on admission, and used multiple imputation to account for laboratory covariates and Glasgow Coma Scale. Strategy 3 was limited to patients with no missing covariates. Each strategy yielded a different sample size, as below:

Inadequate empiric therapy analysis:

- Strategy 1: n=15,183 (2,499 deaths)
- Strategy 2: n=7,937 (1,369 deaths)
- Strategy 3: n=4,516 (931 deaths)

Unnecessarily broad empiric therapy analysis:

- Strategy 1: n=12,398 (2,011 deaths)
- Strategy 2: n=6,222 (1,052 deaths)
- Strategy 3: n=3,545 (727 deaths)

© 2020 Rhee C et al. *JAMA Network Open.*
